# Reduced MGE burden, virulence optimization, and acid stress tolerance shape the clonal succession of MRSA ST59

**DOI:** 10.1126/sciadv.aeb3121

**Published:** 2026-03-11

**Authors:** Ye Jin, Wangxiao Zhou, Xu Dong, Qi Ge, Pan Chen, Yongchang Xu, Ping Shen, the BRICS Working Group, Beiwen Zheng, Yonghong Xiao

**Affiliations:** ^1^ State Key Laboratory for Diagnosis and Treatment of Infectious Diseases, National Clinical Research Center for Infectious Diseases, Collaborative Innovation Center for Diagnosis and Treatment of Infectious Diseases, the First Affiliated Hospital, Zhejiang University School of Medicine, Hangzhou 310000, China.; ^2^ Department of General Intensive Care Unit, The Second Affiliated Hospital of Zhejiang University School of Medicine, Hangzhou 310000, China.; ^3^ Clinical Laboratory Center, The Second Affiliated Hospital & Yuying Children’s Hospital of Wenzhou Medical University, Wenzhou 325000, China.; ^4^ Peking Union Medical College & Institute of Pathogen Biology, Chinese Academy of Medical Sciences & Research Units of Infectious Disease and Microecology, Chinese Academy of Medical Sciences, Beijing 100871, China.; ^5^ Department of Immunology and Pathogen Biology, School of Basic Medical Sciences, Hangzhou Normal University, Hangzhou 310000, China.

## Abstract

Methicillin-resistant *Staphylococcus aureus* (MRSA) ST59 clone has replaced the historically dominant ST239 in China. However, the mechanisms behind this shift remain elusive. Phylogenetic analysis revealed divergent evolutionary trajectories: ST239 emerged via interlineage recombination and accumulated extensive resistance and genome degradation, whereas ST59 followed a more conserved, recombination-sparse path marked by reduced mobile genetic element (MGE) burden and retention of key virulence–associated MGEs. ST239’s decline was attributed to the fitness costs of multidrug resistance, widespread pseudogenization, and a closed pan-genome. Furthermore, ST59 exhibited enhanced virulence. Two key virulence determinants were identified: deletion of *chp* impaired survival in whole blood and neutrophil cocultures by disrupting C5a-mediated chemotaxis; *sraP* deletion reduced epithelial adhesion, nasal colonization, and systemic virulence. ST59 also demonstrated superior acid tolerance, linked to transcriptomic activation of transport and metabolic pathways. Our findings highlight that the epidemiological success of clones requires a balance of reduced resistance, targeted virulence, and environmental adaptability.

## Teaser

Epidemiological success of MRSA ST59 reflects reduced resistance burden, targeted virulence, and enhanced environmental tolerance.

## INTRODUCTION

In recent years, reconstruction of the evolutionary history of major pathogenic *Staphylococcus aureus* (*S. aureus*) clones, particularly methicillin-resistant *S. aureus* (MRSA), has provided remarkable insights into their emergence, expansion, and dissemination. For example, in the United Kingdom, the EMRSA15 (ST22) and EMRSA-16 (ST36) clones dominated the landscape of hospital-associated MRSA (HA-MRSA) strains during the 1990s and 2000s, each following distinct trajectories of emergence and decline (*1*). In North America, the rise of the community-acquired (CA)-MRSA USA300 clone has been linked to increased morbidity and mortality, substantially affecting clinical management and public health strategies (*2*). These global examples illustrate how shifts in dominant MRSA clones can lead to major changes in disease burden, treatment effectiveness, and infection control practices. In this context, understanding the epidemic dynamics of emerging clones within specific regions is critical for anticipating clinical challenges and informing regionally tailored intervention strategies.

Numerous genomic and epidemiological studies have shown that the dominant MRSA clones follow similar patterns of emergence, expansion, equilibrium, and decline in their evolutionary history (*1*, *3*). However, a reasonable explanation for the population expansion of these dominant clones has been provided in very few instances. In some cases, clones may have acquired antibiotic resistance, specific virulence factors, or increased adaptability to both the host and the environment during their evolutionary process (*4*–*6*). The USA300 lineage carries the arginine catabolic mobile element, which encodes an arginine deiminase system (Arc) that facilitates bacterial survival in acidic environments resembling the human skin, thereby promoting colonization and transmission (*7*). Moreover, mutations in global regulatory genes such as *sarZ* have been shown to increase the virulence of USA300 bloodstream infection (BSI) isolates, underscoring the importance of regulatory rewiring in the evolution of pathogenicity (*8*). These studies shed light on the genetic diversity and evolution of these pathogens. However, our understanding of the specific traits that define a successful clone and contribute to its persistence and spread remains limited.

In this study, we report a notable clonal replacement event in which MRSA ST59 has progressively supplanted the historically dominant ST239 lineage among BSI isolates across China over the past decade. To investigate the evolutionary basis of this shift, we conducted a nationwide, longitudinal genomic epidemiology study of MRSA bloodstream isolates collected between 2011 and 2020. Rather than being driven by expanded antimicrobial resistance (AMR), the epidemiological success of ST59 appeared to result from the synergistic contribution of enhanced immune evasion, increased tolerance to acidic and thermal stress, and the retention of the lineage-specific virulence determinants *chp* and *sraP*.

## RESULTS

### Distinct evolutionary origins and mobile genetic element remodeling underpin the BSI MRSA clonal shift in China

**Fig. 1.**
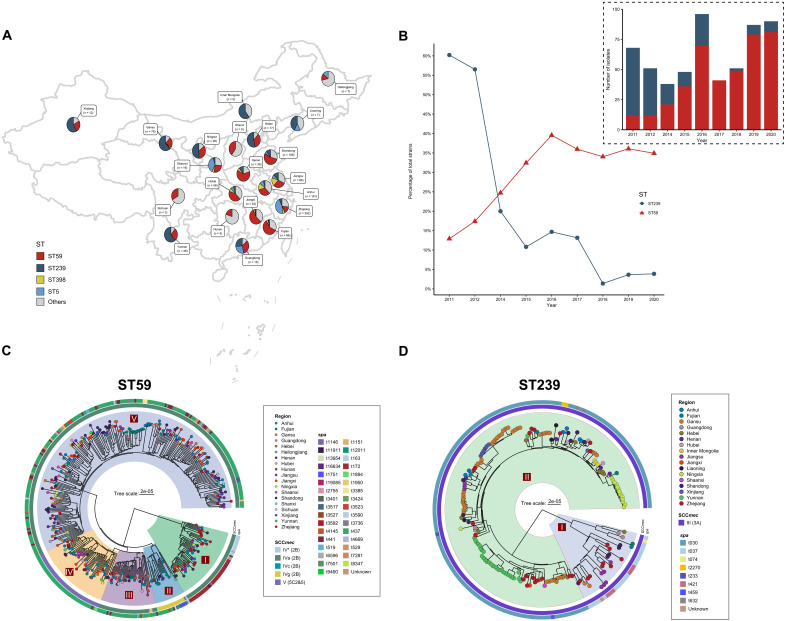
Dynamic population structure changes in MRSA from bloodstream infections in China. (**A**) Geographical distribution of the 1244 MRSA isolates collected from 72 sentinel hospitals that participated in this study. Each pie chart shows the distribution of STs in the corresponding region; the adjacent label indicates the number of isolates. (**B**) The annual counts (top) and percentage of total isolates (bottom) detected each year from 2011 to 2020. Percentages are calculated relative to all MRSA strains in the surveillance dataset for that year. (**C** and **D**) Phylogeny of ST59 (*n* = 401) and ST239 (*n* = 184) in this study, respectively. Tree tips are colored by region of isolation. SCC*mec* (inner ring) and *spa* (outer ring) types are annotated on the tree.

Between 2011 and 2020, a total of 1244 nonduplicate MRSA isolates (table S1 and Fig. 1A) were collected from BSIs in 72 sentinel hospitals spanning 22 provinces across China, representing approximately 75.7% of the national population (ca. 1.06 billion). Multilocus sequence typing (MLST) identified 15 clonal complexes (CCs) and 90 STs among these isolates. The predominant lineages were ST59 (*n* = 401, 32.23%), ST239 (*n* = 184, 14.79%), ST5 (*n* = 227, 18.25%), and ST398 (*n* = 75, 6.03%), while the remaining isolates were distributed across multiple STs, each representing <3% of the total collection (table S1). To assess population dynamics, the number of isolates belonging to each lineage was counted annually at the national level. This analysis revealed a marked shift in BSI MRSA clonal composition over the 10-year period: ST239, which was the dominant lineage before 2011, progressively declined in prevalence and was overtaken by ST59 starting in 2014 (Fig. 1B). This trend was observed across multiple hospitals nationwide, indicating a widespread clonal replacement event that culminated in the emergence of ST59 as the predominant MRSA lineage in China.

To investigate the evolutionary dynamics underlying this transition, we reconstructed the phylogenetic histories of the two major MRSA lineages and compared their genome architectures. Bayesian inference based on core-genome SNPs revealed that ST239 (*n* = 184) and ST59 (*n* = 401) originated around the same period, with their most recent common ancestors dating to approximately 1938.26 for ST239 (95% confidence interval: 1923.9 to 1950.7) and 1941.7 for ST59 (95% confidence interval: 1928.2 to 1952.9) (Fig. 1, C and D, and fig. S1). Despite their temporal overlap, the two lineages differ substantially in their genetic origins (fig. S2).

**Fig. 2.**
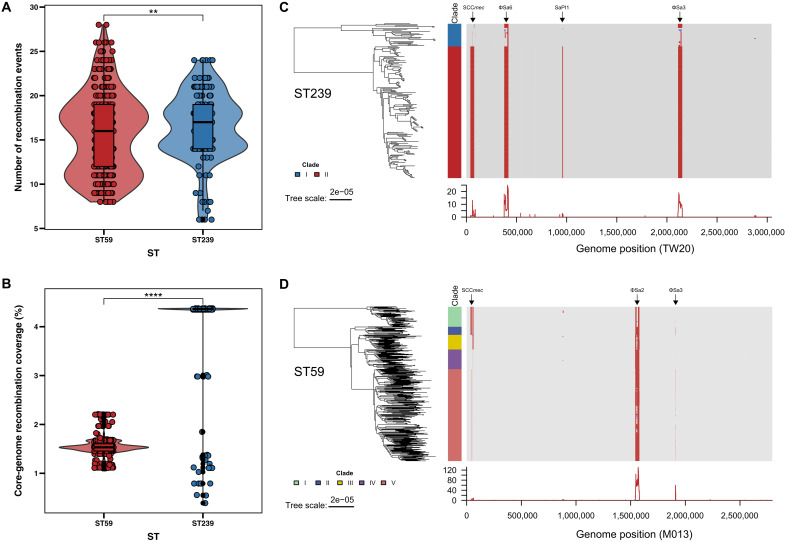
Within-lineage recombination in ST239 (*n* = 184) and ST59 (*n* = 401). (**A**) Distribution of recombination event counts within the ST239 and ST59 MRSA lineages. Two-sided Mann-Whitney *U* test (ST59 versus ST239). (**B**) Distribution of core-genome recombination coverage within the ST239 and ST59 MRSA lineages. Two-sided Mann-Whitney *U* test (ST59 versus ST239). **P < 0.01; ****P < 0.0001. (**C** and **D**) Genome-wide recombination landscape inferred with Gubbins for ST239 (C, mapped to reference TW20) and ST59 (D, mapped to reference M013). For each lineage, a maximum likelihood phylogeny is shown with clade annotations, and the adjacent heatmap depicts inferred recombination tracts. Line plots below the heatmaps show the frequency of recombination events along the reference genome.

To trace their respective genetic origins, we analyzed the ST239 lineage. Consistent with previous reports (*9*, *10*), our analysis of 184 China-derived ST239 genomes confirmed that ST239 is a mosaic lineage composed of large chromosomal segments from ST8 and ST30, with breakpoints near *ulaA* and *farE* (fig. S2, A to D). By contrast, across 401 ST59 genomes in this study, we found no evidence of large-scale interlineage chromosomal recombination, with recombination signals confined to MGEs (fig. S2, E to G). Furthermore, we quantified within-lineage recombination during the study period. ST239 exhibited a higher recombination-to-mutation ratio than ST59 [r/m, 0.78 (95% CI: 0.76 to 0.81) versus 0.60 (95% CI: 0.59 to 0.61)], a greater number of recombination events per genome (mean: 16.5 versus 15.7, *P* < 0.01) and greater core-genome recombination coverage (mean: 4.36% versus 1.53%, *P* < 0.0001) (Fig. 2, A and B). In both lineages, recombination tracts localized predominantly to MGE-rich regions, including SCC*mec*, φSa3, φSa6, SaPI1, and φSa2 (Fig. 2, C and D).

Consistent with their divergent origins, the two lineages followed markedly different evolutionary trajectories. As shown in Fig. 1D, ST239 segregated into two principal clades (ST239-I and ST239-II). Molecular dating in our analysis placed the MRCA of ST239-II approximately 29 years after that of ST239-I. Consistent with this sublineage turnover, ST239-I was predominantly *spa* t037 (70.37%, 19 of 27), whereas ST239-II was predominantly t030 (96.18%, 151 of 157), in agreement with Harris *et al.* (*11*) identifying t037 as ancestral and with the report that t030 has replaced t037 in China by the late 2000s (*12*). In our 2011–2020 collection, ST239-II accounted for >85% of ST239 isolates and exhibited notable genome reduction compared with ST239-I (fig. S3, A and B). This contraction was primarily driven by the loss of large MGEs, including φSPβ, φSa6, and φSa2 (figs. S3 and S4), and a concomitant acquisition of smaller SaPI2/3/4-like elements (~14 to 16 kb) lacking characterized virulence factors (fig. S5). In contrast, ST59 displayed greater phylogenetic diversification, consisting of five distinct clades (I to V) defined by lineage-specific SCC*mec* and *spa* types (Fig. 1C), alongside a more compact genome with substantially reduced MGE burden (fig. S3, C and D). Notably, ST59 lacked several MGEs present in ST239, including φSPβ, SaPIs, and transposons Tn*554*/Tn*6072* (fig. S4), but retained critical virulence determinants such as *chp*, *seb*, and *sey* (fig. S6), many of which were lineage-specific and implicated in immune evasion. Together, these findings indicate that ST239 experienced genomic attrition characterized by large-scale MGE loss and functional erosion, whereas ST59 exhibited reduced MGE burden, selectively retaining a minimal yet potent set of virulence-associated elements, thereby facilitating its epidemiological expansion and dominance among BSI isolates.

### Resistance burden, genomic degeneration, and a pan-genome closure trend may contribute to the decline of MRSA ST239 among BSI isolates

Previous studies have proposed that the acquisition and maintenance of AMR genes constitute a key evolutionary force underpinning the emergence and persistence of epidemic MRSA lineages such as ST239, particularly in hospital environments where antibiotic pressure is high (*1*, *13*). However, the factors associated with the subsequent decline and clonal replacement of such lineages remain incompletely understood. To investigate whether differential resistance burdens might underlie the observed replacement of ST239 by ST59 among BSI isolates in our cohort, we performed a comprehensive comparative genomic analysis of resistance gene content and positive selection signatures across both lineages.

**Fig. 3.**
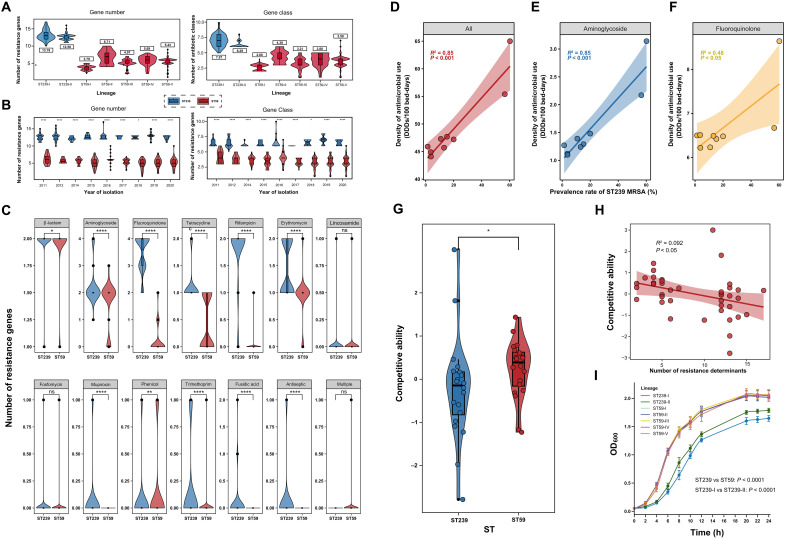
Comparative resistance profiles, fitness deficits, and the impact of antibiotic pressure on ST239 and ST59 prevalence. (**A**) Distribution of number of AMR genes and antibiotic classes among clades within ST239 (*n* = 184) and ST59 (*n* = 401). (**B**) Distribution of resistance gene numbers and resistance gene classes carried by the ST239 and ST59 over the study period. The numbers within the boxes for each clade in the figure represent the average number of resistance genes or the average number of resistance gene classes per strain in that clade. Two-sided Mann-Whitney *U* test (ST59 versus ST239). (**C**) Distribution of resistance gene numbers among different classes within the ST239 (*n* = 184) and ST59 (*n* = 401). This figure illustrates the distribution of resistance gene numbers carried by the ST239 and ST59 MRSA lineages across 14 different classes of resistance gene. Two-sided Mann-Whitney *U* test (ST59 versus ST239). (**D** to **F**) The correlation between antimicrobial usage intensity and the prevalence of ST239. Figure depicts the relationships between ST239 MRSA prevalence and the usage intensity of total antibiotics (D), aminoglycosides (E), and fluoroquinolones (F), respectively, from 2011 to 2020. Linear regression analyses show the *R*^2^ and *P* values for each relationship, with 95% confidence intervals indicated by shaded areas around the regression lines. (**G**) Comparison of competitive ability between ST239 (*n* = 20) and ST59 (*n* = 20). Plotted points show the competitive ability of *S. aureus* isolates. Statistical significance was calculated using the two-sided Mann-Whitney *U* test. (**H**) Relationship between the number of resistance genes and the competitive ability of strains. Linear regression analysis shows the *R*^2^ and *P* value for this relationship; the shaded area denotes the 95% confidence interval around the fitted line. (**I**) Growth curves of ST59 (*n* = 20) and ST239 (*n* = 20). *P < 0.05, **P < 0.01, and ****P < 0.0001; ns, no significant difference.

ST239 isolates harbored more resistance genes (mean: 12.60 versus 5.27) and spanned more resistance gene classes (mean: 6.40 versus 3.44) compared to ST59 (both *P* < 0.0001), indicating a broader and more complex resistance repertoire (Fig. 3, A and B). ST239 exhibited higher resistance across almost antibiotic classes, apart from lincosamides, fosfomycin, phenicols, and multiclass resistance genes, including rifampin, tetracyclines, aminoglycosides, and fluoroquinolones (Fig. 3E and table S2). These patterns were stable throughout the 10-year surveillance period, during which ST239 consistently maintained a higher average resistance burden compared to ST59 (Fig. 3, C and D).

To directly examine potential trade-offs associated with resistance accumulation, we conducted growth and competition assays using 20 representative isolates from each lineage. ST59 isolates consistently outcompeted ST239 in coculture (*P* < 0.05, Fig. 3G), and a substantial negative correlation was observed between resistance gene number and competitive ability (*R*^2^ = 0.092 and *P* < 0.05; Fig. 3H). Growth curve analysis further confirmed that ST59 had a notably faster growth rate than ST239 (mean maximum rate: 0.64 versus 0.44, *P* < 0.001; Fig. 3I). These differences were supported by evolutionary signals: ST239 harbored 32 positively selected core genes, including and involved in resistance (e.g., *grlA*, *rpoB*, and *ileS*; fig. S7), consistent with strong antibiotic-driven selection. In contrast, ST59 had 51 positively selected genes, but the vast majority (92.16%) were associated with metabolism and environmental adaptation (figs. S7 and S8), reflecting a divergent evolutionary path toward enhanced metabolic plasticity.

To further investigate ST239’s fitness limitations, we analyzed genome degradation. ST239 isolates exhibited more stable pseudogenization events (≥50% of strains in any clade) than ST59 (43 versus 22 genes; fig. S9, A and B). These included loss-of-function mutations in adhesion genes (*icaC*, *sasG*, and *fnbA*/*B*) and immune evasion genes (*scb* and *vWbp*), potentially impairing host colonization and persistence. Inactivation events were also enriched in key metabolic pathways, such as DNA, ion, and amino acid metabolism.

At the strain level, ST239 isolates carried significantly more pseudogenes than ST59 (mean: 40 versus 14, *P* < 0.0001; fig. S9, C and D). ST239-II accumulated more pseudogenes than ST239-I (35 versus 24), indicating genomic erosion. In contrast, ST59 maintained functional redundancy for some inactivated genes via intact homologs (e.g., *hysA*^vSaβ^ and *eap*), potentially buffering fitness loss, suggesting that in addition to resistance-related fitness costs, extensive pseudogenization may have further compromised ST239’s virulence, metabolic flexibility, and overall ecological viability.

To further assess the long-term adaptive potential of the two lineages, we next analyzed their pan-genome dynamics. ST239 exhibited a steeper pan-genome accumulation curve than ST59, indicating higher accessory gene diversity (fig. S10A). Principal components analysis (PCA) confirmed greater gene content variation in ST239 (*P* < 0.0001; fig. S10D), and ST239 genomes carried a larger proportion of accessory genes (fig. S10C). However, despite its larger gene pool, ST239’s pan-genome is transitioning toward closure. Using the Tettelin *et al.* model (*14*), ST239 had an α value >1 (α = 1.19), whereas ST59 remained below 1 (α = 0.87).

### ST59 isolates exhibit increased virulence

While the decline of ST239 can be attribute to the cumulative burden of multidrug resistance, genome degradation, and reduced adaptive flexibility, these factors alone do not totally account for the concurrent expansion of ST59. The sustained emergence of ST59 during the same period raises a critical question: What biological or ecological advantages enabled this lineage to not only persist but to outcompete and ultimately replace its predecessor in an evolving clinical and antimicrobial landscape? In contrast to ST239, whose evolutionary trajectory was shaped by prolonged antibiotic exposure and resistance accumulation, ST59 appears to have adapted along a different path. One such factor may be enhanced virulence potential. To test this, we compared the cytotoxicity of all ST59 (*n* = 401) and ST239 (*n* = 184) isolates using a standardized human monocytic cell model.

**Fig. 4.**
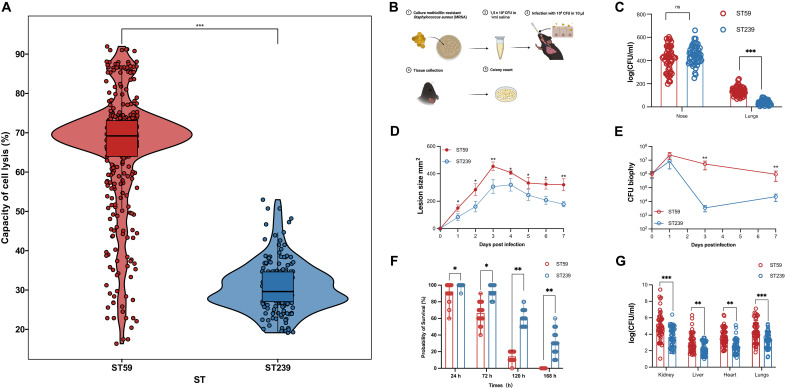
Virulence comparison between MRSA lineages ST239 and ST59. (**A**) Cytotoxicity between ST59 (*n* = 401) and ST239 (*n* = 184) isolates toward human monocytic cells in vitro, providing a standardized metric for assessing their virulence potential. Two-sided Student’s *t* test. (**B**) Schematic representation of the intranasal inoculation (nose drop) mouse model used to assess bacterial colonization and infection. Created in BioRender. Y. Jin (2026) https://BioRender.com/izu1cgr. (**C**) Comparison of bacterial loads in the nose and lungs of mice infected with ST59 and ST239 (*n* = 10 mice per strain; 50 strains per lineage) for 48 hours postinfection. Two-sided Mann-Whitney *U* test. (**D**) Nude mice were infected subcutaneously with ST59 or ST239 (*n* = 10 mice per strain; 50 strains per lineage) and developing skin infection was observed. The size of dermonecrotic lesions was measured daily. Two-sided Mann-Whitney *U* test. (**E**) On selected days, the infected areas were biopsied, and skin bacterial burden in homogenized biopsy specimens was measured. Two-sided Mann-Whitney *U* test (ST59 versus ST239). (**F**) Survival following intravenous challenge, summarized as the proportion of surviving mice at 24, 72, 120, and 168 hours for ST59 and ST239 (groups of 10 mice per strain; 50 strains per lineage). Two-sided Mann-Whitney *U* test. (**G**) Bacterial burdens in major organs of mice infected with ST59 and ST239 strains in a BSI model. Fifty representative strains each from the ST59 (red hues) and ST239 (blue hues) lineages were used to infect mice (*n* = 10 mice per strain). At the designated time point, bacterial loads in the kidney, lungs, liver, and heart were quantified. Two-sided Mann-Whitney *U* test (ST59 versus ST239). Data are presented as mean ± SD. *P < 0.05, **P < 0.01, and ***P < 0.001; ns, no significant difference.

As shown in Fig. 4A, the ST59 strains presented markedly greater cytolytic damage than the ST239 strains did (mean and median cytotoxicity of 65.93 and 69.20% for ST59; mean and median cytotoxicity of 30.74 and 29.61% for ST239; *P* < 0.001). While cytotoxicity confirms the greater cell-lytic potential of ST59, overall pathogenicity is governed by a broader set of virulence attributes. To comprehensively assess the pathogenic potential of MRSA ST59 and ST239, we established a series of in vivo murine infection models that simulate different stages of *S. aureus* pathogenesis, including local tissue invasion, mucosal colonization and dissemination, and systemic infection. Fifty representative isolates from each clonal lineage were randomly selected and tested in a murine infection model. To assess colonization and dissemination, we used a murine nasal inoculation model simulating early upper-to-lower respiratory tract transition (Fig. 4B). While nasal colonization was comparable (Fig. 4C), ST59-infected mice showed significantly higher lung bacterial loads (*P* < 0.001), indicating greater invasive capacity.

We next evaluated the tissue-invasive potential of each clonal lineage using a murine subcutaneous infection model. Three days postinoculation, mice infected with ST59 strains developed extensive skin lesions characterized by necrosis and inflammation, whereas those infected with ST239 strains exhibited only mild abscess formation (Fig. 4D), indicating markedly higher tissue-destructive potential in ST59. In ST239-infected mice, necrotic lesions developed more slowly and never reached the severity observed with ST59. During the early phase of infection, both lineages proliferated comparably, although ST239 strains showed a trend toward reduced colony-forming units (CFUs). However, by day 3, ST59 strains persisted robustly in the infected tissue, whereas bacterial loads of ST239 declined notably (Fig. 4E). These findings suggest that ST59 has enhanced capacity to evade early immune clearance, conferring a distinct advantage in tissue persistence and pathogenesis compared to ST239.

To investigate systemic virulence, we established a murine BSI model to simulate severe invasive disease. A notable difference in lethality was evident when comparing clinical ST59 and ST239 BSI isolates (Fig. 4F). When mice were injected intravenously with 1 × 10^7^ CFU/ml of bacterial suspension, the 120-hour survival rate for those infected with ST59 was markedly lower than for those infected with ST239 (≤20% versus ≥50%, respectively). By day 7 postinfection, at least 10% of the ST239-infected mice remained alive, whereas all ST59-infected mice had succumbed. To further assess bacterial dissemination, we performed a low-dose infection (1 × 10^5^ CFU/ml) model and harvested major organs on day 3 postinfection. Mice infected with ST59 exhibited considerably higher bacterial burdens in multiple organs, including the kidneys, livers, hearts, and lungs, compared to those infected with ST239 (Fig. 4G). These results collectively demonstrate that ST59 exhibits markedly enhanced systemic virulence compared to ST239, both in terms of lethality and capacity for dissemination.

In addition, within-lineage analyses showed that cytotoxicity correlated positively with skin lesion size and skin bacterial burdens in both ST59 and ST239 (*P* < 0.05), and in ST59 also correlated positively with multiorgan bacterial burdens (*P* < 0.05) and negatively with the 120-hour survival rate in the BSI model (*P* < 0.05) (fig. S11).

### *chp* and *sraP* were associated with the greater virulence of ST59

Having demonstrated that ST59 strains exhibit heightened virulence, we next investigated the genetic basis underlying these phenotypic traits. Prior studies have shown that specific virulence factors can enhance *S. aureus* adaptability to host environments by promoting immune evasion, tissue invasion, and persistence, potentially contributing to clonal success (*5*, *7*). While MRSA virulence can be inferred from genome sequences (*15*), to pinpoint potential virulence determinants, we performed a comparative analysis of virulence gene repertoires and lineage-enriched genomic elements.

**Fig. 5.**
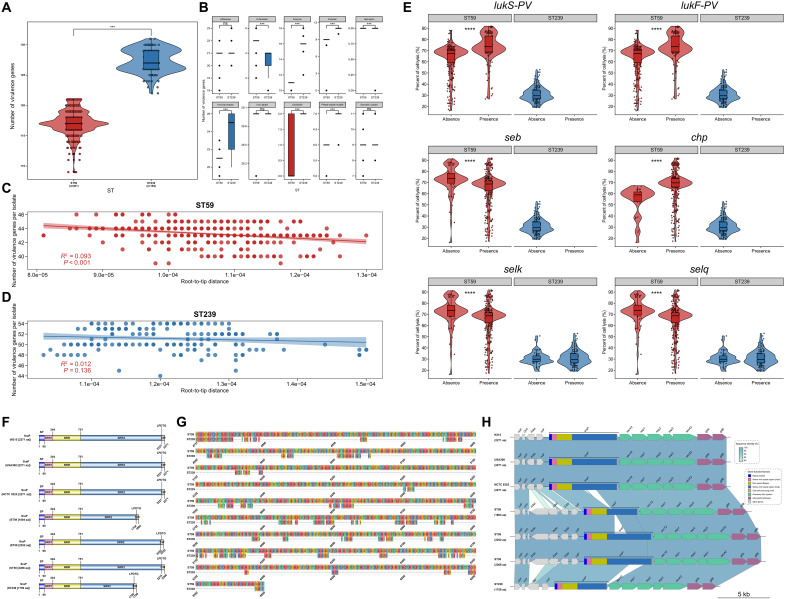
Comparative analysis of virulence gene content and the molecular basis of key virulence factors between ST239 and ST59. (**A**) Total number of virulence genes identified in the ST59 (*n* = 401) and ST239 (*n* = 184) isolates. Two-sided Mann-Whitney *U* test (ST59 versus ST239). (**B**) Distribution of virulence genes across 10 functional categories in the two lineages. Two-sided Mann-Whitney *U* test (ST59 versus ST239). (**C** and **D**) Correlation between the number of virulence genes and phylogenetic distance from the root within ST239 (*n* = 184) and ST59 (*n* = 401) populations. Each point represents an individual strain. Linear regression lines and 95% confidence intervals are shown; *R*^2^ and *P* values are based on linear regression. (**E**) Association between the presence of specific virulence genes and cytolytic activity (measured by THP-1 lysis) among ST59 (*n* = 401) and ST239 (*n* = 184) isolates. In each panel, MRSA lineages are grouped on the *x* axis by virulence-gene status (present versus absent); the *y* axis shows THP-1 cell lysis (%). Two-sided Mann-Whitney *U* test (absence versus presence). (**F**) Structural domains of the SraP protein carried in most ST59 genomes. (**G**) Comparison of SraP domain structures among genomes from ST59, ST239, and other STs. (**H**) Structural variation in the *sraP* gene locus between ST59 and ST239. Arrows indicate genes; color shading between genomes denotes sequence homology, with darker shading representing higher similarity. ***P < 0.001 and ****P < 0.0001; ns, no significant difference. aa, amino acid.

We first compared the distribution of virulence-associated genes between ST239 and ST59 across various functional categories and examined their temporal dynamics over a 10-year period. Unexpectedly, ST239 isolates presented a significantly greater abundance of virulence genes than ST59 (Fig. 5A, *P* < 0.001). Specifically, ST239 harbored a greater number of virulence genes across multiple functional categories, with the exception of those associated with adhesion, enterotoxin production, iron uptake, and secretion system functions (Fig. 5B). Moreover, the number of virulence genes in the ST239 strains isolated from 2011 to 2020 remained relatively stable (Fig. 5D, *P >* 0.05). These findings suggest that the overall abundance of virulence genes does not determine a strain’s pathogenic potential. Rather, it is the presence or absence of specific key virulence determinants, or functional variations within them, that plays a critical role in virulence. Thus, we performed a genome-wide association study (GWAS), integrating genomic data on the distribution of known virulence genes with phenotypic measurements of cell lysis. Using a presence-absence matrix of known virulence genes and linear regression models adjusted for population structure, we identified six genes (*lukS-PV*, *lukF-PV*, *seb*, *selk*, *selq*, and *chp*) that showed the strongest statistical associations with increased cytotoxicity (adjusted *P* < 0.01; Fig. 5E and fig. S12).

We first examined *lukS-PV*/*lukF-PV*, which encodes PVL, one of the loci associated with inducible cytotoxicity. This locus is present on the PVL phage, a genetic element that is found almost exclusively in CA-MRSA and has been previously reported to be lost in some bloodstream isolates (*16*–*18*). However, in this study, the prevalence of the *lukS*/*lukF-PV* encoding the PVL cytotoxin in ST59 was only 33.17% (fig. S6). Although *seb*, *selk*, and *selq* were enriched among high-cytotoxicity strains, we did not prioritize them for further analysis in this study because their functional redundancy and overlapping roles as enterotoxins complicate the attribution of cytotoxic effects to any individual gene in the absence of targeted functional validation.

The second locus of interest was *chp*, which is consistently present in ST59 clones but absent in ST239 (Fig. 5E and fig. S6). *chp* encodes CHIPS (chemotaxis inhibitory protein of *S. aureus*), which specifically inhibits the response of neutrophils and monocytes to C5a and fMLP (*19*, *20*). Our previous research suggested a potential role for the *chp* gene in driving the dominance of MRSA ST59 lineage (*21*). In addition, the outcomes of the GWAS reaffirmed the critical role of the *chp* gene.

However, our GWAS detected only genes associated with differences within lineages after population structure correction and, by design, did not identify genes that explain differences in cytotoxicity between ST239 and ST59 when determinants are fixed between lineages. Accordingly, between lineage differences were defined by comparative genomics. Among shared virulence genes with sequence variation, *sraP* showed one of the most notable differences, which encodes a serine-rich surface glycoprotein implicated in bacterial adhesion and host interaction. Detailed sequence comparisons revealed that while most ST59 isolates harbored a full-length *sraP* gene, all ST239 strains encoded truncated versions with large deletions primarily within the serine-rich SRR2 region (Fig. 5, F and G). Genomic context of *sraP* across ST59, ST239, and reference strains (N315, USA300, and NCTC 8325) were further compared. While the surrounding genomic region was highly conserved, all sequence deletions distinguishing ST239 from ST59 localized to the SRR2 region of *sraP* (Fig. 5H). Despite speculation that SRRPs reside within genomic islands, no evidence of horizontal gene transfer was detected near *sraP* (Fig. 5H).

Notably, as shown in fig. S1A, clade I, the most recently emerged (~1995.7) in ST59 lineage, exhibited a large segmental deletion within the SRR2 domain of *sraP*. In contrast, *sraP* remained intact in other ST59 clades, suggesting divergent evolutionary paths.

### *chp* enhances immune evasion capacity, whereas *sraP* facilitates invasion and adhesion in ST59

**Fig. 6.**
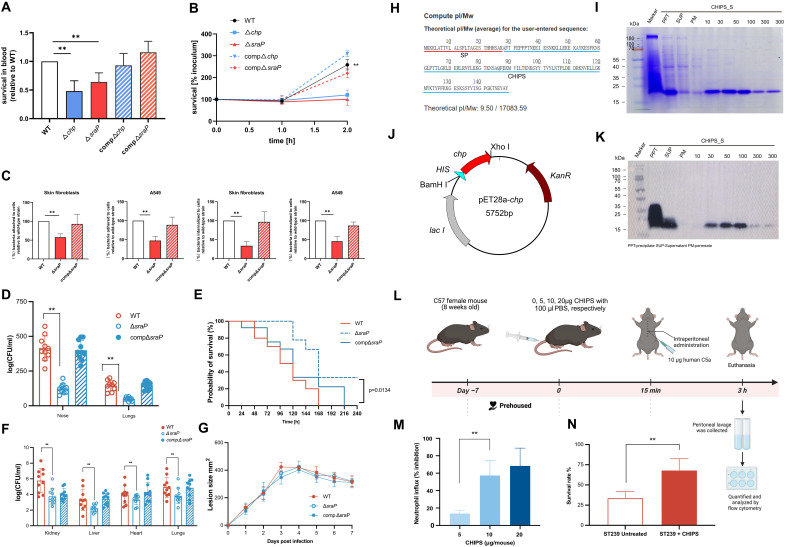
*chp* enhances immune evasion capacity, whereas *sraP* facilitates invasion and adhesion in ST59. (**A**) Survival of the wild-type (WT), Δ*chp*, Δ*sraP*, and complemented strains in human whole blood, normalized to WT = 1.0. (**B**) Serum survival of WT, Δ*chp*, Δ*sraP*, and complemented strains after 2-hour incubation. (**C**) Adhesion and invasion of WT, Δ*sraP*, and complemented strains in human dermal fibroblasts and A549 epithelial cells. (**D**) Bacterial loads in the noses and lungs of mice infected with WT, Δ*sraP*, and complemented strains. (**E**) Survival of mice intravenously challenged with ST59 or ST239 over 10 days (*n* = 20 per group); survival curves compared by log-rank test. (**F**) Bacterial burdens in the kidney, lungs, liver, and heart of mice infected with WT, Δ*sraP*, and complemented strains. (**G**) Skin abscess areas following subcutaneous infection with WT, Δ*sraP*, and complemented strains. (**H**) Predicted CHIPS protein encoded by chp, with a molecular weight of ~17 kDa and isoelectric point of 9.50; red underline denotes the signal peptide and blue underline the mature CHIPS protein. (**I**) Schematic diagram of pET28a-CHIPS plasmid construction. (**J**) Purification of recombinant CHIPS (rCHIPS) shown by 15% SDS-PAGE with Coomassie staining: Lane 1, protein marker; lane 2, pellet; lane 3, supernatant; lane 4, flow-through; lanes 5 and 6, imidazole elutions. (**K**) Western blot analysis of purified recombinant CHIPS using an anti-His tag monoclonal antibody, with the same lane order as described in (J). (**L**) Schematic of the mouse model intraperitoneal challenge with CHIPS. Created in BioRender. Y. Jin (2026) https://BioRender.com/xnr2qdb. (**M**) C5a-induced neutrophil migration is inhibited by CHIPS. (**N**) Survival of ST239 in human whole blood with or without exogenous CHIPS treatment. Two-sided Mann-Whitney *U* tests were used for pairwise group comparisons. Data are presented as mean ± SD. **P < 0.01.

Therefore, we identified *chp* and *sraP* as key virulence determinants and constructed isogenic deletion mutants in a representative ST59 isolate SKLX15777 (SCC*mec* IVa; *spa* type t437). We subsequently applied diverse infection models to dissect their contributions to survival, immune evasion, colonization, and pathogenicity. Whole-blood survival assays revealed notably reduced survival of both mutants compared to the wild-type (WT) and complemented strains (Fig. 6A). Furthermore, coculture with purified human neutrophils demonstrated distinct survival kinetics: While all strains exhibited comparable survival during the first hour, only the WT and complemented strains subsequently proliferated, indicating effective evasion of neutrophil-mediated killing (Fig. 6B). In contrast, the Δ*chp* and Δ*sraP* mutants failed to expand, showing limited survival even after 2 hours. These findings suggest that *chp* and *sraP* contribute to early immune evasion by enabling resistance to neutrophil clearance, rather than directly promoting bacterial replication. Their absence compromises the ability of ST59 to persist under innate immune pressure.

*sraP* encodes a serine-rich surface adhesin implicated in epithelial attachment and immune modulation (*22*, *23*), we hypothesized that it also contributes to host colonization and tissue invasion. Compared with the WT strain, the Δ*sraP* mutant exhibited reductions of approximately 43.3% in adhesion and approximately 52.8% in invasion upon incubation with human dermal fibroblasts (Fig. 6C). Similar trends were observed in A549 alveolar epithelial cells, where the Δ*sraP* mutant displayed reductions of approximately 66.2% in adhesion and approximately 54.4% in invasion (Fig. 6C). These results collectively indicate that *sraP* plays a critical role in mediating ST59 adhesion to and invasion of epithelial cells, supporting its importance in early host colonization.

To further evaluate its role in vivo, we next assessed whether *sraP* contributes to this process in vivo*.* Mice intranasally inoculated with the Δ*sraP* mutant exhibited a markedly lower bacterial burden in the nasal cavity compared with those infected with the WT or complemented strains (Fig. 6D). These findings indicate that *sraP* is important for efficient colonization of the nasal mucosa, suggesting that the acquisition or maintenance of this gene may enhance the capacity of ST59 to persist in the host and facilitate transmission.

Given our data showing that ST59 exhibited greater lethality than ST239 in the murine BSI models (Fig. 4F), we next sought to determine whether this heightened virulence could be attributed, at least in part, to the presence of the *sraP* gene. To this end, we evaluated the role of *sraP* using a high-dose systemic infection model. Mice infected with the WT ST59 strain harboring an intact *sraP* gene all succumbed to infection within 7 days, whereas those infected with the isogenic Δ*sraP* mutant exhibited a strikingly improved survival rate of over 66% (Fig. 6E). To further investigate the impact of *sraP* on in vivo bacterial dissemination, we performed a low-dose infection experiment and quantified bacterial burdens in major organs at 48 hours postinfection. Mice infected with WT or complemented ST59 strains exhibited significantly higher bacterial loads in the kidneys, liver, and spleen compared to those infected with either the Δ*sraP* mutant or ST239 strains (Fig. 6F), suggesting that *sraP* contributes to enhanced survival and systemic spread within the host.

Given the pronounced tissue-destructive phenotype of ST59 observed in the subcutaneous infection model, we next sought to determine whether *sraP* contributes to this enhanced local virulence. However, when comparing the WT ST59 strain with its isogenic Δ*sraP* and complemented strains in the murine subcutaneous infection model, we observed no marked differences in abscess size or local pathology (Fig. 6G), indicating that *sraP* does not contribute measurably to virulence in this context. This suggests that the pronounced tissue damage mediated by ST59 is likely independent of *sraP* and may instead involve other virulence determinants.

We next turned our attention to *chp*, which encodes CHIPS, a protein known to inhibit neutrophil chemotaxis by blocking the C5a receptor (*24*). We hypothesized that CHIPS-mediated immune evasion may contribute to the enhanced tissue persistence and inflammatory pathology observed in ST59 infections. To test this, we generated the purified recombinant CHIPS protein (Fig. 6, H to K). To investigate the specific inhibitory effect of CHIPS on neutrophil recruitment in vivo, we used a well-established mouse model of human C5a-induced peritoneal inflammation (Fig. 6L). As shown in Fig. 6M, administration of at least 10 μg of CHIPS resulted in a considerable inhibition (~55%) of neutrophil influx compared with phosphate-buffered saline (PBS)–treated controls, confirming the in vivo functionality of CHIPS as a potent antagonist of the human C5a receptor.

To assess the immunoevasive function of CHIPS in the context of innate immune pressure, we evaluated whether exogenous CHIPS supplementation could enhance bacterial survival in human whole blood. Pretreatment of CHIPS-negative ST239 strains with recombinant CHIPS conferred a modest but statistically significant increase in survival compared to untreated controls (Fig. 6N), indicating that CHIPS can mitigate host-mediated killing in the early stages of infection. These results reinforce the role of CHIPS as a key immune evasion factor that inhibits C5a-mediated neutrophil responses.

### Superior acid tolerance drives the expansion of ST59

Virulence alone does not fully account for increasing prevalence of ST59. The ability of strains to persist and disseminate in diverse host and environmental niches also relies heavily on its capacity to withstand a variety of stresses. Therefore, to further characterize the physiological differences between the ST59 and ST239 that may contribute to their differential fitness and persistence in clinical settings, we next evaluated the ability of 20 representative isolates from each lineage to survive under a range of environmental stress conditions.

**Fig. 7.**
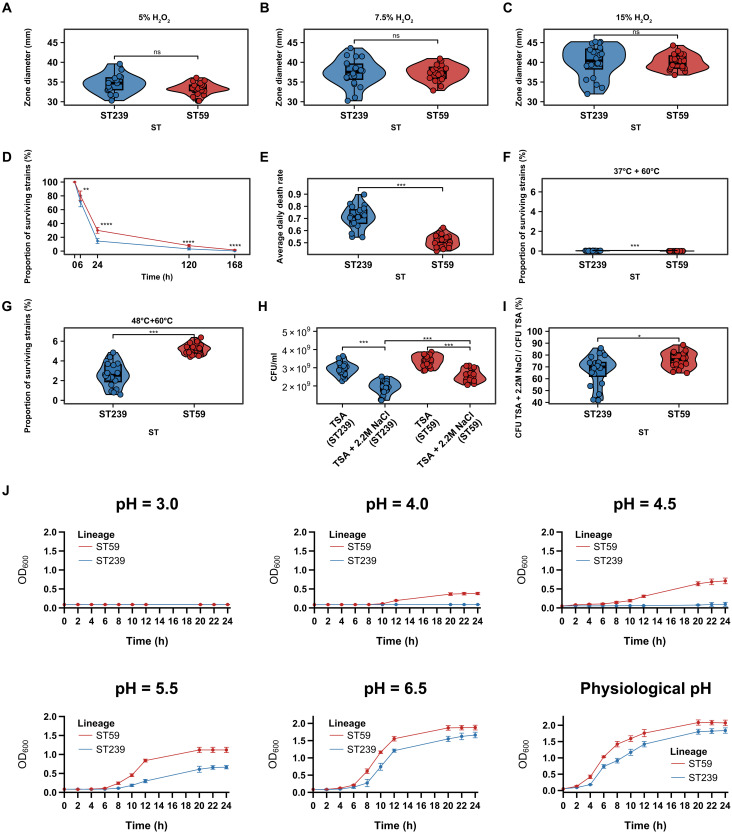
Comparison of resistance to environment-related stress between ST239 (*n* = 20) and ST59 (*n* = 20). (**A** to **C**) The distribution of inhibition zone diameters for ST239 and ST59 strains under different concentrations of 5 (A), 7.5 (B), and 15% (C) H_2_O_2_, respectively. A larger inhibition zone diameter indicates a weaker tolerance to hydrogen peroxide. (**D**) The survival rates (mean ± SDs) of ST239 and ST59 strains under identical desiccation conditions at different time points. (**E**) The distribution of average daily mortality rates of ST239 and ST59 strains at 120 hours. (**F** and **G**) The survival rates of ST239 and ST59 lineages following preincubation at physiological temperature (37°C; F) and sublethal temperature (48°C; G), when subsequently exposed to a lethal temperature (60°C). The survival rates of all tested strains were determined by CFU enumeration. (**H**) The distribution of CFU counts for ST239 and ST59 strains under different osmotic conditions, including physiological osmotic conditions (TSA) and high osmotic stress (TSA supplemented with 2.2 M NaCl). (**I**) The distribution of the ratios of CFU counts under high osmotic stress to those under physiological conditions for ST239 and ST59 strains. (**J**) Growth curves of ST239 (blue) and ST59 (red) were measured in TSB adjusted to pH 3.0, 4.0, 4.5, 5.5, and 6.5, respectively. TSB with a physiological pH of 7.4 served as the control condition. Two-sided Mann-Whitney *U* test (ST59 versus ST239). *P < 0.05, **P < 0.01, ***P < 0.001, and ****P < 0.0001; ns, no significant difference.

We first established a baseline by assessing oxidative stress tolerance under various concentrations of hydrogen peroxide. As show in Fig. 7 (A to C), ST59 and ST239 exhibited comparable resistance to oxidative stress across all tested H_2_O_2_ concentrations. Under desiccation conditions, ST59 exhibited significantly higher survival rates than ST239 at 6, 24, 120, and 168 hours (*P* < 0.01; Fig. 7D), consistent with a markedly lower average daily death rate (mean: 0.51 versus 0.71, *P* < 0.0001; Fig. 7E), suggesting enhanced persistence in dry environments such as on fomites or skin surfaces.

Thermotolerance assays revealed that both ST59 and ST239 strains were susceptible to acute heat stress at 60°C, with nearly all cells rendered nonviable after direct exposure (Fig. 7F). However, when preconditioned at a sublethal temperature of 48°C, ST59 exhibited significantly greater survival following lethal heat exposure than ST239 (mean: 4.95% versus 2.71%, *P* < 0.0001; Fig. 7G), indicating an enhanced capacity for heat adaptation. In hyperosmotic conditions, both lineages displayed reduced viability (*P* < 0.001; Fig. 7H), yet ST59 maintained significantly higher survival than ST239 (*P* < 0.05; Fig. 7I), reflecting greater osmotolerance.

Most notably, considerable differences in acid resistance were observed between the two lineages. Under neutral conditions (pH 7.4), ST59 displayed a slightly higher growth rate than ST239 (Fig. 7J), serving as a physiological baseline. While neither lineage was capable of growth at pH 3.0 (Fig. 7J), ST59 sustained slow but detectable growth at pH 4.0, whereas ST239 failed to grow under these conditions. Moreover, ST59 exhibited clearly enhanced survival across a range of acidic pH values (4.5, 5.5, and 6.5) compared to ST239 suggesting robust acid tolerance that may be advantageous in acidic host microenvironments such as the skin surface, abscesses, or phagolysosomes. In our data, acid tolerance (measured as the maximum growth rate at pH 4.5) correlated positively with skin lesion size in ST59 (*P* < 0.01) (fig. S11A).

To explore the underlying mechanisms contributing to the superior acid tolerance of ST59, we conducted transcriptomic profiling of a representative ST59 isolate SKLX15777 (SCC*mec* IVa; *spa* type t437) during exponential growth at acidic (pH 4.5) versus physiological (pH 7.4) conditions. Differential expression analysis revealed considerable changes in 312 genes (fig. S13A). Among them, a marked down-regulation of virulence-associated genes was observed (fig. S13B). Of particular note, 35 previously reported acid-responsive genes showed altered expression, including genes involved in urease activity (*ure* operon), nucleotide biosynthesis, and ribosomal function. Approximately 40% (14 of 35) of these were transport-related genes, such as the hemin efflux pumps (*hrtAB*) and putative drug efflux pumps (*emrAB*), both of which were up-regulated by over 87-fold under acidic stress.

Gene ontology (GO) and Kyoto Encyclopedia of Genes and Genomes (KEGG) pathway enrichment analyses indicated that protein synthesis and metabolic pathways were notably enhanced under acidic conditions (fig. S13, C to E). Membrane transport–related genes ranked among the top three most enriched functional categories, alongside those associated with translation and amino acid metabolism (fig. S13F). These transcriptomic shifts may collectively underpin the physiological resilience of ST59 in acidic environments.

## DISCUSSION

Despite the considerable clinical burden posed by MRSA, large-scale, long-term genomic epidemiological studies of BSI-causing MRSA strains are still limited. To our knowledge, this study constitutes the most extensive and longest-running investigation of its kind to date. MRSA ST239, one of the most prominent historical clones, has long been associated with hospital-acquired infections and high levels of multidrug resistance across Asia (*11*, *25*–*27*). Harris *et al.* (*11*) have traced the global spread and hospital adaptation of ST239, confirming that clinical practice (specifically the use of currently deployed antibiotics) is a major driver of pathogen evolution. In this study, we found that the past decade has witnessed a striking clonal replacement event among BSI isolates in which the historically dominant ST239 lineage was supplanted by ST59 in China. This clonal replacement appears to be rooted in fundamental differences in the evolutionary origin and genome architecture of the two lineages. The hybrid origin of ST239 has been established by earlier studies (*9*, *10*). Here, we confirm this chimeric architecture in a large, China-wide BSI cohort (2011–2020) and use it as the lineage framework for comparisons with ST59. Genomic chimerism in ST239 may entail long-term fitness costs (*10*). In contrast, ST59 evolved along a recombination-sparse path, maintaining core-genome stability and avoiding burdens linked to extensive interlineage recombination. Further, our within-lineage analyses support a greater contribution of recombination to genetic diversification in ST239 than in ST59 during the surveillance period. These contrasting evolutionary modes are further reflected in MGE dynamics: ST239 underwent genome reduction characterized by the loss of large MGEs and functional attrition, whereas ST59 retained a reduced yet functionally potent MGE repertoire enriched for immune evasion genes such as *chp*, *seb*, and *sey*. Given that the maintenance and transfer of MGEs impose resource costs on the bacterial host (*28*–*31*), such remodeling in ST59 may have enhanced its epidemiological fitness among BSI isolates by minimizing genetic burden while retaining essential virulence-associated traits.

The decline of ST239 and the concurrent rise of ST59 represent a pivotal evolutionary shift in the BSI MRSA landscape of China. To explore the factors underlying the progressive decline of the ST239 lineage, we assessed their AMR profiles and historical selective pressures. In our dataset, ST239 strains carried a substantially higher AMR burden than ST59, consistent with the notion that multidrug resistance has historically conferred a critical selective advantage for MRSA clones in health care settings under intense antibiotic pressure (*32*, *33*). During the early 2000s, the global expansion of ST239 was closely linked to its ability to withstand commonly used antibiotics such as β-lactams, fluoroquinolones, and aminoglycosides, which were frequently deployed in hospital environments at the time (*4*, *34*, *35*). This resistance repertoire allowed ST239 to outcompete more susceptible lineages and establish dominance across Asia and other regions with high antimicrobial usage (*25*). Supporting this trend, a recent 12-year genomic analysis of a hospital ST239 population demonstrated that adaptive changes in MRSA were closely linked to patterns of vancomycin consumption, with higher usage selecting for strains with reduced glycopeptide susceptibility (*36*). However, with the implementation of antimicrobial stewardship programs and the subsequent reduction in the use of antibiotics such as fluoroquinolones and aminoglycosides in China (*37*), the multidrug-resistant profile of ST239, once a key advantage, has increasingly become a liability. In line with these observations, data from the Center for Antibacterial Surveillance in China showed that inpatient antimicrobial consumption declined from a median of 65.00 DDDs/100 bed-days in 2011 to 44.12 in 2020, inversely correlating with ST239 prevalence (*R*^2^ = 0.85 and *P* < 0.001). Notably, resistance classes where ST239 had a clear advantage, such as aminoglycosides and fluoroquinolones, also declined over the same period (aminoglycosides: from 3.14 to 1.10 DDDs/100 bed-days, *R*^2^ = 0.85 and *P* < 0.001; fluoroquinolones: from 8.64 to 6.22 DDDs/100 bed-days, *R*^2^ = 0.48 and *P* < 0.05), further eroding its selective benefit. Collectively, these trends indicate that decreasing antimicrobial pressure has facilitated the emergence and dominance of less resistance-burdened clones like ST59.

Moreover, this resistance-driven advantage came at a cost (*38*, *39*). As ST239 progressively accumulated resistance determinants, it exhibited substantially slower growth and reduced competitive fitness in vitro, suggesting that the maintenance of extensive resistance mechanisms imposed a substantial metabolic burden. Concurrently, the ST239 genome showed clear signs of functional attrition, with increased pseudogene content and reduced metabolic flexibility, further compromising its epidemiological fitness and capacity for long-term persistence. In addition, while ST239 showed a higher overall gene content diversity, it appears to be approaching an evolutionary dead end. In contrast, ST59 maintained an open pan-genome architecture, indicating sustained genomic plasticity and a greater evolutionary capacity to respond to future selective pressures (*40*).

While ST239 has waned, ST59 has expanded to dominance among BSI isolates, potentially driven in part by its unique virulence advantages. In our study, cytotoxicity assays demonstrated that ST59 isolates caused greater host cell lysis than ST239. Consistent with previous comparative study, ST239 exhibits reduced cytotoxicity relative to other lineages (*41*, *42*). This enhanced cytotoxic phenotype of ST59 was also found in vivo, manifesting as more severe skin lesions, higher systemic bacterial burdens, and increased mortality. Collectively, these findings suggest that the elevated cytotoxic potential of ST59 may promote tissue invasion, immune evasion, and persistence within the host, thereby conferring a selective advantage during clonal competition. Previous studies have demonstrated that strains with elevated cytotoxic capacity are more likely to induce severe clinical manifestations and exhibit enhanced systemic dissemination, particularly in immunocompetent hosts (*43*, *44*). Such strains can induce extensive tissue pathology and modulating host immune defenses in a manner that amplifies disease severity while simultaneously promoting intrahost survival and efficient transmission between hosts (*8*, *45*, *46*). These virulence-associated traits have been increasingly recognized as critical determinants of clonal success (*47*–*50*), providing a competitive advantage in both host colonization and interhost spread, and ultimately promoting the epidemiological dominance of hypervirulent lineages (*5*).

Despite carrying a broader repertoire of virulence-associated genes, ST239 strains exhibited markedly lower in vivo pathogenicity compared to ST59. This apparent paradox underscores an important concept: the overall number of virulence genes may be less critical than the presence of specific, intact factors. To elucidate the genetic basis underlying ST59’s enhanced pathogenicity, we performed genome-wide association analyses integrating phenotypic cytotoxicity data with genomic profiles. Six candidate genes (*lukS-PV*, *lukF-PV*, *seb*, *selk*, *selq*, and *chp*) were identified substantially associated with elevated cytotoxicity. Meanwhile, sequence variation analysis of shared virulence genes between the two lineages highlights the *sraP* gene as a primary target. Notably, *chp* and intact *sraP* stood out due to their lineage-specific presence in ST59 and their established roles in immune evasion (*51*, *52*) and host adhesion (*53*, *54*), respectively.

The *chp* encodes the CHIPS, which can specifically inhibit the responses of neutrophils and monocytes to C5a and fMLP (*19*, *20*). When *S. aureus* invades the human host, the complement system is rapidly activated, leading to opsonization of bacteria and the generation of a notable amount of C5a (*55*). The production of C5a, along with bacterial translation by-products, collectively constitutes the first trigger of the innate immune system (*56*). The host’s innate immune system recognizes early signs of bacterial invasion through two related receptors on neutrophils, C5aR (the complement receptor on neutrophils) and FPR-1 (formyl peptide receptor-1), and CHIPS can inhibit the activation of immune cells by binding to both receptors, thus buying time for MRSA to establish a rapid infection (*57*). In our study, deletion of *chp* and *sraP* led to substantially reduced bacterial survival in whole blood and during neutrophil coculture, underscoring its role in evading early innate immune responses, providing a window of opportunity for infection and proliferation. Moreover, administration of recombinant CHIPS protein suppressed neutrophil influx in vivo and enhanced bacterial survival ex vivo, reinforcing its functionality as a key immune evasion factor. These results are consistent with prior findings that chemotaxis interference contributes to immune escape and increased fitness during host colonization and systemic spread (*58*). However, CHIPS plays a vital role only in the early stages of bacterial invasion (*51*, *52*). Once the strains proliferate extensively, the substantial release of other toxins weakens the inhibitory effect of CHIPS, leading to a marked influx of neutrophils into the infection site and triggering a severe inflammatory response (*59*).

The *sraP* gene, encodes a serine-rich surface adhesin implicated in epithelial adhesion and invasion. In this study, we confirmed that *sraP* deletion markedly reduced bacterial adhesion to and invasion of both dermal and alveolar epithelial cells. Furthermore, *sraP* was critical for nasal colonization and systemic dissemination, as evidenced by diminished bacterial burdens in the nasal mucosa and major organs following infection with the Δ*sraP* mutant. In addition, *sraP* deletion led to substantially improved host survival in systemic infection models, establishing a clear link between *sraP* and increased virulence. *sraP* did not contribute measurably to local tissue damage in skin abscess models, suggesting that ST59’s subcutaneous virulence is driven by other determinants, such as cytolytic toxins.

To survive the harsh conditions commonly found in health care environments, such as exposure to disinfectants, high osmolarity, oxidative stress, and pH fluctuations, succeed clones must have robust physiological adaptability. Our comparative phenotypic analyses revealed that while ST59 and ST239 displayed similar tolerance to oxidative stress, ST59 demonstrated markedly enhanced survival under desiccation, heat, high osmolarity, and particularly acidic conditions (pH 4.0 to 6.5). This trait is especially advantageous, as acidic microenvironments, such as those on skin surfaces, within abscesses, or inside phagolysosomes, are increasingly recognized as key sites for early host-pathogen interactions and persistent colonization (*60*, *61*). Similar patterns have been observed in other globally successful lineages. For instance, the livestock-associated clone ST398 has demonstrated enhanced survival under dry conditions and oxidative stress, which may facilitate its persistence in both human and animal environments (*62*). Likewise, the epidemic clone USA300 shows high-level resistance to acidic pH and reactive oxygen species (*63*). The hospital-associated ST22 lineage (EMRSA-15) has also evolved metabolic flexibility and stress resistance traits that may promote its success in nosocomial settings (*64*).

The specific acid response mechanisms behind enhanced acid tolerance in ST59 remain poorly understood. In this study, we found that exposure to acidic pH triggered a broad and coordinated adaptive response, characterized by the up-regulation of genes involved in membrane transport, ribosomal function, and central metabolic pathways. Notably, genes encoding efflux pumps such as *hrtAB* and *emrAB*, which are involved in heme detoxification (*65*, *66*) and multidrug resistance, respectively, were substantially up-regulated. These transporters likely contribute to acid resistance by exporting toxic metabolic by-products or maintaining intracellular ion homeostasis, thereby mitigating acid-induced cellular damage (*67*, *68*). In addition, the urease gene cluster (*ureABC*) and associated accessory genes (*ureEFGD*), which catalyze the hydrolysis of urea to produce ammonia, were also up-regulated. This enzymatic activity can locally buffer acidic pH by generating alkaline compounds (*69*), providing a mechanism for *S. aureus* to survive in hostile niches such as abscesses and phagolysosomes (*70*, *71*). The up-regulation of these systems aligns with previous findings showing that acid stress triggers the activation of proton extrusion, ammonia production, and membrane repair pathways to restore pH balance and protect cellular integrity (*58*, *72*). The observed down-regulation of virulence-associated genes under acid stress is also consistent with previously reported trade-offs between pathogenesis and stress tolerance (*73*), highlighting the lineage’s ability to modulate gene expression for survival rather than overt virulence under hostile conditions.

Moreover, ST59 exhibited increased expression of ribosomal proteins and genes involved in amino acid metabolism and ATP biosynthesis under acid stress, indicating an active effort to maintain protein synthesis and energy homeostasis even in hostile environments. This stands in contrast to the translational shutdown observed in more acid-sensitive lineages, suggesting that ST59 avoids stress-induced metabolic arrest and instead preserves its growth and virulence potential under low-pH conditions. Similar transcriptional resilience under acid stress has been reported in other pathogenic bacteria, such as *Listeria monocytogenes*, where sustained energy production and protein synthesis contribute to intracellular survival and pathogenesis (*74*). Together, these findings reinforce the view that stress tolerance, particularly acid resistance, acts as a critical evolutionary force and, together with virulence and reduced MGE burden, collectively contributed to the epidemiological success of MRSA ST59 among BSI isolates.

While our study provides a comprehensive, multilayered investigation into the evolutionary dynamics of MRSA clonal replacement in China, several limitations should be acknowledged. First, our analysis was restricted to bloodstream isolates, which, although clinically important, may not reflect the broader ecology and transmission of *S. aureus* across colonizing, noninvasive, or environmental reservoirs that experience different selective pressures; extrapolation beyond invasive disease should therefore be made with caution. Second, while we used an integrative framework combining genomic, transcriptomic, and functional assays, including gene knockouts, recombinant protein experiments, and murine models, to identify and validate key virulence determinants, other host-specific factors and environmental variables (e.g., host immunity, antibiotic exposure at the individual level, and hospital hygiene practices) were not systematically incorporated. Third, our functional characterization focused primarily on two lineage-specific effectors, *chp* and *sraP*; while their synergistic contributions to immune evasion and colonization were experimentally validated, additional genetic or epigenetic factors contributing to the observed phenotypes may remain uncharacterized. Last, although our genomic dataset spans 72 hospitals across China over a 10-year period, the generalizability of our findings to other geographical regions or MRSA clones warrants further validation through international surveillance and comparative studies. With collection restricted to 2011–2020 and few early ST239 genomes (ST239-I) in the cohort, we cannot test whether ST239 became progressively more degraded or whether ST59 became progressively more virulent over time. Establishing temporal causality will require broader historical sampling, including pre-2010 genomes and non-BSI reservoirs. In addition, given that any *S. aureus* could serve as a donor, future work should rigorously compare recombination rates between ST59 and ST239 by situating our isolates within a large, globally sourced *S. aureus* collection using tools such as fastGEAR (*75*).

In conclusion, our nationwide genomic analysis reveals that the clonal replacement of ST239 by ST59 among BSI isolates in China was not simply a matter of antibiotic resistance erosion, but the result of a multifaceted evolutionary advantage. ST59’s dominance was shaped by the combined action of a reduced MGE burden, optimized virulence, lineage-specific virulence determinants, and superior stress tolerance, particularly acid resistance. These traits collectively enhanced its capacity for immune evasion, tissue invasion, and survival in hostile host niches. Our identification of *chp* and s*raP* as key determinants underscores the role of virulence traits in clonal success. Our findings advance the current understanding of MRSA clonal dynamics and underscore the need for surveillance frameworks that incorporate epidemiological fitness and evolutionary adaptability, rather than relying solely on AMR profiling and the presence of individual virulence determinants.

## MATERIALS AND METHODS

### Study design and collection of *S. aureus* strains

This laboratory-based, multicenter retrospective study was conducted from January 2011 to December 2020 as part of the national BSI surveillance program [Blood Bacterial Resistant Investigation Collaborative System (BRICS)]. A total of 3848 nonduplicate *S. aureus* isolates were collected from 72 sentinel hospitals across 22 provinces in China and identified via matrix-assisted laser desorption/ionization–time-of flight mass spectrometry. Isolates were voluntarily submitted by participating hospitals; therefore, the dataset does not capture all BSI cases during the study period. Only nonduplicate, clinically confirmed *S. aureus* isolates from the first BSI episode per patient with complete metadata (isolation date, hospital source, and ward type) were included; contaminants, mixed cultures, or isolates lacking essential data were excluded. Isolates with an oxacillin MIC ≥4 mg/liter were classified as MRSA according to the Clinical and Laboratory Standards Institute (CLSI) criteria (CLSI M100, 30th edition), yielding 1244 MRSA isolates for further analysis (table S1). Antimicrobial susceptibility testing was performed using agar dilution (for 12 agents including oxacillin, penicillin, erythromycin, clindamycin, trimethoprim-sulfamethoxazole, tetracycline, ciprofloxacin, levofloxacin, moxifloxacin, gentamicin, amikacin, and rifampicin) and broth microdilution (for tigecycline, linezolid, daptomycin, and vancomycin), following CLSI guidelines. *S. aureus* ATCC 25923 and 29213 served as quality control strains. Details of the strains selected for comparative fitness experiments, animal infection, genetic manipulation, and other phenotypic assays are provided in table S3; strains were selected by clade-stratified random sampling within the ST59 and ST239 lineages. This study complies with all relevant ethical regulations and was approved by the Ethics Committee of the First Affiliated Hospital of Zhejiang University (approval number: IIT20210311B). All animal experiments were conducted following the guidelines of the Institutional Animal Care and Ethics Committee at the First Affiliated Hospital of Zhejiang University (approval number: 2021-929).

### WGS and genomic analysis

Genomic DNA of the 1244 MRSA isolates was extracted using the Ezup Column Bacteria Genomic DNA Purification Kit (Sangon Biotech, Shanghai, China). The MRSA genomes were sequenced on a HiSeq X Ten platform (Illumina, San Diego, CA, USA) with a 2 × 150 bp read length. The processed reads, after adaptor trimming and quality filtering by fastp version 0.20.1 (*76*), were assembled using SPAdes version 3.13.0 (*77*). MLST, SCC*mec* typing, and *spa* typing of the MRSA strains were conducted using web-based tools MLST (https://cge.food.dtu.dk/services/MLST), SCCmecFinder (https://cge.food.dtu.dk/services/SCCmecFinder), and SpaFinder (https://cge.food.dtu.dk/services/spaTyper), respectively. Genomes were annotated using dfast version 1.2.18 (*78*). The pseudogenes in genomes were identified by dfast version 1.2.18 (*78*) and pseudofinder version 1.1.0 (*79*). Virulence and AMR genes were identified using ABRicate version 1.0.0 (BLASTn-based; https://github.com/tseemann/abricate) against VFDB setB (accessed in July 2023) and ResFinder version 2.1.1, using thresholds of ≥85% identity and ≥85% query coverage.

### Phylogenetic analysis of ST59 and ST239

Trimmed sequencing reads of all ST59 (*n* = 401) and ST239 (*n* = 184) in this study were mapped to their respective reference genomes (M013 for ST59, accession no. CP003166.2; TW20 for ST239, accession no. FN433596.1) using Snippy version 4.6.0 (https://github.com/tseemann/snippy) to generate core genome alignments. Recombination regions within the core genomes were detected and subsequently removed before further phylogenetic analysis using Gubbins version 2.4.1 (*80*). Maximum likelihood phylogenetic trees for ST59 and ST239 were constructed in RAxML version 8.2.12 (*81*) under the GTR + GAMMA model (four discrete rate categories), using rapid bootstrapping combined with the best-scoring ML search (-f a) and 1000 bootstrap replicates; all other settings were left at default values. Genomes of MRSA MW2 (ST1, accession no. NC_003923.1) and N315 (ST5, accession no. NC_002745.2) were incorporated as outgroups for the phylogenetic analyses of ST59 and ST239, respectively. Temporal phylogenetics for the ST59 and ST239 were estimated using the BactDating version 1.1 (*82*), with mixedcarc model and 200 million Markov chain Monte Carlo (MCMC) iterations. Convergence of MCMC was confirmed with effective sample sizes greater than 200 for all model parameters. The resulting phylogenetic trees, along with the metadata, were visualized using ggtree version 3.7.1.003.

### Detection of genes under positive selection

The detection of genes under positive selection in ST59 and ST239 was performed as previously described (*83*). In brief, ancestral states of all core single-nucleotide polymorphisms (SNPs) were initially reconstructed using PAML 4 (*84*). Mutations were categorized as intergenic, synonymous, or nonsynonymous based on comparison of each SNP with its annotations and reconstructed ancestral states. The dN/dS rates were adjusted according to transition/transversion rates and codon frequencies under the NY98 model. Genes with an adjusted dN/dS ratio >1.5 in ST239 or >2 in ST59 were under positive selection. In addition, genes with homoplastic SNPs (*85*) (evolved independently at least three times) were also considered to be under positive selection.

### Genetic origin analysis of ST239 and ST59 MRSA

All available *S. aureus* genome sequences, totaling 32,424 as of July 2022, were downloaded from the NCBI GenBank database. Each strain was classified by ST type and annotated. For ST239, we used Assembly Dereplicator version 0.3.1 (https://github.com/rrwick/Assembly-Dereplicator) to cluster and dereplicate all ST8 and ST30 genomes, selecting 66 ST8 and 72 ST30 genomes for further analysis. Core genes from all ST239 and the selected ST8 and ST30 genomes were extracted using Panaroo version 1.3.0 (*86*). These genes were then aligned by ST with mafft version 7.508 (*87*). Consensus sequences for each ST’s core genes were obtained using the EMBOSS toolkit (*88*), and their similarities were analyzed using blast among ST239, ST8, and ST30. Alignment of ST239, ST8 and ST30 isolates against the reference genome TW20 (ST239, GenBank accession: FN433596.1) was performed using Snippy version 4.6.0 (https://github.com/tseemann/snippy) to generate a core-genome SNP alignment, and the recombination events were detected using Gubbins version 2.4.1 (*80*). For ST59, using M013 (GenBank accession: CP003166.2) as a representative sequence, the top 500 strains most similar to its genome were identified using Mash version 2.3 (*89*). A phylogenetic tree was constructed to analyze these strains alongside our study’s ST59 MRSA genomes. The recombination analysis methods for ST59 were consistent with those applied to ST239.

### Competition experiments

Competition experiments between ST59 and ST239 were performed as previously described (*10*). Forty MRSA isolates (20 ST59 and 20 ST239; detailed strain information is listed in table S3) were cultured individually on tryptic soy agar (TSA) plates and incubated at 37°C for 24 hours. Single colonies from each strain were inoculated into 4 ml of tryptic soy broth (TSB) and grown overnight at 37°C with shaking (220 rpm). Equal volumes (1 ml) from each culture were pooled to generate a mixed bacterial suspension, which was thoroughly homogenized. Genomic DNA was extracted and purified from 10 ml of the initial mixture, followed by whole-genome sequencing as previously described for 1244 MRSA isolates. For the competition step, 6 ml of the pooled culture was diluted 1:50 in fresh TSB and incubated at 37°C for 24 hours under the same shaking conditions. After incubation, genomic DNA was again extracted from 10 ml of the postcompetition culture and subjected to sequencing. Isolate-specific SNPs were identified from the multi-isolate core-genome SNP matrix generated by mapping raw reads to the ST239 reference TW20 (GenBank accession: FN433596.1) and calling SNPs with Snippy version 4.6.0 (https://github.com/tseemann/snippy); per-isolate markers are provided in table S4. Relative strain abundance before and after competition was quantified from read counts supporting isolate-specific SNP alleles after mapping to the TW20 reference, and the competitive index for each isolate was calculated as the log ratio of its relative abundance after versus before incubation. Raw competitive index values were normalized using the scale function in R. All competition experiments were performed in three independent biological replicates, and the difference in competitive index between ST239 and ST59 was analyzed using a two-sided Mann-Whitney *U* test.

### Independent growth assay

Strains were cultured overnight in TSB and diluted to an optical density at 600 nm (OD_600_) of 0.05, followed by incubation at 37°C with 200 rpm shaking. OD_600_ was measured every 2 hours for the first 12 hours, then at 20, 22, and 24 hours. Growth rates were calculated using the R package Growthcurver version 0.3.1 (*90*).

### Pan-genome analysis of ST59 and ST239 clones

The pan-genome of each clone, comprising core and accessory genes, was analyzed using Panaroo version 1.3.0 with default parameters (*86*). Gene accumulation curves were visualized via ggplot2, and alpha values were estimated using the heaps function in micropan version 2.1. An alpha >1 indicated a closed pan-genome, whereas an alpha <1 suggested an open pan-genome (*14*, *91*). PCA of the gene presence-absence matrix was conducted using Adegenet version 2.0.1, with Euclidean distances calculated from 431 principal components explaining 95% of variation. Gene annotations were performed using eggNOG-mapper (COG), InterProScan (GO), and KAAS (pathways) (*92*–*95*).

### Lactate dehydrogenase assay for assessing MRSA toxicity on human monocytic cells THP-1

To assess MRSA toxicity on THP-1 cells via the lactate dehydrogenase (LDH) assay, all ST59 (*n* = 401) and ST239 (*n* = 184) isolates were tested. The THP-1 human monocyte-macrophage cell line (ATCC no. TIB-202, 3 × 10^6^ cells/ml) were differentiated into macrophages with phorbol 12-myristate 13-acetate in six-well plates. After 3 hours, cells were washed and incubated in medium with 10% fetal bovine serum (FBS), without antibiotics. MRSA was adjusted to 1.0 × 10^9^ CFU/ml, and 10 μl of the bacterial suspension was added per well [multiplicity of infection (MOI) = 30]. After 6 hours of incubation at 37°C, cell culture supernatants were collected by centrifugation, and LDH release was measured using an LDH assay kit.

### Genome-wide association analysis

To identify potential virulence-related genes carried by ST59 but not by ST239, we used pyseer version 1.3.10 (*96*) software. We used the built-in linear mixed model to fit the presence/absence matrix of candidate virulence-related genes for all ST59 and ST239 strains [this binary matrix was identified from the VFDB setB database (accessed in July 2023)] to the corresponding strains’ measurements of THP-1 lysis. The software’s included count_patterns.py script was used to calculate the likelihood ratio test (lrt) *P* value threshold. In this study, all genes with lrt *P* values <0.0019 were considered statistically significant.

### Sequence difference analysis of shared virulence genes in ST59 and ST239 clones

Python scripts were used to extract shared virulence genes identified in the ST59 and ST239 clone strains. Gene sequence clustering was performed using cd-hit version 4.8.1 software (*97*) with parameters set to -c 1. Subsequently, representative virulence gene sequences were aligned using Prank version 170427 (*98*). Differences in virulence gene sequences between the two clones were confirmed manually. To identify the protein structural domains carried by SraP in the ST59 and ST239 clones, we used the previously reported SraP crystal structure (*99*) and the NCBI CD-search program (*100*). These domains were visualized using the DOG local program (*101*). A comparative analysis of the genetic environments upstream and downstream of the *sraP* gene was visualized using the Matplotlib package in Python (https://python.org/).

### Mouse nasal colonization model

Six-week-old female BALB/c mice (*n* = 10 per strain) received 20 μl of PBS containing 1 × 10^8^ strains into their nasal passages. After 3 days, mice were euthanized, nasal tissues were homogenized, and counts were determined by plating 200 μl of diluted tissue suspension on TSA.

### Mice BSI model

Eight-week-old female BALB/c mice were injected with 100 μl of bacterial suspension (10 mice per strain). Mice were monitored every 24 hours, and all survivors were euthanized after 10 days. Bacterial burden in the liver, heart, lungs, and kidney was assessed by plating tissue suspensions on TSA.

### Mouse skin infection model

Bacterial strains were cultured in TSB at 37°C for 9 hours to reach the postexponential phase, then adjusted to 1 × 10^8^ CFU/100 μl. Anesthetized BALB/c nude female mice (6 weeks old) received a 100-μl subcutaneous injection into the buttock region using a 27-gauge needle. Injection sites were disinfected with 70% ethanol. Skin lesion development was monitored for 7 days, with lesion size measured daily using digital calipers (length and width).

### Construction of Δ*chp* and Δ*sraP* mutants and their complemented strains

Deletion mutants of *chp* and *sraP* (Δ*chp* and Δ*sraP*) were constructed using the temperature-sensitive shuttle plasmid pKOR1. Their complemented mutants, compΔ*chp* and compΔ*sraP* were constructed by using the pLI50 shuttle vector.

### Cell adhesion assay

Human dermal fibroblasts and A549 alveolar epithelial cells (3 × 10^6^ cells/ml) were seeded in six-well plates and cultured to 90% confluence. After supplementation with high-glucose RPMI 1640 medium containing 10% FBS for 3 hours, bacterial strains were cultured in TSB for 6 hours, then adjusted to 1.0 × 10^9^ CFU/ml. Ten microliters of the bacterial suspension (MOI = 30) was added to each well and coincubated at 37°C for 6 hours. After washing with PBS, cells were lysed with 0.1% sodium deoxycholate, diluted, and plated for colony counting.

### Coculture with neutrophils

The bacterial suspension was adjusted to 5 × 10^8^ CFU/ml. One-hundred microliters of neutrophils (5 × 10^7^ cells/well) in RPMI/HSA were added to a 96-well plate precoated with 50% human serum in PBS for 1 hour. Following this, 100 μl of RPMI/HSA and bacterial cultures (5 × 10^7^ CFU/ml, MOI = 100) were added, and incubation was carried out at 37°C with 5% CO_2_ for 0.5, 1, 1.5, and 2 hours. Afterward, 22 μl of 1% saponin was added to lyse neutrophils, allowing for CFU counting to assess bacterial survival in serum (*102*).

### Blood survival assay

Fifty microliters of bacterial suspension (2.5 × 10^6^ CFU) was mixed with 450 μl of human blood and incubated at 37°C for 1 hour. The samples were treated with PBS, 0.5% saponin, streptokinase, trypsin, and deoxyribonuclease I to lyse cells and disperse bacterial clumps. Surviving CFUs were quantified as a percentage of the original inoculum, with mutant strain survival normalized to WT survival for each donor, given variability in WT survival (10 to 70%) (*103*).

### Expression, purification, and gel filtration of CHIPS

The pET28a vector was digested with BamH I and Xho I, and the polymerase chain reaction product was ligated to obtain the recombinant plasmid pET28a-*chp*, which was verified by DNA sequencing. The plasmid was transformed into BL21 (DE3) cells, and a 1-liter LB culture was grown at 37°C. After cooling to 16°C, isopropyl-β-d-thiogalactopyranoside was added to induce expression for 12 hours. The CHIPS protein was purified using elution buffer, analyzed by SDS–polyacrylamide gel electrophoresis (PAGE; 12%), and stored at −80°C.

### Mouse neutrophil influx model

The assay was performed as described by de Haas *et al.* (*51*). Eight- to 10-week-old C57 mice were subjected to pretreatment via intravenous injection with 100 μl of PBS containing varying doses of CHIPS (0, 5, 10, and 20 μg). After a 15-min interval, the mice were intraperitoneally challenged with 10 μg of human C5a. Following a 3-hour duration, the mice were euthanized, peritoneal lavage was performed, and the number of emitted neutrophils was assessed. Flow cytometry was used for analysis.

### Comparison of whole-blood survival rate in CHIPS-treated and untreated ST239

To evaluate the role of CHIPS in immune evasion, a CHIPS-expressing plasmid was electroporated into CHIPS-negative *S. aureus* ST239 strains. Following confirmation of expression, the WT strains and CHIPS-ST239 were incubated in freshly collected and heparinized human whole blood at 37°C for 1 hour with gentle rotation. Bacterial survival was quantified by serial dilution and plating.

### Hydrogen peroxide susceptibility

Susceptibility to hydrogen peroxide was assessed using a disc diffusion method. A 100-μl sample of mid-log-phase bacterial culture was spread evenly on a TSA plate and dried. Sterile 6-mm filter paper discs, each saturated with 10 μl of 5%, 7.5%, or 15% H_2_O_2_, were placed on the plate. After 24 hours of incubation at 37°C, the diameters of the inhibition zones were measured in millimeters.

### Desiccation resistance test

The capacity of MRSA strains to survive desiccation was assessed as previously described (*3*). The survival rates of bacteria at the designated end time points (6 hours, 24 hours, 5 days, and 7 days) were calculated.

### Thermotolerance assay

The capacity of heat resistance for MRSA strains was assessed as previously described (*104*). All bacterial strains were grown to reach the mid-log phase and subsequently adjusted to an OD_600_ of 0.3. Each culture was then split into two separate portions. One portion was kept at 37°C, while the other was incubated at 48°C for 30 min. Postincubation, 100-μl aliquots from each portion were taken, serially diluted in PBS, and then spread onto TSA followed by incubation at 37°C for 24 hours. Subsequently, both portions were exposed to a temperature of 60°C for 5 min. After this heat treatment, 100-μl aliquots were again removed, diluted in PBS, plated onto TSA, and incubated at 37°C for another 24 hours. Last, number of CFUs was counted.

### Growth at high osmolarity

Bacterial cultures were grown to mid-log phase and adjusted to an OD_600_ of 0.2. Subsequently, 100-μl aliquots were spread onto TSA with or without 2.2 M NaCl. After incubating at 37°C for 48 hours, colonies were counted (*64*).

### Growth at acid/basic pH

The procedure was the same as the independent growth assays, with the modification that TSB was adjusted to pH 3.0, 4.0, 4.5, 5.5, 6.5, and 7.4 using hydrochloric acid, respectively. The OD_600_ was measured every 2 hours for the first 12 hours, then at 20, 22, and 24 hours.

### RNA sequencing and bioinformatics analysis

RNA libraries were constructed using the TruSeq RNA Sample preparation Kit (Illumina, San Diego, CA, USA) and sequenced on the HiSeq 4000 platform (Illumina, San Diego, CA, USA) to obtain 150 bp paired-end reads. The filtered clean data were mapped to the genome of ST59 representative strain SKLX15777 using HISAT2 version 2.2.1 (*105*), and gene count matrices were obtained using featureCounts version 2.0.1 (*106*). Differentially expressed genes (DEGs) were identified using DESeq2 (*107*) (for pH 4.5 versus pH 7.4: log_2_|fold change| >2 and false discovery rate–adjusted *P* < 0.05). Gene expression heatmaps were plotted using the variance-stabilizing transformation-transformed data. The DEGs were then subjected to GO and KEGG enrichment analysis using clusterProfiler version 4.6.0 (*108*).

### Quantification and statistical analysis

All data were analyzed using GraphPad Prism 9. Sample sizes (*n*), exact tests, and any additional details are provided in the figure legends. Kaplan-Meier survival curves were compared using the log-rank test. Pairwise comparisons of continuous variables were performed with the two-sided Student’s *t* test or Mann-Whitney *U* test, as appropriate. Correlations between cytotoxicity, murine virulence measures, and acid tolerance were assessed using Spearman’s rank correlation analysis. Each independent experiment was performed with at least three biological replicates. Statistical significance was set at *P* < 0.05.

## Supplementary Materials

### This PDF file includes:

The BRICS Working Group Figs. S1 to S13 Tables S1 to S4
