# Supplementary material for: Reduced MGE burden, virulence optimization, and acid stress tolerance shape the clonal succession of MRSA ST59
Source: Sci Adv. 2026 Mar 11;12(11):eaeb3121. doi: 10.1126/sciadv.aeb3121 (PMC12978222; doi:10.1126/sciadv.aeb3121)
Supplement: Supplementary file 1 — The BRICS Working Group Figs. S1 to S13 Tables S1 to S4 [file sciadv.aeb3121_sm.pdf]

Science Advances
AAAS

Supplementary Materials for

Reduced MGE burden, virulence optimization, and acid stress tolerance shape the clonal succession of MRSA ST59

Ye Jin *et al.*

Corresponding author: Beiwen Zheng, zhengbw@zju.edu.cn; Yonghong Xiao, xiaoyonghong@zju.edu.cn

Sci. Adv. **12**, eaeb3121 (2026)
DOI: 10.1126/sciadv.aeb3121

This PDF file includes:

The BRICS Working Group
Figs. S1 to S13
Tables S1 to S4

the BRICS Working Group

Xiao Yonghong[6], Yunbo Chen[6], Ji Jinru[6], Liu Zhiying[6], Ying Chaoqun[6], Yang Qing[6], Kong Haishen[6], Song Jiangqin[7], Ding Hui[8], Li Yanyan[9], Dai Yuanyuan[10], Mao Haifeng[11], Tian Pengpeng[12], Wang Lu[13], Liu Yongyun[14], Zhou Yizheng[15], Wang Jiliang[16], Jin yan[17], Huang Donghong[18], Xu Hongyun[19], Zhang Peng[20], Qiang Xinhua[21], He hong[22], Zheng lin[23], Cao Junmin[24], Liu zhou[25], Huang Ying[26], Geng Yan[27], Kang Haiquan[28], Liu Dan[29], Liao Guolin[30], Zhang Lixia[31], Chen Fenghong[32], Li Yanhong[33], Zhang Baohua[34], Qiang Xinhua[35], Li Xiaoyan[36], Liu Donghua[37], Zhang Qiuying[38], Hu xuefei[39], Guo Liang[40], Man Sijin[41], Song Dijing[42], Xu Rong[43], Yin Youdong[44], Liang Kunpeng[45], Li aiyun[46], Li zhuo[47], Liu Qiang[48], Lu guoping[49], Liang Jinhua[50], Liu Qiang[51], Dong yinjiao[52], Shen jilu[53], Hu shuyan[54], Luan liang[55], Li jian[56], Meng ling[57], Qiao dengyan[58], Xia xiusan[59], Quan bo[60], Wang dahong[61], Han chunhua[62], Yan xiaoping[63], Li fei[64], Wang shifu[65]

[6]The First Affiliated Hospital, Zhejiang University School of Medicine, Hangzhou, China. [7]Clinical Laboratory, the First People's Hospital of Tianmeng, Tianmeng, China. [8]Clinical Laboratory, Lishui Central Hospital, Lishui, China. [9]Clinical Laboratory, Zibo Central Hospital, Zibo, China. [10]Clinical Laboratory, Anhui Provincial Hospital, Hefei, China. [11]Clinical Laboratory, the First People's Hospital of Lianyungang, Lianyungang, China. [12]Clinical Laboratory, the First People's Hospital of Jingzhou, Jingzhou, China. [13]Clinical Laboratory, Lu'an People's Hospital, Lu'an, China. [14]Clinical Laboratory, Binzhou Medical University Hospital, Binzhou, China. [15]Clinical Laboratory, Jingzhou Central Hospital, Jingzhou, China. [16]Clinical Laboratory, Shengli Oilfield Central Hospital, Dongying, China. [17]Clinical Laboratory, Shandong Provincial Hospital, Jinan, China. [18]Clinical Laboratory, the Second Affiliated Hospital of Fujian Medical University, Quanzhou, China. [19]Clinical Laboratory, the Second People's Hospital of Yunnan Province,

Kunming, China. [20]Clinical Laboratory, Yijishan Hospital of Wannan Medical College, Wuhu, China. [21]Clinical Laboratory, the First People's Hospital of Huzhou, Huzhou, China. [22]Clinical Laboratory, the Shinan campus Affiliated Hospital of Qingdao University, Qingdao, China. [23]Clinical Laboratory, The First Affiliated Hospital of Ningbo University, Ningbo, China. [24]Clinical Laboratory, Zhejiang Provincial Hospital of Traditional Chinese Medicine, Hangzhou, China. [25]Clinical Laboratory, The Second Affiliated Hospital of Anhui Medical University, Hefei, China. [26]Clinical Laboratory, First Affiliated Hospital of Anhui Medical University, Hefei, China. [27]Clinical Laboratory, the Second Affiliated Hospital of Xi'an Jiaotong University, Xi'an, China. [28]Clinical Laboratory, The Affiliated Hospital of Xuzhou Medical University, Xuzhou, China. [29]Clinical Laboratory, Jiujiang First People's Hospital, Jiujiang, China. [30]Clinical Laboratory, Wuhan Puren Hospital, Wuhan, China. [31]Clinical Laboratory, Shaanxi Provincial People's Hospital, Xi'an, China. [32]Clinical Laboratory, The First Hospital of Putian City, Putian, China. [33]Clinical Laboratory, Anyang District Hospital of Henan Province, Anyang, China. [34]Clinical Laboratory, People's Hospital of Huangshan City, Huangshan, China. [35]Clinical Laboratory, the Affiliated Hospital of Jining Medical University, Jining, China. [36]Clinical Laboratory, The Fifth Affiliated Hospital of Southern Medical University, GuangZhou, China. [37]Clinical Laboratory, Xiaogan Central Hospital, Xiaogan, China. [38]Clinical Laboratory, Suizhou Central Hospital, Suizhou, China. [39]Clinical Laboratory, The First Affiliated Hospital of Nanchang University, Nanchang, China. [40]Clinical Laboratory, Mindong Hospital of Ningde City, Fu'an, China. [41]Clinical Laboratory, Tengzhou Central People's Hospital, Tengzhou, China. [42]Clinical Laboratory, People's Hospital of Haining City, Haining, China. [43]Clinical Laboratory, People's Hospital of Yichun City, Yichun, Jiangxi Province, China. [44]Clinical Laboratory, the People's Hospital of Yingjiang, Yingjiang, China. [45]Clinical Laboratory, The Second Affiliated Hospital of BengBu Medical University,

Bengbu, China. [46]Clinical Laboratory, Women's Hospital, Zhejiang University School of Medicine, Hangzhou, China. [47]Clinical Laboratory, the First Affiliated Hospital of Xi'an Medical University, Xi'an, China. [48]Clinical Laboratory, Huaihe Hospital of Henan University, Kaifeng, China. [49]Clinical Laboratory, Fuyang Hospital of Anhui Medical University, Fuyang, China. [50]Clinical Laboratory, the Affiliated Hongqi Hospital of Mudanjiang Medicine College, Mudanjiang, China. [51]Clinical Laboratory, the First Affiliated Hospital of He'nan University of Science and Technology, Luoyang, China. [52]Clinical Laboratory, Tianchang People's Hospital, Tianchang, China. [53]Clinical Laboratory, The Fourth Hospital of Ahhui Medical University, Hefei, China. [54]Clinical Laboratory, People's Hospital of Qingyang, Qingyang, China. [55]Clinical Laboratory, General Hospital of Northern Theater Command, Shenyang, China. [56]Clinical Laboratory, Lu'an Civil Hospital, Lu'an, China. [57]Clinical Laboratory, The Second Hospital of Lanzhou University, Lanzhou, China. [58]Clinical Laboratory, Gansu Provincial Hospital of Traditional Chinese Medicine, Lanzhou, China. [59]Clinical Laboratory, People's Hospital of Lujiang, Caohu, China. [60]the second People's Hospital Hospital of Jingzhou, Jingzhou, China. [61]Clinical Laboratory, Hunan Normal University Affiliated Xiangdong Hospital, Liling, China. [62]Clinical Laboratory, the Xihaian campus Affiliated Hospital of Qingdao University, Qingdao, China. [63]Clinical Laboratory, Zigong Third People's Hospital, Zigong, China. [64]Clinical Laboratory, Women's Hospital of Luan Jinan District, Lu'an, China. [65]Clinical Laboratory, Children's Hospital of Qilu, Jinan, China.

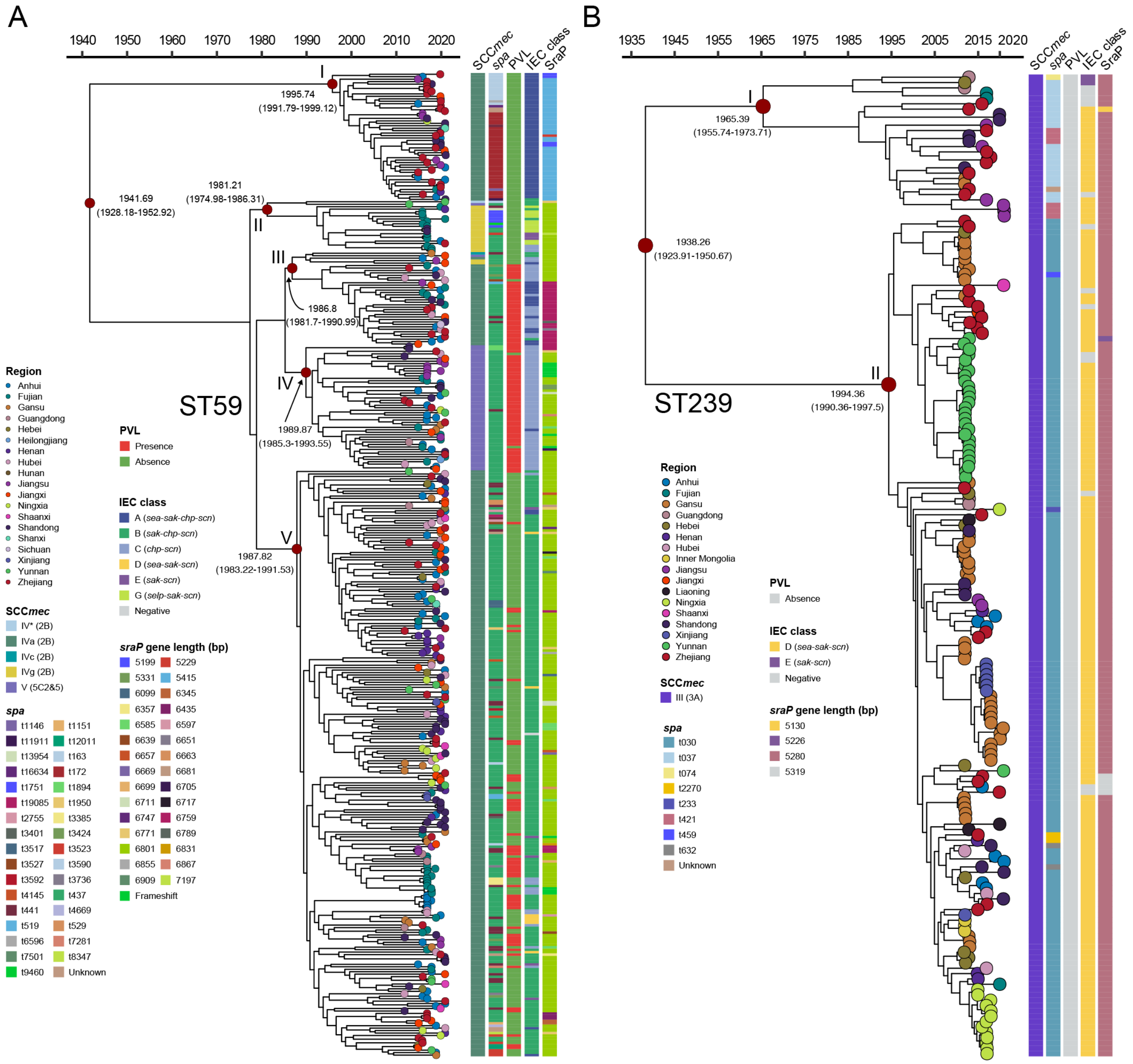

Fig. S1. Dated phylogeny of ST59 and ST239 in this study. Time-based tree for ST59 (n = 401) (A) and ST239 (n = 184) (B). The characteristics of each strain are displayed on the right, including SCC*mec* types, *spa* types, presence/absence of PVL, immune evasion cluster (IEC) gene classes and *sraP* gene length (bp). The geographical origins of isolates are mapped on the tips, and divergence time of each clade is shown around the nodes with 95% HPD intervals in parentheses.

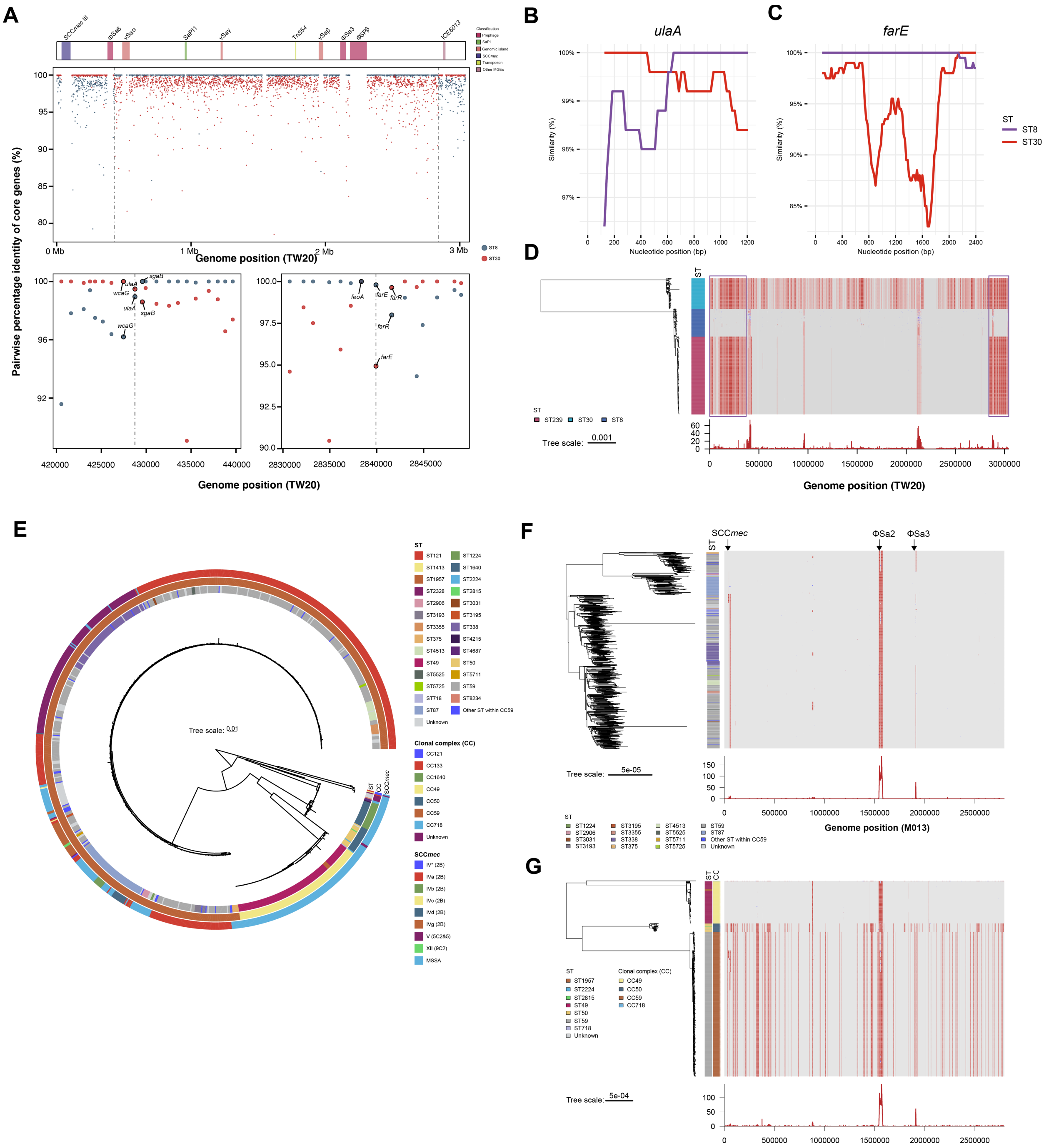

Fig. S2. Comparative analysis of recombination and core genome similarity in MRSA lineages ST239 and ST59. (A) Distribution of core gene sequence similarity between ST239 and its parental lineages ST8 (blue) and ST30 (red). Each dot represents a core gene, positioned according to its location on the ST239 reference genome (TW20). Vertical gray dashed lines indicate inferred recombination boundaries. Annotations above the plot denote the positions and categories of mobile genetic elements (MGEs), color-coded by type. (B-C) Gene-level sequence similarity

analysis for *ulaA* (B) and *farE* (C), comparing ST8 (purple) and ST30 (red) to ST239. These genes demarcate recombination breakpoints, supporting the chimeric nature of the ST239 chromosome arising from large-scale chromosomal replacement events. (D) Phylogenetic relationships and recombination landscape among representative ST239, ST8, and ST30 isolates. The left panel presents a maximum-likelihood tree, while the adjacent heatmap depicts recombination density and donor-recipient exchanges, highlighting the extensive mosaicism within the ST239 genome. (E) Phylogenetic analysis of ST59 and its closely related clonal complexes. The circular phylogeny is annotated from the inside out with ST, clonal complex (CC), and SCC*mec* type. (F) Recombination events within the CC59 lineage. (G) Recombination analysis between CC50, CC49, and CC718 and the ST59 lineage, showing limited inter-lineage chromosomal exchange, consistent with the recombination-sparse evolution of ST59. The line plots below the recombination heatmaps (D, F and G) display the frequency of recombination events along the reference genome.

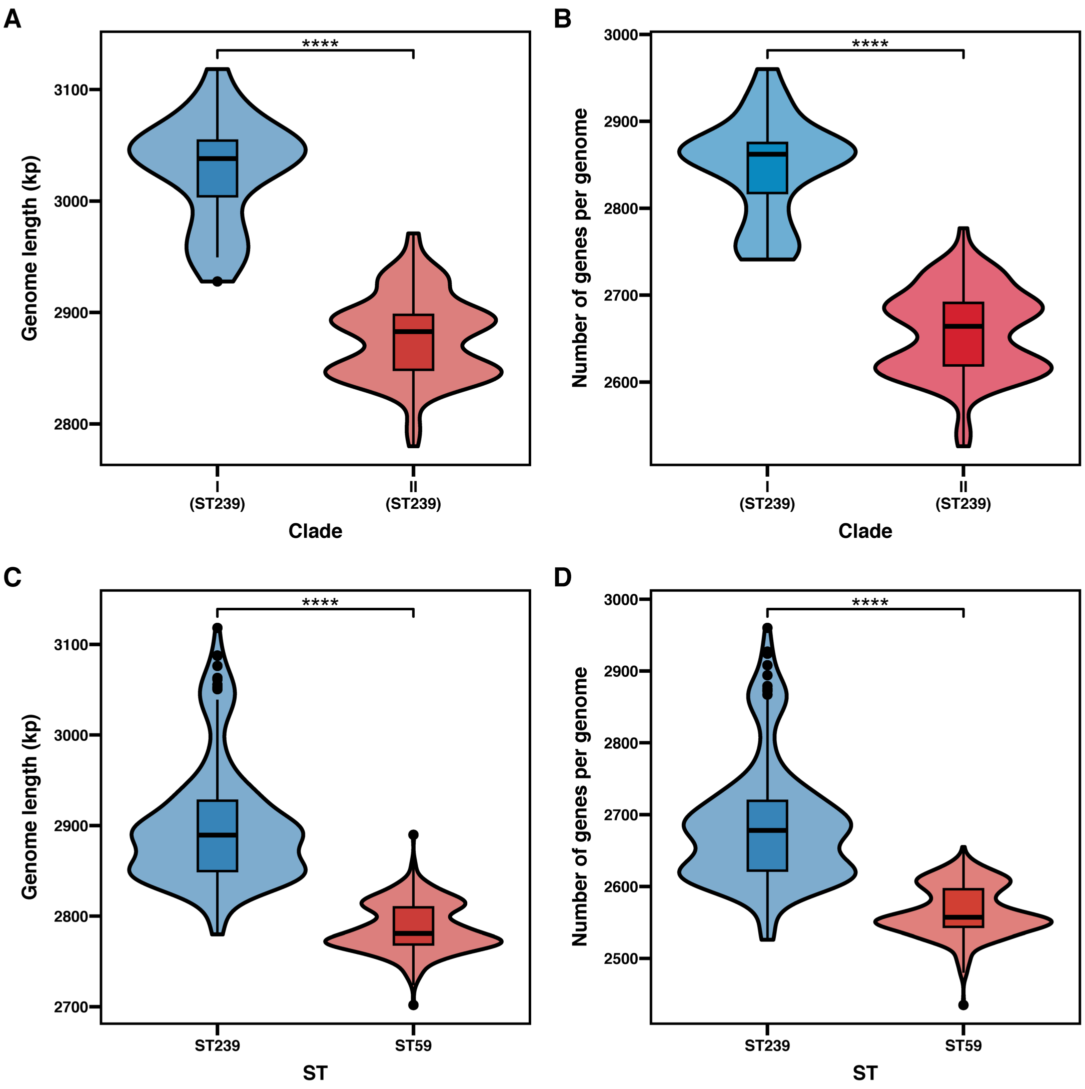

Fig. S3. ST239 and ST59 genome alignments. (A) Distribution of genome size between two clads of ST239 (ST239-I: n = 27; ST239-II: n = 157). (B) Distribution of gene number between two clads of ST239 (ST239-I: n = 27; ST239-II: n = 157). (C) Distribution of genome size between ST239 (n = 184) and ST59 (n = 401). (D) Distribution of gene number between (n = 184) and ST59 (n = 401). *p* values are provided following the two-sided Mann-Whitney U test (A-D). ****, $p < 0.0001$.

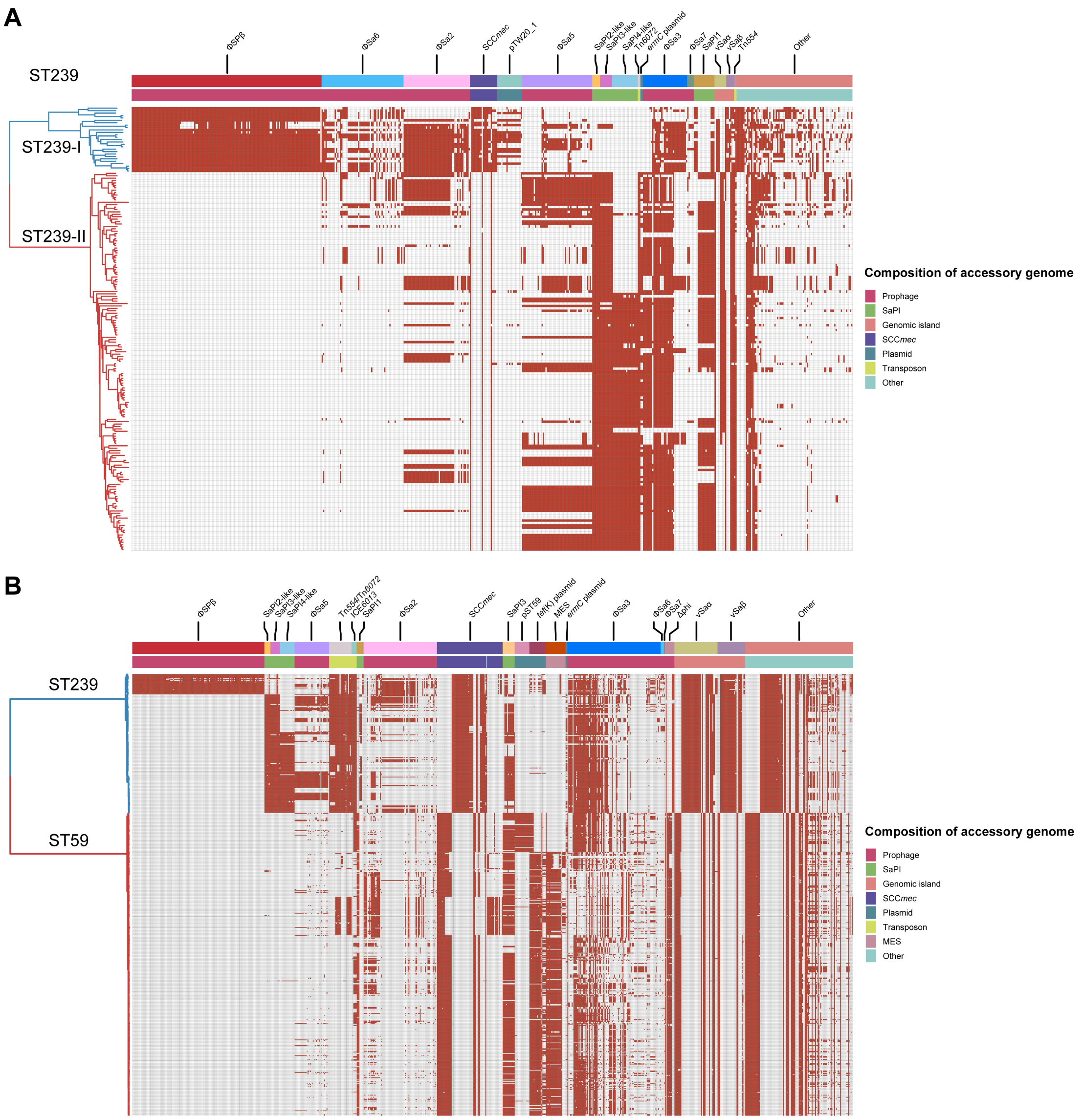

Fig. S4. Accessory genome composition profile for two MRSA lineages. (A) Difference in accessory genome composition between the two clades of ST239 (ST239-I: n = 27; ST239-II: n = 157). (B) Difference in accessory genome composition between ST239 (n = 184) and ST59 (n = 401) lineages. Red squares indicate the presence of the gene examined. The tips of the phylogenetic tree on the left correspond to the strains in the heatmap. The two annotation bars above the heatmap indicate, from top to bottom, the MGE category of accessory genes (per the legend at right) and the specific MGEs carrying them. Because some accessory genes can occur on multiple MGEs, each such gene is assigned to a single MGE category for display to avoid double counting. Accessory genes with prevalence ≥80% or ≤10% within a clade (A) or lineage (B) are considered too conserved or too rare and are excluded from further analysis.

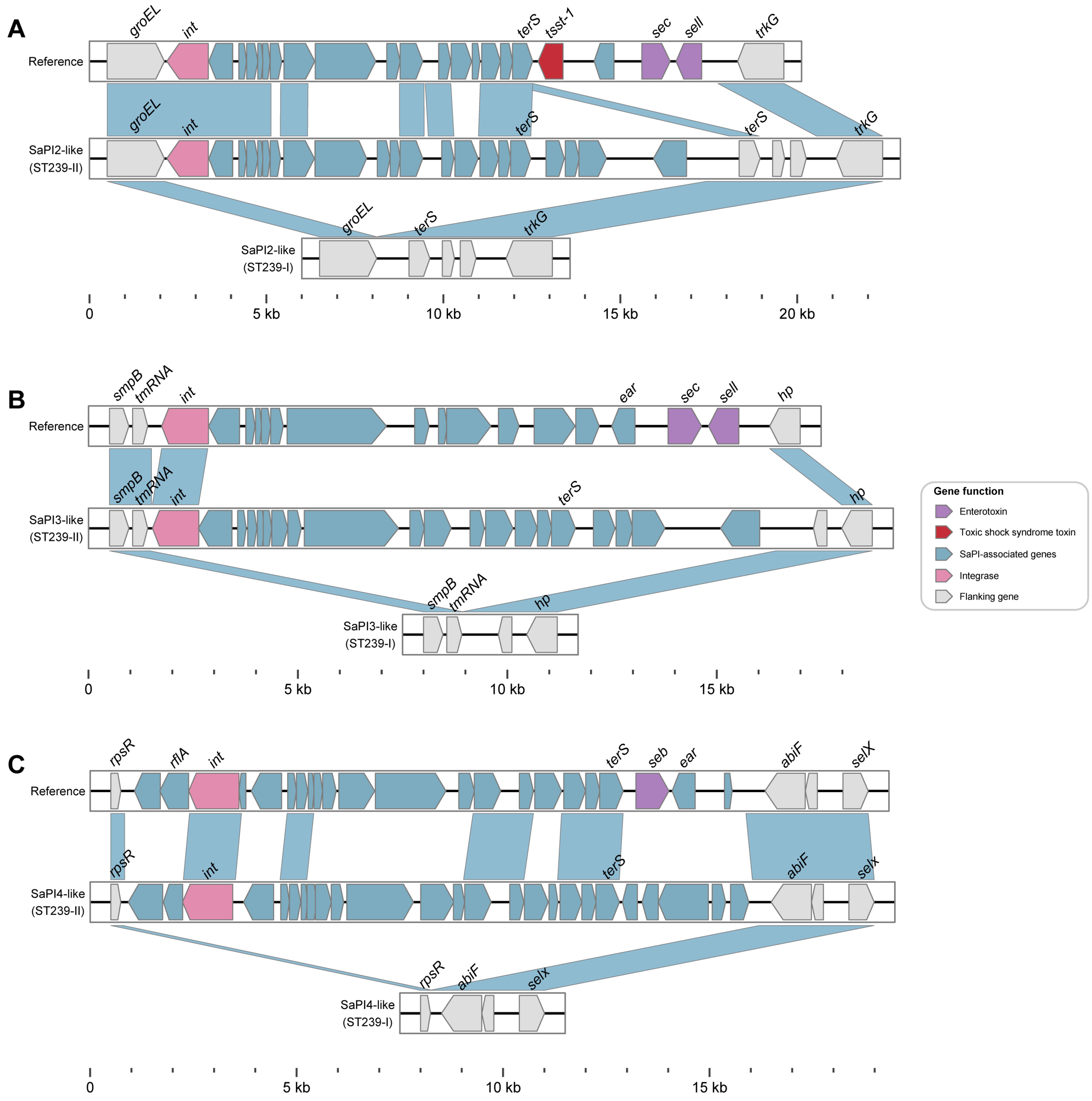

Fig. S5. Schematic view of the SaPI2/3/4-like elements identified in the ST239-II, and its linear comparison with related regions.

(A) Comparison of the SaPI2-like with related regions. (B) Comparison of the SaPI3-like with related regions. (C) Comparison of the SaPI4-like with related regions. Genes are represented by arrowed boxes and colored based on gene function classification. Shading denotes regions of homology (nucleotide identity ≥85%).

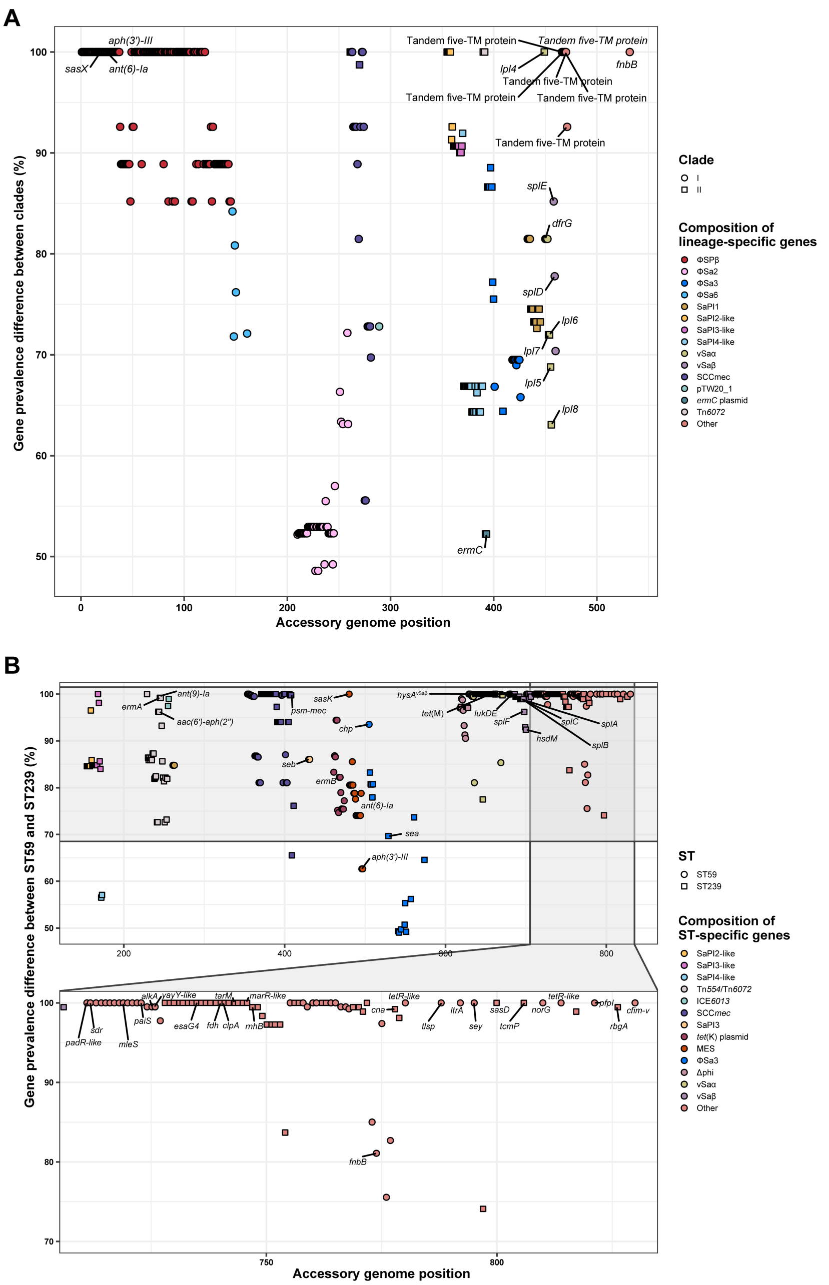

Fig. S6. Differences in prevalence of specific genes. (A) Differences in prevalence of clade-specific genes between two clades of ST239 (ST239-I: n = 27; ST239-II: n = 157). (B) Differences in prevalence of lineage-specific genes between ST59 (n = 401) and ST239 (n = 184). Each point signifies a unique gene and is colored based on its classification. The shape of the point corresponds to the clade or lineage to which the gene is specific. In each panel, the x-axis indicates gene position, ordered identically to Fig. S4. The y-axis shows the absolute difference in gene

prevalence between the compared clades or lineages. Genes with standard names are labeled adjacent to their loci.

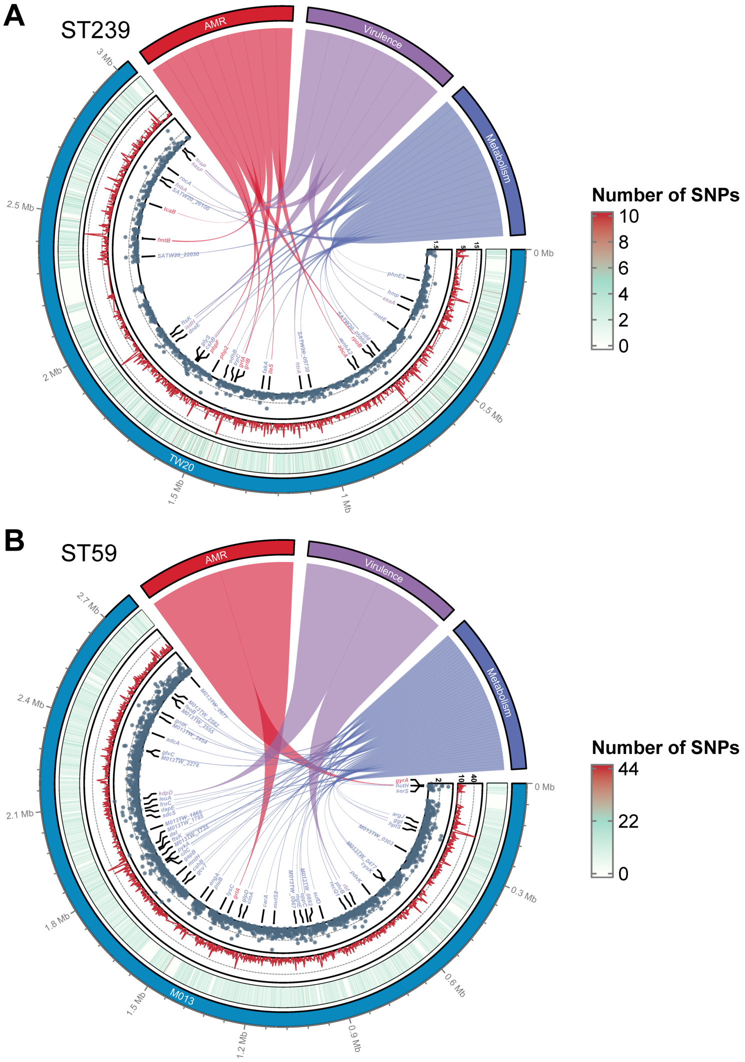

Fig. S7. Positive selection analysis for two MRSA lineages. Genes under positive selection in ST239 (n = 184) (A) and ST59 (n= 401) (B). From innermost to outermost: the innermost circle represents the distribution of dN/dS values (non-synonymous to synonymous substitution rate), with each point indicating the dN/dS value of a gene. The dashed line represents the positions where dN/dS = 1.5 (ST239) or 2 (ST59). The second circle displays the distribution of SNP numbers per gene, with each point showing the number of SNPs occurring on a gene; the positions of the dashed line are labeled alongside. The third circle illustrates genome-wide SNP density, using a sliding window of 1000. Genes labeled with their names are those under positive selection. These genes are color-coded based on their respective functional categories and are connected to their corresponding functional regions.

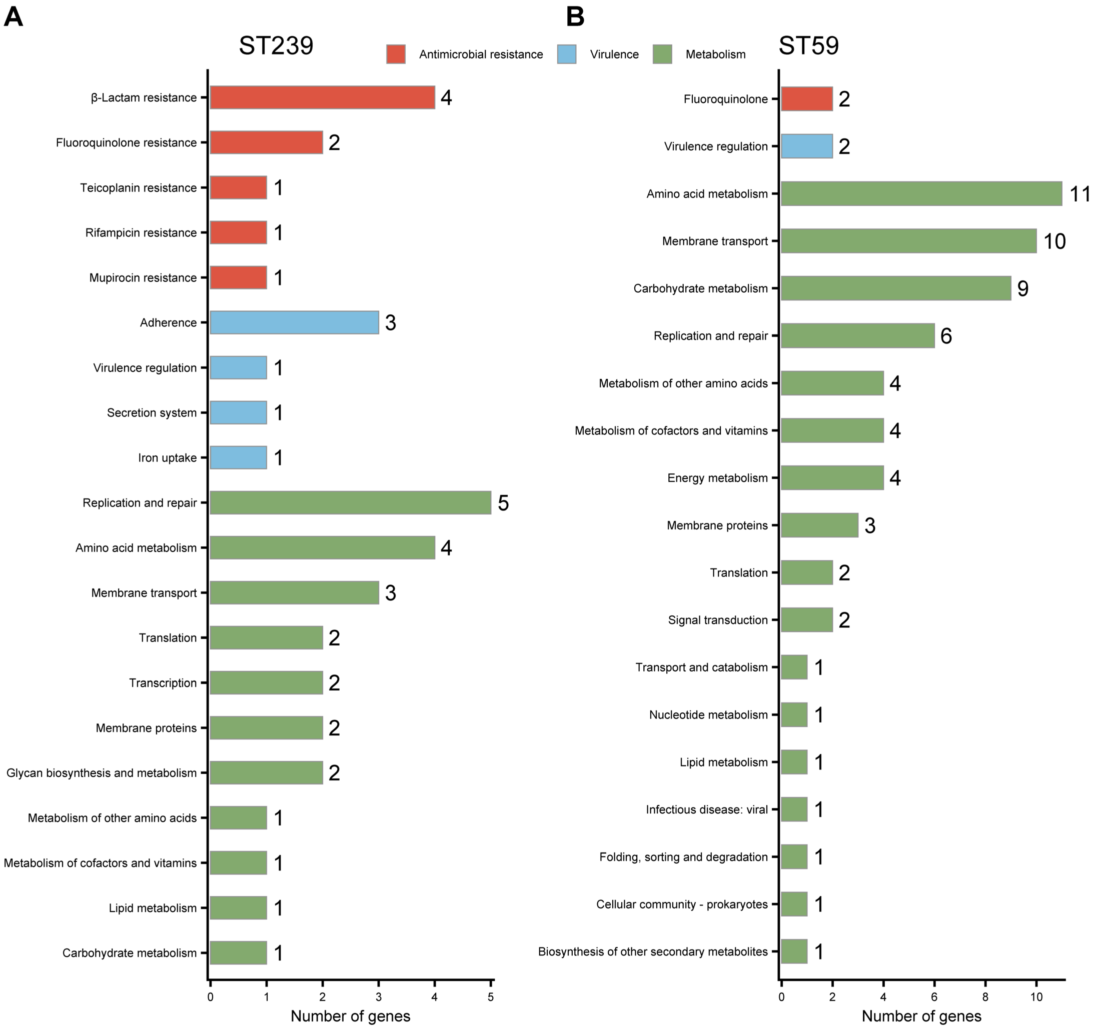

Fig. S8. Functional annotation of candidate genes. Functional annotation of candidate genes under positive selection in ST239 (A) and ST59 (B). Gene counts for each functional category are displayed to the right of the corresponding bars.

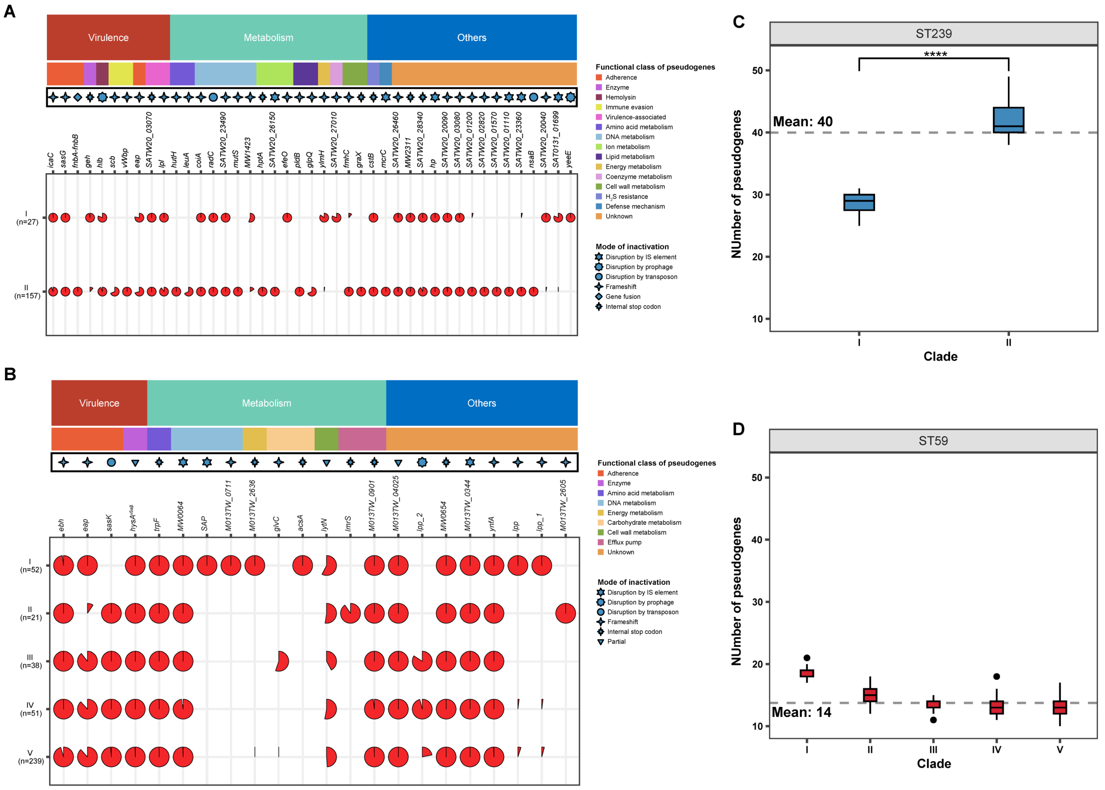

Fig. S9. ST239 undergoes more extensive pseudogenization than ST59. (A) Distribution of pseudogenes between clades of ST239. (B) Distribution of pseudogenes between clades of ST59. For each clade in both lineages, the pie chart shows the fraction of isolates in which the specified gene is pseudogenized (red sector). The pseudogenization type and functional category are displayd above the pie chart and match the legend at right. (C) The number of pseudogenes between clades of ST239. Two-sided Mann-Whitney U test. ****, $p < 0.0001$. (D) The number of pseudogenes between clades of ST59.

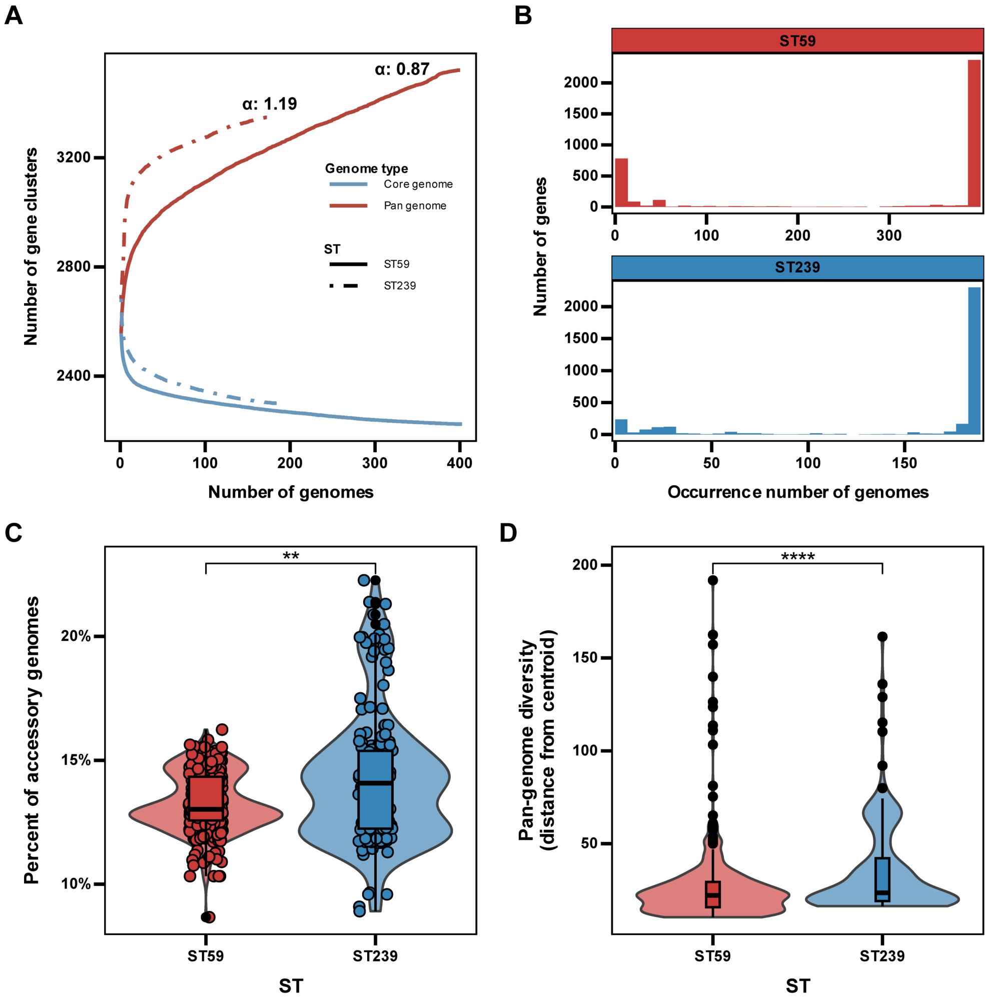

Fig. S10. ST239 Displays Greater Gene Content Diversity but Reduced Evolutionary Plasticity. (A) Pan-genomes of the ST59 (n = 401) and ST239 (n = 184) lineages. The cumulative curves depict the relationship between the size of the pan-genome and core genome and the number of genomes. The α values associated with each lineage's pan-genome curve represent the open/closed state of that lineage's pan-genome. An alpha value of > 1 indicates an open pan-genome of the lineage, whereas an alpha value of < 1 indicates a closed pan-genome. (B) Distribution of homologous gene families in ST59 (n = 401, upper) and ST239 (n = 184, lower) strains is presented. (C) Distribution of accessory genome proportion within each strain of the ST59 (n = 401) and ST239 (n = 184) lineages. Two-sided Mann-Whitney U test (ST59 vs. ST239). (D) Distribution of the Euclidean distance between each strain and the lineage centroid is shown for ST59 and ST239. Two-sided Mann-Whitney U test (ST59 vs. ST239). **, $p < 0.01$; ****, $p < 0.0001$.

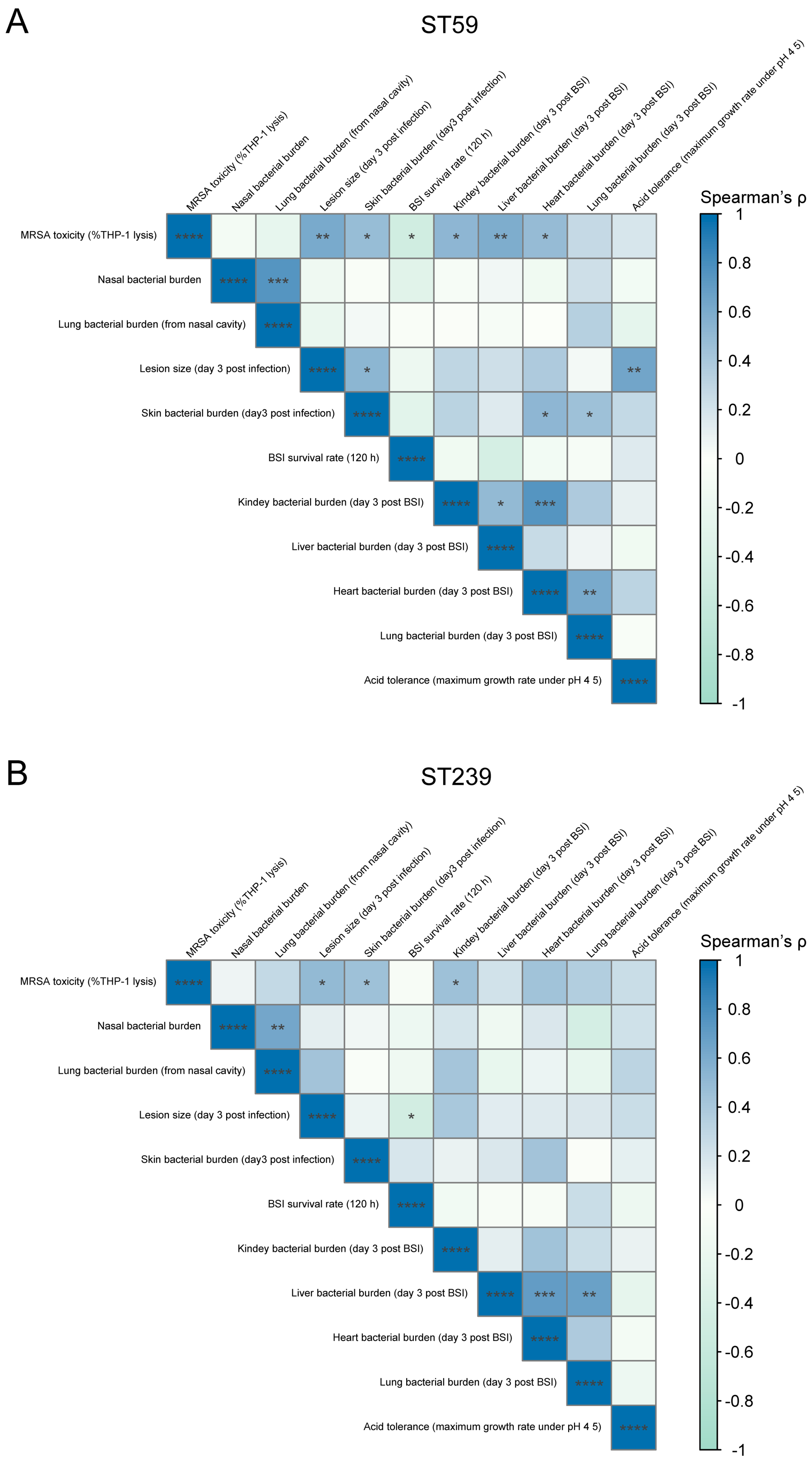

Fig. S11. Lineage-stratified correlations between cytotoxicity, murine virulence measures, and acid tolerance. Heatmaps show Spearman's rank correlations for ST59 (A) and ST239 (B). Variables

include MRSA cytotoxicity (% THP-1 lysis), nasal bacterial burden, lung bacterial burden after nasal inoculation, lesion size at day 3, skin bacterial burden at day 3, BSI survival rate at 120 h, kidney/liver/heart/lung bacterial burdens at day 3 after BSI, and acid tolerance (maximum growth rate at pH 4.5). Asterisks denote significant correlations (*, $p < 0.05$; **, $p < 0.01$; ***, $p < 0.001$; ****, $p < 0.0001$); cells without asterisks are not significant. Cell color indicates Spearman's correlation coefficient (ρ).

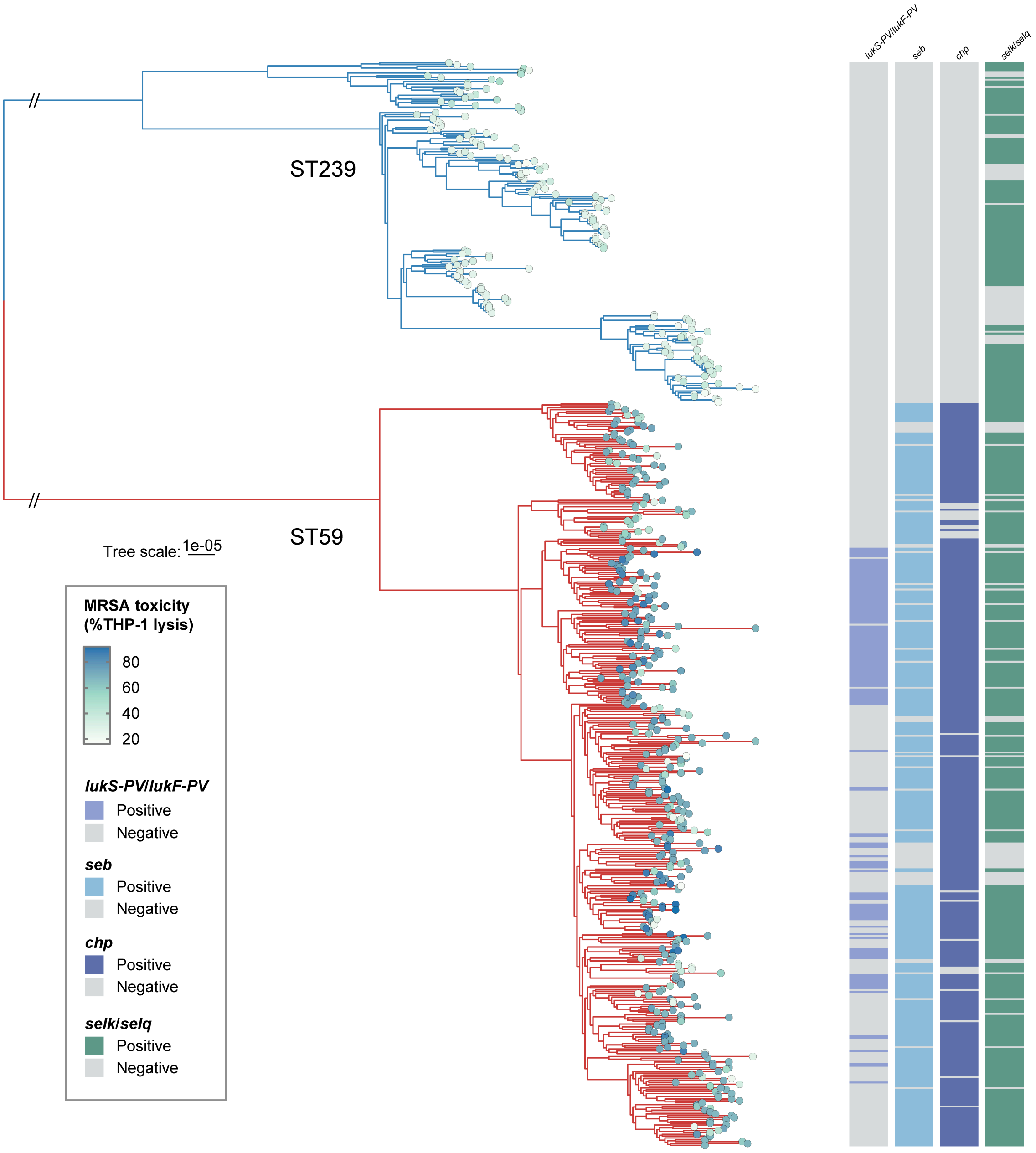

Fig. S12. Cytotoxicity mapped onto the core-genome phylogeny of ST59 and ST239. Tree tips are colored by MRSA cytotoxicity (% THP-1 lysis) for all ST59 (n = 401) and ST239 (n = 184) isolates. Right-hand-side bars indicate presence/absence of GWAS-associated virulence genes (*lukS-PV*/*lukF-PV*, *seb*, *chp* and *selk*/*selq*). For clarity of presentation, disproportionately long branches are truncated and denoted by break symbols; this modification improves legibility only and does not affect the inferred topology (the scale bar applies to unbroken branch lengths).

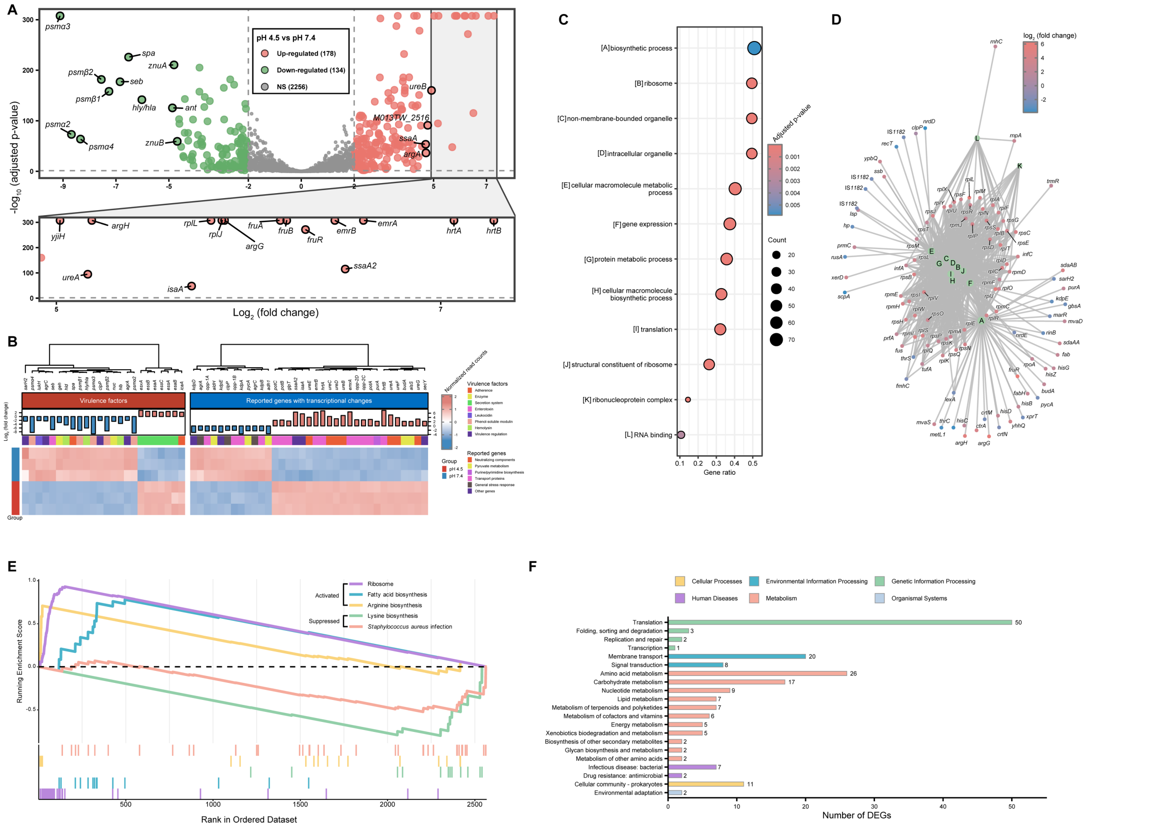

Fig. S13. Transcriptome diversity of ST59 at acidic (pH = 4.5) and physiological pH (pH = 7.4). (A) Volcano plots of the gene expression profile for differentially expressed genes (DEGs). Genes meeting only the fold change ($\log_2$|fold change| >2) or statistical significance cutoffs (false discovery rate-adjusted $p < 0.05$) are shown in green (down-regulated) and red (up-regulated), respectively. Genes that exhibited no differences are in gray. The top30 DEGs are labeled on the plot. (B) Heatmap displaying the expression of virulence genes and reported genes with transcriptional changes between ST59 at acidic (pH = 4.5) and physiological pH (pH = 7.4). (C) GO enrichment analysis of DEGs. The x-axis shows the proportion of DEGs annotated to each GO term relative to the total number of DEGs annotated to GO terms; the y-axis lists the corresponding GO terms. Dot size indicates the number of DEGs in each term, and dot color represents the enrichment *p* value (D) Relationship between enriched GO terms (Green-filled letters) and DEGs (each node)..Gray lines connect genes to associated terms; node color reflects $\log_2$ (fold change). (E) Gene set enrichment analysis based on KEGG pathways. (F) KEGG annotations of DEGs.

Table S1. Metadata of 1,244 MRSA from 2011 to 2020.

Strain	Accession	Clonal complex	ST	SCC*mec*	*spa*	Year
SKLX50577	JBAHCZ000000000	CC59	ST59	IVa (2B)	t163	2014
SKLX50637	JBAHCB000000000	CC5	ST5	II (2A)	t002	2014
SKLX52177	JBAHRQ000000000	CC5	ST5	II (2A)	t002	2014
SKLX53440	JBAHPE000000000	CC5	ST5	II (2A)	t002	2015
SKLX53480	JBAHLR000000000	CC5	ST5	II (2A)	t002	2015
SKLX55077	JBAGYF000000000	CC5	ST5	II (2A)	t311	2015
SKLX56231	JBAGUP000000000	CC5	ST5	II (2A)	t17573	2016
SKLX61098	JBAGZV000000000	CC5	ST5	II (2A)	t002	2017
SKLX63559	JBAHQE000000000	CC5	ST5	II (2A)	t2460	2018
SKLX63565	JBAHRZ000000000	CC5	ST5	II (2A)	t2460	2018
SKLX83693	JBAHFX000000000	CC5	ST5	II (2A)	t002	2018
SKLX50712	JBAHCH000000000	CC59	ST59	IVa (2B)	t172	2014
SKLX83700	JBAHFJ000000000	CC5	ST5	II (2A)	t2460	2018
SKLX83810	JBAHKV000000000	CC5	ST5	IVg (2B)	t688	2018
SKLX87248	JBAHIO000000000	CC5	ST5	II (2A)	t311	2018
SKLX50717	JBAHCA000000000	CC59	ST59	IVa (2B)	t172	2014
SKLX50788	JBAHFG000000000	CC59	ST59	IVa (2B)	t441	2014
SKLX51063	JBAHTE000000000	CC59	ST59	IVa (2B)	t437	2014
SKLX51492	JBAHSN000000000	CC59	ST59	V (5C2&5)	t437	2014
SKLX51493	JBAHSY000000000	CC59	ST59	IVa (2B)	t437	2014
SKLX53476	JBAHPQ000000000	CC59	ST59	IVa (2B)	t437	2015
SKLX53478	JBAHMC000000000	CC59	ST59	IVa (2B)	t437	2015
SKLX53479	JBAHLS000000000	CC59	ST59	V (5C2&5)	t437	2015
SKLX53484	JBAHLT000000000	CC59	ST59	IVa (2B)	t163	2015
SKLX50636	JBAHEW000000000	CC59	ST338	V (5C2&5)	t437	2014
SKLX53487	JBAHLK000000000	CC59	ST59	IVa (2B)	t437	2015
SKLX50714	JBAHFL000000000	CC88	ST88	IVc (2B)	t2310	2014
SKLX53611	JBAHMA000000000	CC59	ST59	IVa (2B)	t437	2015
SKLX50775	JBAHBQ000000000	CC1	ST1	IVc (2B)	t1508	2014
SKLX53973	JBAHQK000000000	CC59	ST59	IVa (2B)	t437	2015
SKLX50832	JBAHCY000000000	CC88	ST88	ΨSCC*mec*$_{ST88}$	t7637	2014
SKLX53974	JBAHMB000000000	CC59	ST59	IVa (2B)	t437	2015
SKLX54052	JBAHML000000000	CC59	ST59	IVa (2B)	t437	2015
SKLX54459	JBAGZZ000000000	CC59	ST59	IVa (2B)	t3424	2015
SKLX51240	JBAHTH000000000	CC8	ST239	III (3A)	t030	2014
SKLX54479	JBAHCL000000000	CC59	ST59	IVa (2B)	t437	2015

SKLX55797	JBAGSI000000000	CC59	ST59	IVa (2B)	t437	2015
SKLX51620	JBAHSD000000000	CC45	ST45	IVa (2B)	t116	2014
SKLX56945	JBAGVW000000000	CC59	ST59	IVa (2B)	t437	2016
SKLX57229	JBAHHJ000000000	CC59	ST59	IVa (2B)	t437	2016
SKLX57230	JBAHFO000000000	CC59	ST59	IVa (2B)	t437	2016
SKLX52179	JBAHQA000000000	CC88	ST88	ΨSCC*mec*$_{ST88}$	None	2014
SKLX57233	JBAHII000000000	CC59	ST59	IVa (2B)	t437	2017
SKLX57234	JBAHGH000000000	CC59	ST59	IVa (2B)	t437	2016
SKLX52805	JBAHOX000000000	CC398	ST5539	V (5C2)	t034	2014
SKLX57249	JBAHJB000000000	CC59	ST59	IVa (2B)	t437	2016
SKLX52880	JBAHNQ000000000	CC398	ST398	V (5C2)	t034	2015
SKLX57252	JBAHEC000000000	CC59	ST59	IVa (2B)	t437	2016
SKLX57866	JBAHIL000000000	CC59	ST59	IVa (2B)	t437	2016
SKLX58002	JBAHUN000000000	CC59	ST59	V (5C2&5)	t437	2016
SKLX58004	JBAHSR000000000	CC59	ST59	V (5C2&5)	t437	2016
SKLX58350	JBAHTJ000000000	CC59	ST59	IVa (2B)	t437	2016
SKLX53474	JBAHQQ000000000	CC398	ST5539	V (5C2)	t034	2015
SKLX58725	JBAHRK000000000	CC59	ST59	V (5C2&5)	t437	2016
SKLX53477	JBAHLW000000000	CC5	ST5529	IVg (2B)	t688	2015
SKLX59439	JBAHLA000000000	CC59	ST59	IVa (2B)	t3517	2016
SKLX59887	JBAHBC000000000	CC59	ST59	IVa (2B)	t437	2016
SKLX59892	JBAHAS000000000	CC59	ST59	IVa (2B)	t441	2016
SKLX60349	JBAGVU000000000	CC59	ST59	IVa (2B)	t441	2017
SKLX61303	JBAGSB000000000	CC59	ST59	V (5C2&5)	t437	2017
SKLX61304	JBAGVM000000000	CC59	ST59	IVa (2B)	t437	2017
SKLX53588	JBAHOG000000000	CC8	ST239	III (3A)	t030	2015
SKLX61339	JBAGVO000000000	CC59	ST59	IVa (2B)	t7281	2017
SKLX53655	JBAHRJ000000000	CC8	ST630	V (5C2&5)	t4549	2015
SKLX63323	JBAHRM000000000	CC59	ST59	IVa (2B)	t172	2018
SKLX63327	JBAHUL000000000	CC59	ST59	IVa (2B)	t3523	2018
SKLX63333	JBAHRG000000000	CC59	ST59	IVa (2B)	t3517	2018
SKLX63907	JBAHKH000000000	CC59	ST59	IVa (2B)	t4669	2018
SKLX63908	JBAHLM000000000	CC59	ST59	V (5C2&5)	t437	2018
SKLX81816	JBAHGS000000000	CC59	ST59	V (5C2&5)	t437	2018
SKLX54055	JBAHKY000000000	CC121	ST121	V (5C2&5)	t8660	2015
SKLX54320	JBAHCO000000000	CC8	ST239	III (3A)	t030	2015
SKLX82898	JBAHGA000000000	CC59	ST59	IVa (2B)	t437	2018
SKLX82910	JBAHFZ000000000	CC59	ST59	IVa (2B)	t437	2018

SKLX82911	JBAHJU000000000	CC59	ST59	IVa (2B)	t437	2018
SKLX83688	JBAHLJ000000000	CC59	ST59	IVa (2B)	t441	2018
SKLX83809	JBAHHB000000000	CC59	ST59	IVa (2B)	t437	2018
SKLX83818	JBAHEY000000000	CC59	ST59	IVa (2B)	t437	2018
SKLX94245	JBAHNW000000000	CC59	ST59	IVa (2B)	t163	2019
SKLX95565	JBAHQJ000000000	CC59	ST59	IVa (2B)	t437	2019
SKLX97572	JBAHNO000000000	CC59	ST59	IVg (2B)	t437	2019
SKLX100319	JBAGUK000000000	CC59	ST59	V (5C2&5)	t437	2019
SKLX107461	JBAGYN000000000	CC59	ST59	V (5C2&5)	t437	2019
SKLX54839	JBAHAB000000000	CC88	ST6309	IVa (2B)	t8296	2015
SKLX107516	JBAGXA000000000	CC59	ST59	IVa (2B)	t437	2019
SKLX117513	JBAHNA000000000	CC59	ST59	IVa (2B)	t172	2019
SKLX55793	JBAGWI000000000	CC398	ST3681	XIV (5A)	t1255	2015
SKLX55795	JBAGYW000000000	CC398	ST3681	XIV (5A)	t1255	2015
SKLX56176	JBAGSP000000000	CC7	ST7	V (5C2&5)	t796	2016
SKLX56222	JBAGVX000000000	CC1	ST1	IVg (2B)	t114	2016
SKLX56713	JBAGSD000000000	CC8	ST239	III (3A)	t030	2016
SKLX56991	JBAGSV000000000	CC59	ST5525	IVa (2B)	t437	2016
SKLX56993	JBAGWU000000000	CC59	ST5525	IVa (2B)	t437	2016
SKLX57237	JBAHGD000000000	CC398	ST398	V (5C2)	t034	2016
SKLX57238	JBAHET000000000	CC398	ST398	V (5C2)	t034	2016
SKLX57282	JBAHGY000000000	CC59	ST338	V (5C2&5)	t437	2016
SKLX57794	JBAHDU000000000	CC59	ST5525	IVa (2B)	t437	2016
SKLX57801	JBAHFP000000000	CC8	ST630	V (5C2&5)	t4549	2016
SKLX58009	JBAHSU000000000	CC8	ST239	III (3A)	t030	2016
SKLX58278	JBAHTD000000000	CC398	ST398	V (5C2)	t4652	2016
SKLX58283	JBAHTI000000000	CC5	ST764	II (2A)	t002	2016
SKLX58351	JBAHTA000000000	CC59	ST338	V (5C2&5)	t437	2016
SKLX58368	JBAHSZ000000000	CC88	ST88	IVc (2B)	t2310	2016
SKLX58369	JBAHHW000000000	CC8	ST72	IVc (2B)	t664	2016
SKLX59891	JBAHCE000000000	CC88	ST88	IVc (2B)	t2310	2016
SKLX60371	JBAGWS000000000	CC59	ST338	V (5C2&5)	t441	2017
SKLX60404	JBAHCD000000000	CC398	ST398	V (5C2)	t034	2017
SKLX60713	JBAGWR000000000	CC5	ST5529	IVg (2B)	t688	2017
SKLX61321	JBAGRZ000000000	CC88	ST88	ΨSCC*mec*$_{ST88}$	t7637	2017
SKLX61340	JBAGSA000000000	CC509	ST509	IVa (2B)	t375	2017
SKLX61370	JBAGSR000000000	CC88	ST88	IVc (2B)	t2310	2017
SKLX62287	JBAHIH000000000	CC5	ST8479	II (2A)	t9353	2017

SKLX63041	JBAHRE000000000	CC88	ST88	ΨSCC*mec*$_{ST88}$	t14340	2018
SKLX63045	JBAHRD000000000	CC88	ST88	ΨSCC*mec*$_{ST88}$	t14340	2018
SKLX63326	JBAHQN000000000	CC8	ST630	V (5C2&5)	t4549	2018
SKLX63329	JBAHRW000000000	CC88	ST88	ΨSCC*mec*$_{ST88}$	t14340	2018
SKLX63331	JBAHSE000000000	CC5	ST764	II (2A)	t002	2018
SKLX63580	JBAHPW000000000	CC398	ST398	V (5C2)	t034	2018
SKLX63877	JBAHJQ000000000	CC398	ST398	V (5C2)	t034	2018
SKLX82906	JBAHFK000000000	CC398	ST398	V (5C2)	t034	2018
SKLX83133	JBAHER000000000	CC398	ST398	V (5C2)	t2876	2018
SKLX83686	JBAHES000000000	CC8	ST239	III (3A)	t030	2018
SKLX83689	JBAHJD000000000	CC8	ST239	III (3A)	t030	2018
SKLX83694	JBAHHI000000000	CC5	ST764	II (2A)	t002	2018
SKLX83812	JBAHDQ000000000	CC59	ST-	IVa (2B)	t172	2018
SKLX87346	JBAHDI000000000	CC1	ST1	IVa (2B)	t127	2018
SKLX88605	JBAHPY000000000	CC398	ST398	V (5C2)	t034	2019
SKLX89512	JBAHON000000000	CC398	ST398	V (5C2)	t034	2019
SKLX91455	JBAHQX000000000	CC88	ST88	IVc (2B)	t2310	2019
SKLX91456	JBAHPL000000000	CC88	ST88	ΨSCC*mec*$_{ST88}$	None	2019
SKLX91461	JBAHPF000000000	CC88	ST88	ΨSCC*mec*$_{ST88}$	None	2019
SKLX94087	JBAHPG000000000	CC5	ST764	II (2A)	t002	2019
SKLX97564	JBAHNM000000000	CC88	ST88	ΨSCC*mec*$_{ST88}$	None	2019
SKLX99406	JBAHJC000000000	CC1	ST1	IVa (2B)	t127	2019
SKLX99408	JBAHJE000000000	CC8	ST630	V (5C2)	t4549	2019
SKLX100209	JBAHMN000000000	CC59	ST5317	IVa (2B)	t437	2019
SKLX100316	JBAHFA000000000	CC22	ST22	V (5C2&5)	t309	2019
SKLX100539	JBAGWX000000000	CC22	ST22	V (5C2&5)	t309	2019
SKLX103058	JBAHFE000000000	CC398	ST398	V (5C2)	t034	2019
SKLX103060	JBAHUC000000000	CC45	ST45	IVa (2B)	t776	2019
SKLX107637	JBAGWD000000000	CC398	ST398	V (5C2)	t011	2019
SKLX108426	JBAHTM000000000	CC398	ST398	V (5C2)	t034	2019
SKLX114107	JBAGXF000000000	CC398	ST398	V (5C2)	t034	2019
SKLX114126	JBAGXU000000000	CC398	ST398	V (5C2)	t034	2019
SKLX114637	JBAGYM000000000	CC398	ST1232	V (5C2&5)	t034	2019
SKLX114770	JBAGWT000000000	CC22	ST8466	IVb (2B)	t309	2019
SKLX117514	JBAHBB000000000	CC30	ST30	IVc (2B)	t019	2019
SKLX51800	JBAHQW000000000	CC88	ST88	ΨSCC*mec*$_{ST88}$	t10793	2014
SKLX54694	JBAGZN000000000	CC45	ST508	IVi (2B)	t015	2015
SKLX54710	JBAGZF000000000	CC8	ST630	V (5C2)	t4549	2015

SKLX54735	JBAGZD000000000	CC45	ST508	IVi (2B)	t1608	2015
SKLX51784	JBAHTO000000000	CC59	ST59	IVa (2B)	t437	2014
SKLX51793	JBAHQZ000000000	CC59	ST59	V (5C2&5)	t437	2014
SKLX53030	JBAHLN000000000	CC59	ST59	IVa (2B)	t437	2015
SKLX53755	JBAHLI000000000	CC59	ST59	IVg (2B)	t1751	2015
SKLX53781	JBAHPC000000000	CC59	ST59	IVg (2B)	t1751	2015
SKLX54693	JBAGYV000000000	CC59	ST59	IVa (2B)	t441	2015
SKLX55539	JBAGSM000000000	CC45	ST508	IVi (2B)	t1608	2016
SKLX54695	JBAGZM000000000	CC59	ST59	V (5C2&5)	t437	2015
SKLX55555	JBAGUB000000000	CC5	ST965	IVc (2B)	t062	2016
SKLX54720	JBAHDJ000000000	CC59	ST59	IVa (2B)	t437	2015
SKLX54743	JBAGYU000000000	CC59	ST59	IVg (2B)	t1751	2015
SKLX54749	JBAGZW000000000	CC59	ST59	V (5C2&5)	t437	2015
SKLX54750	JBAHBF000000000	CC59	ST59	IVa (2B)	t437	2015
SKLX54751	JBAGZA000000000	CC59	ST59	IVg (2B)	t9460	2015
SKLX55486	JBAGWG000000000	CC59	ST59	IVa (2B)	t441	2016
SKLX55499	JBAGYP000000000	CC59	ST59	IVa (2B)	t441	2016
SKLX55521	JBAGWW000000000	CC59	ST59	V (5C2&5)	t437	2016
SKLX55554	JBAGSF000000000	CC59	ST59	IVa (2B)	t441	2016
SKLX56569	JBAGXQ000000000	CC59	ST59	IVa (2B)	t437	2016
SKLX56593	JBAGSY000000000	CC121	ST121	V (5C2&5)	t8660	2016
SKLX56609	JBAGUU000000000	CC59	ST338	V (5C2&5)	t437	2016
SKLX56578	JBAGVQ000000000	CC59	ST59	IVg (2B)	t1751	2016
SKLX56579	JBAGVC000000000	CC59	ST59	IVa (2B)	t3424	2016
SKLX56588	JBAGVH000000000	CC59	ST59	IVg (2B)	t1751	2016
SKLX56590	JBAGTZ000000000	CC59	ST59	V (5C2&5)	t437	2016
SKLX57645	JBAHEL000000000	CC59	ST59	IVa (2B)	t437	2016
SKLX57651	JBAHEK000000000	CC59	ST59	IVg (2B)	t437	2016
SKLX57656	JBAHEH000000000	CC59	ST59	IVg (2B)	t437	2016
SKLX57689	JBAHFF000000000	CC59	ST59	IVa (2B)	t437	2016
SKLX57696	JBAHEA000000000	CC59	ST59	IVa (2B)	t3385	2016
SKLX57702	JBAHFB000000000	CC59	ST59	IVa (2B)	t3385	2016
SKLX58708	JBAHRL000000000	CC59	ST59	IVa (2B)	t437	2016
SKLX57650	JBAHEJ000000000	CC45	ST508	IVi (2B)	t1608	2016
SKLX58711	JBAHTZ000000000	CC59	ST59	IVa (2B)	t437	2016
SKLX59491	JBAHKF000000000	CC59	ST59	IVa (2B)	t437	2016
SKLX59546	JBAHQO000000000	CC59	ST59	IVg (2B)	t437	2016
SKLX59550	JBAHNS000000000	CC59	ST59	IVa (2B)	t3736	2016

SKLX57701	JBAHEN000000000	CC88	ST88	PF-SCC	t12147	2016
SKLX60524	JBAGXN000000000	CC59	ST59	IVa (2B)	t437	2017
SKLX60530	JBAGYS000000000	CC59	ST59	IVa (2B)	t437	2017
SKLX60540	JBAGWP000000000	CC59	ST59	IVa (2B)	t437	2017
SKLX60555	JBAGUD000000000	CC59	ST59	IVa (2B)	t437	2017
SKLX63348	JBAHRB000000000	CC59	ST59	IVa (2B)	t437	2018
SKLX63372	JBAHQF000000000	CC59	ST59	IVa (2B)	t437	2018
SKLX78502	JBAGYJ000000000	CC59	ST59	IVa (2B)	t437	2018
SKLX58663	JBAHRR000000000	CC88	ST88	IVc (2B)	None	2016
SKLX78504	JBAGXY000000000	CC59	ST59	IVa (2B)	t441	2018
SKLX78523	JBAGXB000000000	CC59	ST59	IVa (2B)	t1146	2018
SKLX88663	JBAHTL000000000	CC59	ST59	V (5C2&5)	t437	2019
SKLX110352	JBAGUX000000000	CC59	ST59	IVa (2B)	t437	2019
SKLX117468	JBAHNZ000000000	CC59	ST59	V (5C2&5)	t437	2019
SKLX117483	JBAHTR000000000	CC59	ST59	IVg (2B)	t441	2019
SKLX59513	JBAHKB000000000	CC5	ST965	IVc (2B)	t062	2016
SKLX59525	JBAHLE000000000	CC45	ST508	IVi (2B)	t1608	2016
SKLX59537	JBAHJI000000000	CC8	ST239	III (3A)	t037	2016
SKLX59541	JBAHJL000000000	CC8	ST239	III (3A)	t037	2016
SKLX60519	JBAGVB000000000	CC5	ST965	IVc (2B)	t062	2017
SKLX60527	JBAGUG000000000	CC59	ST8362	IVa (2B)	t437	2017
SKLX60562	JBAGTT000000000	CC22	ST22	V (5C2&5)	t309	2017
SKLX78512	JBAGWQ000000000	CC9	ST9	XII (9C2)	t899	2018
SKLX82812	JBAHKO000000000	CC9	ST9	XII (9C2)	t899	2018
SKLX104993	JBAHUH000000000	CC6	ST6	IVa (2B)	t304	2019
SKLX105008	JBAHMV000000000	CC6	ST6	IVa (2B)	t304	2019
SKLX110362	JBAGWY000000000	CC88	ST88	ΨSCC*mec*$_{ST88}$	t6294	2019
SKLX115313	JBAHOT000000000	CC45	ST45	IVa (2B)	t116	2019
SKLX117470	JBAHRC000000000	CC5	ST965	IVc (2B)	t062	2019
SKLX55132	JBAGSU000000000	CC30	ST2580	IVc (2B)	t3351	2015
SKLX55133	JBAGXR000000000	CC30	ST2580	IVc (2B)	t3351	2015
SKLX55351	JBAGXM000000000	CC59	ST59	IVa (2B)	t437	2015
SKLX58897	JBAHSI000000000	CC59	ST59	IVa (2B)	t437	2016
SKLX58898	JBAHRT000000000	CC59	ST59	IVa (2B)	t437	2016
SKLX60779	JBAGUH000000000	CC59	ST59	IVa (2B)	t441	2017
SKLX60811	JBAHAR000000000	CC59	ST59	IVa (2B)	t437	2017
SKLX62660	JBAHUP000000000	CC59	ST59	IVa (2B)	t3523	2018
SKLX78468	JBAHCJ000000000	CC59	ST59	IVc (2B)	t441	2017

SKLX87136	JBAHDN000000000	CC59	ST59	V (5C2&5)	t437	2018
SKLX99456	JBAHIR000000000	CC59	ST59	V (5C2&5)	t437	2019
SKLX107244	JBAGZE000000000	CC59	ST59	IVa (2B)	None	2019
SKLX60806	JBAGVZ000000000	CC8	ST239	III (3A)	t030	2017
SKLX60813	JBAGVY000000000	CC8	ST239	III (3A)	t030	2017
SKLX60822	JBAGUQ000000000	CC8	ST239	III (3A)	t030	2017
SKLX60835	JBAGUI000000000	CC8	ST239	III (3A)	t030	2017
SKLX60840	JBAGUN000000000	CC8	ST239	III (3A)	t030	2017
SKLX61080	JBAGTD000000000	CC59	ST7025	IVa (2B)	t437	2017
SKLX62137	JBAHJT000000000	CC8	ST239	III (3A)	t030	2017
SKLX87194	JBAHEM000000000	CC59	ST8482	V (5C2&5)	t437	2018
SKLX99479	JBAHIZ000000000	CC8	ST239	III (3A)	t030	2019
SKLX99480	JBAHJZ000000000	CC8	ST239	III (3A)	t030	2019
SKLX105024	JBAHLU000000000	CC59	ST3355	IVa (2B)	t3523	2019
SKLX117548	JBAHBE000000000	CC59	ST3355	IVa (2B)	t437	2019
SKLX51104	JBAHRV000000000	CC8	ST239	III (3A)	t030	2014
SKLX52612	JBAHQS000000000	CC8	ST239	III (3A)	t030	2014
SKLX51897	JBAHRN000000000	CC59	ST59	IVa (2B)	t437	2014
SKLX53867	JBAHLY000000000	CC59	ST59	IVa (2B)	t437	2015
SKLX56513	JBAGUA000000000	CC59	ST59	IVa (2B)	t437	2016
SKLX59393	JBAHKX000000000	CC59	ST59	IVa (2B)	t437	2016
SKLX59394	JBAHLP000000000	CC59	ST59	IVa (2B)	t437	2016
SKLX59398	JBAHKI000000000	CC59	ST59	IVa (2B)	t437	2016
SKLX61197	JBAGVE000000000	CC59	ST59	IVa (2B)	t3401	2017
SKLX82849	JBAHGU000000000	CC59	ST59	IVa (2B)	t437	2018
SKLX100645	JBAHSP000000000	CC59	ST59	IVa (2B)	t437	2019
SKLX100648	JBAHPU000000000	CC59	ST59	IVa (2B)	t437	2019
SKLX109210	JBAGYR000000000	CC59	ST59	IVa (2B)	t437	2019
SKLX109214	JBAHJK000000000	CC59	ST59	IVa (2B)	t3592	2019
SKLX109215	JBAHTV000000000	CC59	ST59	IVa (2B)	t437	2019
SKLX109216	JBAHQM000000000	CC59	ST59	IVa (2B)	t437	2019
SKLX109407	JBAHTN000000000	CC59	ST59	IVa (2B)	t172	2019
SKLX115024	JBAGVT000000000	CC59	ST59	IVa (2B)	t19085	2019
SKLX63071	JBAHTG000000000	CC59	ST8480	V (5C2&5)	t437	2018
SKLX63091	JBAHUI000000000	CC398	ST398	V (5C2)	t034	2018
SKLX109209	JBAGUL000000000	CC30	ST30	V (5C2&5)	t318	2019
SKLX95326	JBAHOB000000000	CC5	ST5	II (2A)	t9353	2019
SKLX100675	JBAHBM000000000	CC59	ST59	V (5C2&5)	t437	2019

SKLX83971	JBAHEE000000000	CC8	ST72	IVc (2B)	t324	2018
SKLX83977	JBAHFI000000000	CC8	ST72	IVc (2B)	t324	2018
SKLX115288	JBAHOO000000000	CC1	ST1	IVa (2B)	t4494	2019
SKLX115296	JBAHOF000000000	CC1	ST1	IVa (2B)	t4494	2019
SKLX77838	JBAGYB000000000	CC5	ST5	II (2A)	t2460	2018
SKLX78407	JBAGYD000000000	CC5	ST5	II (2A)	t2460	2018
SKLX78421	JBAGYZ000000000	CC5	ST5	II (2A)	t2460	2018
SKLX78437	JBAGYX000000000	CC5	ST5	II (2A)	t2460	2018
SKLX56187	JBAGZP000000000	CC88	ST8474	V (5C2)	t1764	2016
SKLX56193	JBAGSK000000000	CC8	ST239	III (3A)	t030	2016
SKLX56215	JBAGXV000000000	CC59	ST4513	IVa (2B)	t441	2016
SKLX56216	JBAGSG000000000	CC8	ST239	III (3A)	t030	2016
SKLX57947	JBAHST000000000	CC1	ST1	IVg (2B)	t114	2016
SKLX57946	JBAHKJ000000000	CC59	ST59	IVa (2B)	t437	2016
SKLX59579	JBAHKT000000000	CC59	ST59	IVa (2B)	t437	2016
SKLX59589	JBAHJS000000000	CC59	ST59	IVa (2B)	t437	2016
SKLX59953	JBAHEO000000000	CC59	ST59	IVa (2B)	t437	2016
SKLX60328	JBAGZH000000000	CC59	ST59	IVa (2B)	t3527	2017
SKLX61251	JBAGXH000000000	CC59	ST59	IVa (2B)	t437	2017
SKLX61583	JBAHJW000000000	CC59	ST59	IVa (2B)	t437	2017
SKLX78422	JBAGZL000000000	CC59	ST59	IVa (2B)	t437	2018
SKLX80913	JBAGWF000000000	CC59	ST59	IVa (2B)	t437	2018
SKLX97560	JBAHNF000000000	CC59	ST59	IVa (2B)	t2755	2019
SKLX100083	JBAHQV000000000	CC59	ST59	IVa (2B)	t437	2019
SKLX100092	JBAHUF000000000	CC59	ST59	IVa (2B)	t437	2019
SKLX107417	JBAGSN000000000	CC59	ST59	IVa (2B)	t437	2019
SKLX61416	JBAGST000000000	CC398	ST398	V (5C2)	t034	2017
SKLX110276	JBAHAM000000000	CC59	ST59	IVa (2B)	t437	2019
SKLX63203	JBAHTX000000000	CC59	ST8366	IVa (2B)	t437	2018
SKLX85755	JBAHEZ000000000	CC59	ST3260	V (5C2&5)	t437	2018
SKLX88894	JBAHQB000000000	CC8	ST630	V (5C2&5)	t4549	2019
SKLX94652	JBAHMM000000000	CC398	ST398	V (5C2)	t034	2019
SKLX94661	JBAHOE000000000	CC398	ST398	V (5C2)	t034	2019
SKLX107301	JBAGZQ000000000	CC8	ST630	V (5C2&5)	None	2019
SKLX110300	JBAGUT000000000	CC45	ST508	IVi (2B)	t015	2019
SKLX113775	JBAHCM000000000	CC45	ST45	IVa (2B)	t1523	2019
SKLX113781	JBAHBZ000000000	CC30	ST30	IVc (2B)	t019	2019
SKLX116042	JBAHOA000000000	CC45	ST508	IVi (2B)	t026	2019

SKLX119086	JBAHBN000000000	CC188	ST188	IVa (2B)	t189	2019
SKLX79174	JBAGYI000000000	CC121	ST121	V (5C2&5)	t8660	2018
SKLX88955	JBAHPB000000000	CC88	ST88	IVc (2B)	t2310	2019
SKLX89362	JBAHTF000000000	CC121	ST946	IV (2B&5)	t758	2019
SKLX89369	JBAHQC000000000	CC59	ST8357	V (5C2&5)	t3592	2019
SKLX59908	JBAHAX000000000	CC5	ST5	II (2A)	t15309	2016
SKLX63734	JBAHTC000000000	CC5	ST5	II (2A)	t311	2018
SKLX50445	JBAHFR000000000	CC88	ST88	IVa (2B)	t3622	2014
SKLX52230	JBAHOR000000000	CC8	ST239	III (3A)	t030	2014
SKLX52232	JBAHOL000000000	CC398	ST398	V (5C2)	t034	2014
SKLX54120	JBAHAF000000000	CC8	ST239	III (3A)	t030	2015
SKLX54127	JBAHAO000000000	CC5	ST764	II (2A)	t002	2015
SKLX54652	JBAHAV000000000	CC8	ST239	III (3A)	t037	2015
SKLX55383	JBAGSX000000000	CC59	ST4513	IVa (2B)	t437	2016
SKLX55422	JBAGTJ000000000	CC5	ST8473	V (5C2&5)	t045	2016
SKLX56296	JBAGWK000000000	CC8	ST239	III (3A)	t037	2016
SKLX56310	JBAGSE000000000	CC398	ST398	V (5C2)	t034	2016
SKLX59931	JBAHAY000000000	CC59	ST338	V (5C2&5)	t437	2016
SKLX60471	JBAHAN000000000	CC8	ST72	IVc (2B)	t324	2017
SKLX61885	JBAHKZ000000000	CC398	ST398	V (5C2)	t571	2017
SKLX61912	JBAHLH000000000	CC88	ST88	IVc (2B)	t7480	2017
SKLX52742	JBAHMK000000000	CC59	ST59	IVa (2B)	t163	2014
SKLX53534	JBAHKQ000000000	CC59	ST59	IVa (2B)	t172	2015
SKLX53577	JBAHKU000000000	CC59	ST59	IVa (2B)	t172	2015
SKLX63254	JBAHRF000000000	CC59	ST8481	IVa (2B)	t163	2018
SKLX54590	JBAHAC000000000	CC59	ST59	IVa (2B)	t519	2015
SKLX55416	JBAGVJ000000000	CC59	ST59	V (5C2&5)	t437	2016
SKLX55421	JBAGSL000000000	CC59	ST59	V (5C2&5)	t437	2016
SKLX56302	JBAGYH000000000	CC59	ST59	V (5C2&5)	t437	2016
SKLX59942	JBAHAQ000000000	CC59	ST59	V (5C2&5)	t437	2016
SKLX60459	JBAGWM000000000	CC59	ST59	IVa (2B)	t437	2017
SKLX63744	JBAHUJ000000000	CC398	ST398	V (5C2)	t1928	2018
SKLX63752	JBAHTT000000000	CC1	ST1	IVa (2B)	t127	2018
SKLX63727	JBAHTK000000000	CC59	ST59	IVa (2B)	t172	2018
SKLX83072	JBAHHQ000000000	CC59	ST59	IVg (2B)	t437	2018
SKLX102503	JBAHGF000000000	CC59	ST59	V (5C2&5)	t437	2019
SKLX108650	JBAGZU000000000	CC59	ST59	V (5C2&5)	t437	2019
SKLX116102	JBAHOD000000000	CC59	ST59	IVa (2B)	t3424	2019

SKLX116114	JBAHPA000000000	CC59	ST59	V (5C2&5)	t437	2019
SKLX116126	JBAHOM000000000	CC59	ST59	IVa (2B)	t437	2019
SKLX116127	JBAHOJ000000000	CC59	ST59	IVa (2B)	t172	2019
SKLX83024	JBAHGL000000000	CC59	ST4513	IVa (2B)	t437	2018
SKLX83059	JBAHFV000000000	CC22	ST22	V (5C2&5)	t309	2018
SKLX89377	JBAHPN000000000	CC398	ST8484	V (5C2)	t034	2019
SKLX89397	JBAHSW000000000	CC59	ST3193	IVa (2B)	t172	2019
SKLX89446	JBAHPO000000000	CC398	ST8484	V (5C2)	t034	2019
SKLX102523	JBAHCN000000000	CC22	ST22	V (5C2&5)	t309	2019
SKLX108615	JBAHAD000000000	CC22	ST22	V (5C2&5)	t309	2019
SKLX108673	JBAGXX000000000	CC398	ST398	V (5C2)	t011	2019
SKLX108693	JBAGWJ000000000	CC398	ST398	V (5C2)	t1255	2019
SKLX116135	JBAHNV000000000	CC22	ST22	V (5C2&5)	t309	2019
SKLX116149	JBAHQU000000000	CC5	ST764	II (2A)	t002	2019
SKLX115178	JBAHOQ000000000	CC5	ST5	IVc (2B)	t010	2019
SKLX50944	JBAHUA000000000	CC8	ST239	III (3A)	t030	2014
SKLX57501	JBAHFY000000000	CC59	ST8371	IVa (2B)	t441	2016
SKLX58947	JBAHUD000000000	CC59	ST8364	IVa (2B)	t3523	2016
SKLX58958	JBAHLF000000000	CC59	ST8477	V (5C2&5)	t1751	2016
SKLX50938	JBAHSJ000000000	CC59	ST59	IVa (2B)	t437	2014
SKLX50940	JBAHUM000000000	CC59	ST59	IVa (2B)	t1151	2014
SKLX52512	JBAHMG000000000	CC59	ST59	IVa (2B)	t437	2014
SKLX60733	JBAGTS000000000	CC59	ST59	IVa (2B)	t437	2017
SKLX61393	JBAGWE000000000	CC59	ST59	V (5C2&5)	t437	2017
SKLX77782	JBAGZO000000000	CC59	ST59	V (5C2&5)	t437	2018
SKLX81115	JBAGUS000000000	CC59	ST59	IVa (2B)	t437	2018
SKLX87764	JBAHCS000000000	CC59	ST59	IVa (2B)	t441	2019
SKLX87779	JBAHDO000000000	CC59	ST59	IVa (2B)	t437	2019
SKLX87780	JBAHDL000000000	CC59	ST59	IVa (2B)	t437	2019
SKLX87799	JBAHPV000000000	CC59	ST59	IVa (2B)	t163	2019
SKLX87805	JBAHTU000000000	CC59	ST59	IVa (2B)	t437	2019
SKLX100269	JBAHKS000000000	CC59	ST59	IVa (2B)	t437	2019
SKLX100630	JBAHTW000000000	CC59	ST59	IVa (2B)	t441	2019
SKLX113866	JBAGXT000000000	CC59	ST59	IVa (2B)	t437	2019
SKLX81101	JBAGWB000000000	CC88	ST88	ΨSCC*mec*$_{ST88}$	t7637	2018
SKLX115112	JBAGWA000000000	CC59	ST59	IVa (2B)	t437	2019
SKLX82774	JBAHFU000000000	CC45	ST508	IVi (2B)	t015	2018
SKLX82790	JBAHGX000000000	CC59	ST338	V (5C2&5)	t437	2018

SKLX100707	JBAHGJ000000000	CC398	ST398	V (5C2)	t034	2019
SKLX103083	JBAHRH000000000	CC8	ST630	V (5C2)	t4549	2019
SKLX108304	JBAHRX000000000	CC121	ST121	V (5C2&5)	t9518	2019
SKLX113785	JBAGWV000000000	CC5	ST764	II (2A)	t1084	2019
SKLX115124	JBAHAH000000000	CC45	ST45	IVa (2B)	t073	2019
SKLX115129	JBAGXJ000000000	CC121	ST121	V (5C2&5)	t9518	2019
SKLX115137	JBAGVS000000000	CC88	ST88	V (5C2&5)	t17477	2019
SKLX115138	JBAGWN000000000	CC121	ST121	V (5C2&5)	t9518	2019
SKLX115141	JBAHQH000000000	CC7	ST7	IVh (2B)	t091	2019
SKLX116294	JBAHSQ000000000	CC8	ST239	III (3A)	t030	2019
SKLX52320	JBAHMF000000000	CC8	ST239	III (3A)	t030	2014
SKLX52339	JBAHNN000000000	CC8	ST239	III (3A)	t030	2014
SKLX52399	JBAHNG000000000	CC30	ST30	IVa (2B)	t2147	2014
SKLX56027	JBAGWO000000000	CC59	ST3355	IVa (2B)	t437	2015
SKLX56053	JBAHRI000000000	CC8	ST239	III (3A)	t030	2015
SKLX58971	JBAHQI000000000	CC8	ST239	III (3A)	t030	2016
SKLX59015	JBAHOZ000000000	CC8	ST239	III (3A)	t030	2016
SKLX59048	JBAHNP000000000	CC8	ST239	III (3A)	t030	2016
SKLX59977	JBAHBU000000000	CC8	ST239	III (3A)	t030	2016
SKLX59978	JBAHDD000000000	CC59	ST338	V (5C2&5)	t437	2016
SKLX59986	JBAHDC000000000	CC8	ST239	III (3A)	t030	2016
SKLX60011	JBAHBT000000000	CC8	ST239	III (3A)	t030	2016
SKLX60032	JBAHDV000000000	CC8	ST239	III (3A)	t030	2016
SKLX61006	JBAGXK000000000	CC8	ST239	III (3A)	t030	2017
SKLX61008	JBAGXW000000000	CC8	ST239	III (3A)	t030	2017
SKLX61654	JBAHJY000000000	CC8	ST239	III (3A)	t030	2017
SKLX53068	JBAHLV000000000	CC59	ST59	IVa (2B)	None	2015
SKLX54431	JBAHCG000000000	CC59	ST59	IVa (2B)	t437	2015
SKLX57323	JBAHFD000000000	CC59	ST59	IVa (2B)	t172	2016
SKLX57330	JBAHFN000000000	CC59	ST59	IVa (2B)	t441	2016
SKLX61010	JBAGUR000000000	CC59	ST59	IVa (2B)	t437	2017
SKLX83183	JBAHGC000000000	CC59	ST59	IVa (2B)	t437	2018
SKLX119012	JBAHBR000000000	CC59	ST59	V (5C2&5)	t441	2019
SKLX119015	JBAHAW000000000	CC59	ST59	IVa (2B)	t3523	2019
SKLX87900	JBAHPT000000000	CC59	ST8483	IVa (2B)	t437	2019
SKLX119013	JBAHBA000000000	CC8	ST239	III (3A)	t233	2019
SKLX60218	JBAHDE000000000	CC5	ST5	II (2A)	t2460	2017
SKLX50517	JBAHGV000000000	CC8	ST8470	III (3A)	t030	2014

SKLX53001	JBAHMP000000000	CC59	ST338	V (5C2&5)	t437	2015
SKLX54561	JBAGZR000000000	CC8	ST239	III (3A)	t632	2015
SKLX61977	JBAHIC000000000	CC59	ST8365	IVa (2B)	t17857	2017
SKLX62563	JBAHIJ000000000	CC8	ST239	III (3A)	t632	2017
SKLX63108	JBAHTQ000000000	CC8	ST630	V (5C2)	t4549	2018
SKLX53010	JBAHMX000000000	CC59	ST59	IVa (2B)	t437	2015
SKLX56367	JBAGUV000000000	CC59	ST59	IVa (2B)	t172	2016
SKLX59080	JBAHLC000000000	CC59	ST59	IVa (2B)	t437	2016
SKLX59644	JBAHAI000000000	CC59	ST59	IVg (2B)	t437	2016
SKLX59655	JBAHBI000000000	CC59	ST59	IVg (2B)	t437	2016
SKLX60185	JBAHAL000000000	CC59	ST59	IVa (2B)	t437	2017
SKLX60246	JBAHAA000000000	CC59	ST59	V (5C2&5)	t437	2017
SKLX60915	JBAGUF000000000	CC59	ST59	IVa (2B)	t163	2017
SKLX62574	JBAHRO000000000	CC59	ST59	IVa (2B)	t437	2017
SKLX63133	JBAHSL000000000	CC59	ST59	IVa (2B)	t3424	2018
SKLX78557	JBAGXO000000000	CC59	ST59	IVa (2B)	t437	2018
SKLX87214	JBAHEI000000000	CC59	ST3031	IVa (2B)	t14062	2018
SKLX79131	JBAGZK000000000	CC59	ST59	IVa (2B)	t437	2018
SKLX82063	JBAHFW000000000	CC59	ST59	IVa (2B)	t437	2018
SKLX83877	JBAHEU000000000	CC59	ST59	IVa (2B)	t172	2018
SKLX94343	JBAHNH000000000	CC59	ST59	V (5C2&5)	t437	2019
SKLX94352	JBAHMI000000000	CC59	ST59	V (5C2&5)	t437	2019
SKLX94709	JBAHNB000000000	CC59	ST59	IVa (2B)	t437	2019
SKLX100435	JBAGXI000000000	CC59	ST59	IVa (2B)	t437	2019
SKLX100469	JBAHPI000000000	CC59	ST59	IVa (2B)	t441	2019
SKLX100488	JBAHBW000000000	CC59	ST59	IVa (2B)	t437	2019
SKLX107050	JBAHLD000000000	CC59	ST59	IVa (2B)	t437	2019
SKLX108558	JBAHKR000000000	CC59	ST59	IVa (2B)	t437	2019
SKLX94718	JBAHBV000000000	CC8	ST72	IVc (2B)	t2431	2019
SKLX108580	JBAHJR000000000	CC59	ST59	IVa (2B)	t172	2019
SKLX108596	JBAHKD000000000	CC59	ST59	IVa (2B)	t172	2019
SKLX109558	JBAHJN000000000	CC59	ST59	IVa (2B)	t437	2019
SKLX109579	JBAHNC000000000	CC59	ST59	IVa (2B)	t437	2019
SKLX115846	JBAHOC000000000	CC59	ST59	IVa (2B)	t437	2019
SKLX115924	JBAHOW000000000	CC59	ST59	IVa (2B)	t437	2019
SKLX115928	JBAHSC000000000	CC59	ST59	IVa (2B)	t4145	2019
SKLX109490	JBAHIY000000000	CC8	ST72	IVc (2B)	t2431	2019
SKLX109556	JBAHOH000000000	CC59	ST7437	IVa (2B)	t437	2019

SKLX113971	JBAGXE000000000	CC59	ST338	V (5C2&5)	t3590	2019
SKLX115188	JBAHIE000000000	CC8	ST72	IVc (2B)	t2431	2019
SKLX115236	JBAHIF000000000	CC8	ST72	IVc (2B)	t2431	2019
SKLX115943	JBAHTB000000000	CC8	ST239	III (3A)	t037	2019
SKLX115984	JBAHPJ000000000	CC8	ST239	III (3A)	t037	2019
SKLX119044	JBAHEB000000000	CC22	ST22	V (5C2&5)	t309	2019
SKLX119055	JBAHCT000000000	CC22	ST22	V (5C2&5)	t309	2019
SKLX85152	JBAHEG000000000	CC59	ST59	IVa (2B)	t3517	2018
SKLX108240	JBAHDY000000000	CC5	ST5	II (2A)	t264	2019
SKLX95282	JBAHMQ000000000	CC398	ST398	V (5C2)	t034	2019
SKLX108216	JBAHED000000000	CC59	ST59	IVa (2B)	t441	2019
SKLX115712	JBAHSB000000000	CC59	ST59	IVa (2B)	t437	2019
SKLX108212	JBAHFT000000000	CC59	ST4513	IVa (2B)	t437	2019
SKLX89726	JBAHPP000000000	CC45	ST45	IVa (2B)	t073	2019
SKLX107813	JBAGRY000000000	CC59	ST338	V (5C2&5)	t437	2019
SKLX58095	JBAHRY000000000	CC8	ST239	III (3A)	t030	2016
SKLX58116	JBAHSH000000000	CC8	ST239	III (3A)	t030	2016
SKLX59234	JBAHOU000000000	CC8	ST239	III (3A)	t030	2016
SKLX59235	JBAHLG000000000	CC8	ST239	III (3A)	t030	2016
SKLX59236	JBAHLB000000000	CC8	ST239	III (3A)	t030	2016
SKLX59237	JBAHLL000000000	CC8	ST239	III (3A)	t030	2016
SKLX61436	JBAGVR000000000	CC59	ST8354	IVa (2B)	t437	2017
SKLX61437	JBAHIV000000000	CC59	ST8354	IVa (2B)	t437	2017
SKLX59279	JBAHKM000000000	CC59	ST59	IVa (2B)	t519	2016
SKLX59280	JBAHQY000000000	CC59	ST59	IVa (2B)	t519	2016
SKLX81100	JBAGVV000000000	CC59	ST59	IVa (2B)	t441	2018
SKLX94275	JBAHRA000000000	CC188	ST188	IVa (2B)	t189	2019
SKLX94276	JBAHMH000000000	CC59	ST338	V (5C2&5)	t437	2019
SKLX94289	JBAHOP000000000	CC59	ST338	V (5C2&5)	t437	2019
SKLX94291	JBAHPH000000000	CC188	ST188	IVa (2B)	t189	2019
SKLX79236	JBAGYC000000000	CC59	ST59	IVa (2B)	t437	2017
SKLX84007	JBAHDT000000000	CC59	ST59	IVa (2B)	t437	2018
SKLX16401	JBAHAU000000000	CC88	ST88	PF-SCC	t7637	2014
SKLX25060	JBAHBY000000000	CC8	ST630	V (5C2&5)	t4549	2014
SKLX25891	JBAHDS000000000	CC88	ST8349	ΨSCC*mec*$_{ST88}$	t7637	2014
SKLX27505	JBAGTL000000000	CC5	ST965	IVc (2B)	t062	2015
SKLX28037	JBAGTE000000000	CC398	ST398	V (5C2)	t034	2015
SKLX29848	JBAGTA000000000	CC8	ST239	III (3A)	t030	2015

SKLX15827	JBAHDK000000000	CC5	ST5	II (2A)	t311	2014
SKLX16270	JBAHBD000000000	CC5	ST5	II (2A)	t311	2014
SKLX31080	JBAGTN000000000	CC59	ST3193	IVa (2B)	t172	2015
SKLX16735	JBAHEP000000000	CC5	ST5	II (2A)	t311	2014
SKLX31883	JBAGVF000000000	CC8	ST630	V (5C2&5)	t4549	2015
SKLX32421	JBAGSO000000000	CC59	ST8370	IVa (2B)	t172	2015
SKLX17689	JBAHBJ000000000	CC5	ST5	II (2A)	t311	2014
SKLX33447	JBAGTI000000000	CC398	ST398	V (5C2)	t034	2015
SKLX18135	JBAHBL000000000	CC5	ST5	II (2A)	t311	2014
SKLX18162	JBAHBH000000000	CC5	ST5	II (2A)	t311	2014
SKLX18306	JBAHBO000000000	CC5	ST5	II (2A)	t311	2014
SKLX34951	JBAHHX000000000	CC398	ST398	V (5C2)	t034	2015
SKLX24210	JBAHCC000000000	CC5	ST5	II (2A)	t311	2014
SKLX24800	JBAHAP000000000	CC5	ST5	II (2A)	t311	2014
SKLX25737	JBAHEV000000000	CC5	ST5	II (2A)	t311	2014
SKLX36764	JBAHID000000000	CC59	ST338	V (5C2&5)	t13774	2016
SKLX26299	JBAHBK000000000	CC5	ST5	II (2A)	t002	2014
SKLX37716	JBAHHV000000000	CC398	ST398	V (5C2)	t034	2016
SKLX26732	JBAHAT000000000	CC5	ST5	II (2A)	t311	2014
SKLX27323	JBAGTP000000000	CC5	ST5	II (2A)	t311	2015
SKLX27748	JBAGXC000000000	CC5	ST5	II (2A)	t311	2015
SKLX28278	JBAGTR000000000	CC5	ST5	II (2A)	t311	2015
SKLX39166	JBAHHT000000000	CC8	ST239	III (3A)	t421	2016
SKLX28293	JBAGWC000000000	CC5	ST5	II (2A)	t311	2015
SKLX39449	JBAHHS000000000	CC5	ST764	II (2A)	t045	2016
SKLX28321	JBAGTU000000000	CC5	ST5	II (2A)	t311	2015
SKLX28547	JBAGSZ000000000	CC5	ST5	II (2A)	t311	2015
SKLX28717	JBAGTY000000000	CC5	ST5	II (2A)	t311	2015
SKLX28940	JBAGUW000000000	CC5	ST5	II (2A)	t002	2015
SKLX29954	JBAGTO000000000	CC5	ST5	II (2A)	t311	2015
SKLX43302	JBAHDG000000000	CC5	ST8486	II (2A)	t311	2016
SKLX43765	JBAHHL000000000	CC188	ST188	IVa (2B)	t3887	2016
SKLX30064	JBAGZB000000000	CC5	ST5	II (2A)	t311	2015
SKLX30455	JBAGTH000000000	CC5	ST5	II (2A)	t311	2015
SKLX30625	JBAGUC000000000	CC5	ST5	II (2A)	t311	2015
SKLX30908	JBAGUM000000000	CC5	ST5	II (2A)	t311	2015
SKLX31081	JBAGUJ000000000	CC5	ST5	II (2A)	t311	2015
SKLX48445	JBAHGK000000000	CC1	ST8469	IVa (2B)	t693	2017

SKLX32757	JBAGTC000000000	CC5	ST5	II (2A)	t3557	2015
SKLX49750	JBAHHR000000000	CC398	ST398	V (5C2)	t034	2017
SKLX33555	JBAGTF000000000	CC5	ST5	II (2A)	t311	2015
SKLX34055	JBAGSQ000000000	CC5	ST5	II (2A)	t311	2015
SKLX34646	JBAGTG000000000	CC5	ST5	II (2A)	t311	2015
SKLX35241	JBAHIK000000000	CC5	ST5	II (2A)	t311	2015
SKLX35874	JBAHIG000000000	CC5	ST5	II (2A)	t311	2015
SKLX36296	JBAHIQ000000000	CC5	ST5	II (2A)	t311	2016
SKLX37159	JBAHIA000000000	CC5	ST5	II (2A)	t311	2016
SKLX37808	JBAHGI000000000	CC5	ST5	II (2A)	t311	2016
SKLX37885	JBAHNR000000000	CC5	ST5	II (2A)	t311	2016
SKLX38043	JBAHHK000000000	CC5	ST5	II (2A)	t311	2016
SKLX38960	JBAHHC000000000	CC5	ST5	II (2A)	t311	2016
SKLX39359	JBAHKP000000000	CC5	ST5	II (2A)	t311	2016
SKLX51683	JBAHSM000000000	CC1	ST1	IVg (2B)	t114	2014
SKLX51707	JBAHSX000000000	CC5	ST965	IVc (2B)	t062	2014
SKLX39989	JBAHGZ000000000	CC5	ST5	II (2A)	t2731	2016
SKLX40085	JBAHHE000000000	CC5	ST5	II (2A)	t311	2016
SKLX40627	JBAHHM000000000	CC5	ST5	II (2A)	t311	2016
SKLX40823	JBAHHH000000000	CC5	ST5	II (2A)	t311	2016
SKLX41869	JBAHGE000000000	CC5	ST5	II (2A)	t311	2016
SKLX45729	JBAHDH000000000	CC5	ST5	II (2A)	t311	2017
SKLX46857	JBAHHA000000000	CC5	ST5	II (2A)	t311	2017
SKLX47505	JBAHDA000000000	CC5	ST5	II (2A)	t311	2017
SKLX47820	JBAHCQ000000000	CC5	ST5	II (2A)	t311	2017
SKLX47979	JBAHGM000000000	CC5	ST5	II (2A)	t311	2017
SKLX48519	JBAHCX000000000	CC5	ST5	II (2A)	t311	2017
SKLX53417	JBAHQT000000000	CC8	ST239	III (3A)	t037	2015
SKLX53430	JBAHPR000000000	CC5	ST8471	II (2A)	t002	2015
SKLX50248	JBAHFQ000000000	CC5	ST5	II (2A)	t311	2014
SKLX50258	JBAHGR000000000	CC5	ST5	II (2A)	t311	2014
SKLX50298	JBAHGW000000000	CC5	ST5	II (2A)	t311	2014
SKLX51312	JBAHUO000000000	CC5	ST5	II (2A)	t002	2014
SKLX51378	JBAHUE000000000	CC5	ST5	II (2A)	t311	2014
SKLX51389	JBAHUK000000000	CC5	ST5	II (2A)	t311	2014
SKLX51393	JBAHRU000000000	CC5	ST5	II (2A)	t311	2014
SKLX51395	JBAHTS000000000	CC5	ST5	II (2A)	t311	2014
SKLX51408	JBAHRS000000000	CC5	ST5	II (2A)	t311	2014

SKLX51654	JBAHUB000000000	CC5	ST5	II (2A)	t311	2014
SKLX55661	JBAHLO000000000	CC8	ST72	IVc (2B)	t2461	2015
SKLX51666	JBAHTP000000000	CC5	ST5	II (2A)	t311	2015
SKLX56262	JBAGSH000000000	CC1	ST1	IVa (2B)	t1908	2016
SKLX56266	JBAGTB000000000	CC5	ST8471	II (2A)	None	2016
SKLX56774	JBAHIN000000000	CC8	ST72	V (5C2&5)	t148	2016
SKLX56776	JBAHJG000000000	CC8	ST72	V (5C2&5)	t148	2016
SKLX51867	JBAHTY000000000	CC5	ST5	II (2A)	t311	2014
SKLX52838	JBAHNK000000000	CC5	ST5	II (2A)	t2460	2014
SKLX56854	JBAHIP000000000	CC8	ST72	V (5C2&5)	t4100	2016
SKLX52855	JBAHMT000000000	CC5	ST5	II (2A)	t2460	2014
SKLX58740	JBAHRP000000000	CC5	ST965	IVc (2B)	t062	2016
SKLX52922	JBAHNI000000000	CC5	ST5	II (2A)	t311	2015
SKLX58752	JBAHSK000000000	CC59	ST4513	IVa (2B)	t8886	2016
SKLX58763	JBAHSF000000000	CC5	ST965	IVc (2B)	t062	2016
SKLX53231	JBAHOV000000000	CC5	ST5	II (2A)	t311	2015
SKLX53288	JBAHMR000000000	CC5	ST5	II (2A)	t311	2015
SKLX53308	JBAHQP000000000	CC5	ST5	II (2A)	t002	2015
SKLX60107	JBAHAG000000000	CC5	ST965	IVc (2B)	t062	2016
SKLX60120	JBAHCV000000000	CC5	ST965	IVc (2B)	t062	2016
SKLX53342	JBAHND000000000	CC5	ST5	II (2A)	t311	2015
SKLX53357	JBAHMO000000000	CC5	ST5	II (2A)	t311	2015
SKLX60173	JBAHBS000000000	CC5	ST965	IVc (2B)	t062	2017
SKLX53388	JBAHNE000000000	CC5	ST5	II (2A)	t311	2015
SKLX60260	JBAHCP000000000	CC8	ST630	V (5C2)	t4549	2017
SKLX54114	JBAHAJ000000000	CC5	ST5	II (2A)	t311	2015
SKLX54236	JBAHAK000000000	CC5	ST5	II (2A)	t311	2015
SKLX54268	JBAGZJ000000000	CC5	ST5	II (2A)	t311	2015
SKLX61826	JBAHIW000000000	CC88	ST88	IVc (2B)	t2310	2017
SKLX62471	JBAHHZ000000000	CC9	ST9	XII (9C2)	t899	2017
SKLX55037	JBAGZT000000000	CC5	ST5	II (2A)	t002	2015
SKLX55182	JBAGYQ000000000	CC5	ST5	II (2A)	t311	2015
SKLX55190	JBAGXS000000000	CC5	ST5	II (2A)	t311	2015
SKLX55622	JBAGSJ000000000	CC5	ST5	II (2A)	t311	2016
SKLX64992	JBAHJO000000000	CC8	ST630	V (5C2&5)	t4549	2017
SKLX65073	JBAHMZ000000000	CC1	ST1	IVa (2B)	t127	2017
SKLX56841	JBAGRX000000000	CC5	ST5	II (2A)	t311	2016
SKLX56848	JBAGUZ000000000	CC5	ST5	II (2A)	t311	2016

SKLX56885	JBAGVP000000000	CC5	ST5	II (2A)	t311	2016
SKLX70159	JBAHKE000000000	CC398	ST398	V (5C2)	t034	2018
SKLX58741	JBAHSV000000000	CC5	ST5	II (2A)	t311	2016
SKLX70439	JBAHKA000000000	CC7	ST7	V (5C2&5)	t091	2018
SKLX70993	JBAHIX000000000	CC5	ST965	IVa (2B)	t062	2018
SKLX71502	JBAHKK000000000	CC5	ST8487	II (2A)	t3235	2018
SKLX58799	JBAHSG000000000	CC5	ST5	II (2A)	t311	2016
SKLX58815	JBAHSA000000000	CC5	ST5	II (2A)	t311	2016
SKLX74659	JBAHJH000000000	CC88	ST88	IVc (2B)	t15074	2018
SKLX60124	JBAHDW000000000	CC5	ST5	II (2A)	t311	2016
SKLX75869	JBAHIM000000000	CC398	ST398	V (5C2)	t011	2018
SKLX76788	JBAHLZ000000000	CC1	ST1	V (5C2)	t127	2018
SKLX60137	JBAHFC000000000	CC5	ST5	II (2A)	t311	2016
SKLX61063	JBAGRV000000000	CC5	ST5	II (2A)	t311	2017
SKLX61759	JBAHIB000000000	CC5	ST5	II (2A)	t311	2017
SKLX64029	JBAHPS000000000	CC5	ST5	II (2A)	t311	2017
SKLX68675	JBAHKG000000000	CC5	ST5	II (2A)	t311	2017
SKLX69629	JBAHIU000000000	CC5	ST5	II (2A)	t002	2018
SKLX69820	JBAHIS000000000	CC5	ST5	II (2A)	None	2018
SKLX70355	JBAHJP000000000	CC5	ST5	II (2A)	None	2018
SKLX72486	JBAHJJ000000000	CC5	ST5	II (2A)	t311	2018
SKLX72566	JBAHMW000000000	CC5	ST5	II (2A)	t311	2018
SKLX75802	JBAHKC000000000	CC5	ST5	II (2A)	t311	2018
SKLX79473	JBAGYA000000000	CC5	ST965	IVc (2B)	t062	2017
SKLX76890	JBAHAE000000000	CC5	ST5	II (2A)	t311	2018
SKLX79543	JBAGTX000000000	CC188	ST188	IVa (2B)	t189	2017
SKLX79556	JBAHCW000000000	CC8	ST72	V (5C2&5)	t148	2017
SKLX79593	JBAGTQ000000000	CC5	ST965	IVc (2B)	t062	2017
SKLX79611	JBAGUE000000000	CC1	ST1	IVg (2B)	t114	2017
SKLX77120	JBAGYT000000000	CC5	ST5	II (2A)	t311	2018
SKLX79700	JBAGSC000000000	CC5	ST965	IVa (2B)	t062	2018
SKLX78048	JBAGZY000000000	CC5	ST5	II (2A)	t2460	2018
SKLX79710	JBAGYE000000000	CC5	ST965	IVa (2B)	t062	2018
SKLX79729	JBAGYY000000000	CC5	ST965	IVc (2B)	t062	2018
SKLX79732	JBAGVG000000000	CC5	ST965	IVc (2B)	t062	2018
SKLX79370	JBAGXD000000000	CC5	ST5	II (2A)	t311	2017
SKLX79376	JBAGYL000000000	CC5	ST5	II (2A)	t311	2017
SKLX79752	JBAGVA000000000	CC5	ST965	IVc (2B)	t062	2018

SKLX79769	JBAGUY000000000	CC5	ST965	IVc (2B)	t062	2018
SKLX79793	JBAGVL000000000	CC5	ST965	IVc (2B)	t062	2018
SKLX80731	JBAGVN000000000	CC22	ST22	V (5C2&5)	t309	2018
SKLX80737	JBAGZI000000000	CC88	ST88	V (5C2)	t2526	2018
SKLX79391	JBAGXP000000000	CC5	ST5	II (2A)	t311	2017
SKLX79455	JBAGYG000000000	CC5	ST5	II (2A)	t311	2017
SKLX79475	JBAGSS000000000	CC5	ST5	II (2A)	t311	2017
SKLX81560	JBAHFM000000000	CC8	ST630	V (5C2)	t4549	2018
SKLX79687	JBAGTW000000000	CC5	ST5	II (2A)	t311	2018
SKLX83000	JBAHGG000000000	CC59	ST8367	IVa (2B)	t172	2018
SKLX79708	JBAGUO000000000	CC5	ST5	II (2A)	t311	2018
SKLX79735	JBAGVI000000000	CC5	ST5	II (2A)	t311	2018
SKLX79749	JBAGVK000000000	CC5	ST5	II (2A)	t311	2018
SKLX81207	JBAGVD000000000	CC5	ST5	II (2A)	t311	2018
SKLX84046	JBAHDX000000000	CC398	ST398	V (5C2)	t034	2018
SKLX81208	JBAHGB000000000	CC5	ST5	II (2A)	t1215	2018
SKLX81408	JBAHHD000000000	CC5	ST5	II (2A)	t1215	2018
SKLX81650	JBAHGT000000000	CC5	ST5	II (2A)	t1215	2018
SKLX87673	JBAHGQ000000000	CC398	ST398	V (5C2)	t1456	2019
SKLX83220	JBAHEQ000000000	CC5	ST5	II (2A)	t311	2018
SKLX88401	JBAHPK000000000	CC398	ST398	V (5C2)	t034	2019
SKLX84669	JBAHDM000000000	CC5	ST5	II (2A)	t311	2018
SKLX88790	JBAHQD000000000	CC5	ST965	IVc (2B)	t062	2019
SKLX89005	JBAHSO000000000	CC5	ST965	IVc (2B)	t062	2019
SKLX89268	JBAHPZ000000000	CC8	ST1821	V (5C2&5)	t4549	2019
SKLX89771	JBAHUG000000000	CC1	ST1	IVa (2B)	t127	2019
SKLX84976	JBAHDP000000000	CC5	ST5	II (2A)	t311	2018
SKLX88115	JBAHPM000000000	CC5	ST5	II (2A)	t002	2019
SKLX94422	JBAHMU000000000	CC5	ST965	IVa (2B)	t1399	2019
SKLX88750	JBAHOS000000000	CC5	ST5	II (2A)	t311	2019
SKLX91914	JBAHNL000000000	CC5	ST5	II (2A)	t311	2019
SKLX98172	JBAHMS000000000	CC88	ST88	IVc (2B)	t2310	2019
SKLX98176	JBAHNT000000000	CC59	ST8368	IVa (2B)	t437	2019
SKLX92185	JBAHOY000000000	CC5	ST5	II (2A)	t311	2019
SKLX99020	JBAHME000000000	CC398	ST1232	V (5C2&5)	t034	2019
SKLX101489	JBAHAZ000000000	CC22	ST22	V (5C2&5)	t309	2019
SKLX101499	JBAGSW000000000	CC1	ST1	Unknown	t127	2019
SKLX101709	JBAGYO000000000	CC59	ST8369	IVa (2B)	t437	2019

SKLX97039	JBAHOK000000000	CC5	ST5	II (2A)	t311	2019
SKLX98330	JBAHQR000000000	CC5	ST5	II (2A)	t002	2019
SKLX15777	JBAHBG000000000	CC59	ST59	IVa (2B)	t437	2014
SKLX26216	JBAHDZ000000000	CC59	ST59	IVa (2B)	t437	2014
SKLX107203	JBAHKW000000000	CC398	ST398	V (5C2)	t034	2019
SKLX107205	JBAHKL000000000	CC398	ST398	V (5C2)	t034	2019
SKLX28285	JBAGTM000000000	CC59	ST59	IVa (2B)	t437	2015
SKLX37344	JBAHGO000000000	CC59	ST59	IVa (2B)	t437	2016
SKLX37345	JBAHHY000000000	CC59	ST59	IVa (2B)	t437	2016
SKLX37803	JBAHHN000000000	CC59	ST59	IVa (2B)	t437	2016
SKLX39485	JBAHGP000000000	CC59	ST59	IVa (2B)	t437	2016
SKLX101727	JBAHHO000000000	CC5	ST5	II (2A)	t311	2019
SKLX41515	JBAHHF000000000	CC59	ST59	IVa (2B)	t163	2016
SKLX42090	JBAHDB000000000	CC59	ST59	IVa (2B)	t163	2016
SKLX45006	JBAHCU000000000	CC59	ST59	IVa (2B)	t437	2017
SKLX52851	JBAHNX000000000	CC59	ST59	IVa (2B)	t172	2014
SKLX52911	JBAHLQ000000000	CC59	ST59	IVa (2B)	t437	2015
SKLX53321	JBAHMD000000000	CC59	ST59	IVa (2B)	t3523	2015
SKLX55040	JBAGZG000000000	CC59	ST59	IVa (2B)	t437	2015
SKLX55046	JBAGZC000000000	CC59	ST59	IVa (2B)	t172	2015
SKLX55638	JBAGWZ000000000	CC59	ST59	IVa (2B)	t172	2015
SKLX57137	JBAHJV000000000	CC59	ST59	IVa (2B)	t163	2016
SKLX58137	JBAHSS000000000	CC59	ST59	IVa (2B)	t437	2016
SKLX59465	JBAHJX000000000	CC59	ST59	IVa (2B)	t172	2016
SKLX60157	JBAHCF000000000	CC59	ST59	IVa (2B)	t3523	2017
SKLX60180	JBAGZS000000000	CC59	ST59	IVa (2B)	t163	2017
SKLX65332	JBAHKN000000000	CC59	ST59	IVa (2B)	t437	2017
SKLX111090	JBAHFH000000000	CC22	ST22	IVh (2B)	t032	2019
SKLX101735	JBAHHU000000000	CC5	ST5	II (2A)	t311	2019
SKLX111114	JBAHEF000000000	CC5	ST5	II (2A)	t311	2019
SKLX67162	JBAHJM000000000	CC59	ST59	IVa (2B)	t437	2017
SKLX111448	JBAHDF000000000	CC5	ST5	II (2A)	t311	2019
SKLX68096	JBAHNU000000000	CC59	ST59	IVa (2B)	t172	2017
SKLX70335	JBAHJA000000000	CC59	ST59	IVa (2B)	t437	2018
SKLX111539	JBAHNJ000000000	CC188	ST188	IVa (2B)	t189	2019
SKLX111548	JBAHMJ000000000	CC188	ST188	IVa (2B)	t189	2019
SKLX111553	JBAHLX000000000	CC188	ST188	IVa (2B)	t189	2019
SKLX111515	JBAHPX000000000	CC5	ST5	II (2A)	t311	2019

SKLX111584	JBAHBX000000000	CC5	ST5	II (2A)	t311	2019
SKLX70821	JBAHIT000000000	CC59	ST59	IVa (2B)	t172	2018
SKLX112970	JBAHCI000000000	CC398	ST398	V (5C2)	t034	2019
SKLX113385	JBAHHP000000000	CC6	ST6	IVa (2B)	t304	2019
SKLX113386	JBAHBP000000000	CC6	ST6	IVa (2B)	t304	2019
SKLX79363	JBAGXZ000000000	CC59	ST59	IVa (2B)	t441	2017
SKLX112240	JBAHEX000000000	CC5	ST5	II (2A)	t2460	2019
SKLX114481	JBAGZX000000000	CC5	ST5	II (2A)	t311	2019
SKLX79371	JBAGXG000000000	CC59	ST59	V (5C2&5)	t437	2017
SKLX79477	JBAGTV000000000	CC59	ST59	IVa (2B)	t441	2017
SKLX79620	JBAGTK000000000	CC59	ST59	IVa (2B)	t172	2017
SKLX79682	JBAGRW000000000	CC59	ST59	IVa (2B)	t437	2018
SKLX114776	JBAGWL000000000	CC5	ST5	II (2A)	t002	2019
SKLX81729	JBAHJF000000000	CC59	ST59	IVa (2B)	t437	2018
SKLX82998	JBAHFS000000000	CC59	ST59	IVa (2B)	t437	2018
SKLX84856	JBAHHG000000000	CC59	ST59	IVa (2B)	t172	2018
SKLX85887	JBAHDR000000000	CC59	ST59	IVa (2B)	t437	2018
SKLX116023	JBAHOI000000000	CC5	ST5	IVc (2B)	t1560	2019
SKLX116038	JBAHQL000000000	CC5	ST965	IVc (2B)	t062	2019
SKLX90707	JBAHQG000000000	CC59	ST59	IVa (2B)	t437	2019
SKLX110014	JBAGXL000000000	CC59	ST59	IVa (2B)	t437	2019
SKLX111068	JBAHGN000000000	CC59	ST59	IVa (2B)	None	2019
SKLX111481	JBAHCR000000000	CC59	ST59	IVa (2B)	t172	2019
SKLX117362	JBAHNY000000000	CC188	ST188	IVa (2B)	t189	2019
SKLX111519	JBAHPD000000000	CC59	ST59	IVa (2B)	t172	2019
SKLX111531	JBAHMY000000000	CC59	ST59	IVa (2B)	t172	2019
SKLX112632	JBAHCK000000000	CC59	ST59	IVa (2B)	t16634	2019
SKLX114966	JBAGYK000000000	CC59	ST59	IVa (2B)	t6596	2019
SKLX114986	JBAGWH000000000	CC59	ST59	IVa (2B)	None	2019
SKLX113430	JAWSPP000000000	CC59	ST338	V (5C2&5)	t437	2020
SKLX114810	JAWSPO000000000	CC5	ST5	ΨSCC*mec* (ST5)	t002	2020
SKLX116729	JAWSPN000000000	CC5	ST8488	II (2A)	t311	2020
SKLX116893	JAWSPM000000000	CC5	ST5	II (2A)	t311	2020
SKLX117601	JAWSPL000000000	CC5	ST5	II (2A)	t311	2020
SKLX118273	JAWSPK000000000	CC59	ST59	IVa (2B)	t437	2020
SKLX119106	JAWSPJ000000000	CC8	ST239	III (3A)	t030	2020
SKLX119149	JAWSPI000000000	CC59	ST59	IVa (2B)	t437	2020
SKLX119166	JAWSPH000000000	CC8	ST630	V (5C2&5)	t4549	2020

SKLX119288	JAWSPG000000000	CC59	ST59	IVa (2B)	t1950	2020
SKLX119895	JAWSPF000000000	CC59	ST59	IVa (2B)	t172	2020
SKLX119944	JAWSPE000000000	CC5	ST5	II (2A)	t2460	2020
SKLX119951	JAWSPD000000000	CC398	ST398	V (5C2)	t034	2020
SKLX119957	JAWSPC000000000	CC59	ST59	IVa (2B)	t7501	2020
SKLX120038	JAWSPB000000000	CC59	ST59	V (5C2&5)	t437	2020
SKLX120162	JAWSPA000000000	CC8	ST72	IVc (2B)	t2461	2020
SKLX120164	JAWSOZ000000000	CC59	ST59	IVa (2B)	t441	2020
SKLX120267	JAWSOY000000000	CC88	ST88	IVa (2B)	t186	2020
SKLX120288	JAWSOX000000000	CC59	ST8467	V (5C2&5)	t437	2020
SKLX120366	JAWSOW000000000	CC59	ST59	IVa (2B)	t437	2020
SKLX120456	JAWSOV000000000	CC5	ST5	II (2A)	t2460	2020
SKLX120472	JAWSOU000000000	CC398	ST1232	V (5C2&5)	t034	2020
SKLX120518	JAWSOT000000000	CC8	ST239	III (3A)	t030	2020
SKLX120610	JAWSOS000000000	CC59	ST59	IVa (2B)	t437	2020
SKLX120704	JAWSOR000000000	CC88	ST88	ΨSCC*mec*$_{\text{ST88}}$	t12147	2020
SKLX120758	JAWSOQ000000000	CC188	ST188	IVa (2B)	t189	2020
SKLX120997	JAWSOP000000000	CC59	ST59	IVa (2B)	t529	2020
SKLX121005	JAWSOO000000000	CC59	ST59	IVa (2B)	t529	2020
SKLX121006	JAWSON000000000	CC59	ST59	IVa (2B)	t172	2020
SKLX121013	JAWSOM000000000	CC59	ST59	V (5C2&5)	t437	2020
SKLX121107	JAWSOL000000000	CC59	ST59	V (5C2&5)	t437	2020
SKLX121128	JAWSOK000000000	CC22	ST22	V (5C2&5)	t309	2020
SKLX121448	JAWSOJ000000000	CC1	ST1	IVa (2B)	t127	2020
SKLX121727	JAWSOI000000000	CC398	ST398	V (5C2)	t034	2020
SKLX121730	JAWSOH000000000	CC398	ST398	V (5C2)	t034	2020
SKLX121731	JAWSOG000000000	CC398	ST398	V (5C2)	t034	2020
SKLX121746	JAWSOF000000000	CC59	ST59	IVg (2B)	t1751	2020
SKLX121917	JAWSOE000000000	CC5	ST5	II (2A)	t2724	2020
SKLX122190	JAWSOD000000000	CC59	ST338	V (5C2&5)	t437	2020
SKLX122208	JAWSOC000000000	CC59	ST59	IVa (2B)	t437	2020
SKLX122232	JAWSOB000000000	CC8	ST72	V (5C2&5)	t4100	2020
SKLX122249	JAWSOA000000000	CC398	ST398	V (5C2)	t034	2020
SKLX122277	JAWSNZ000000000	CC398	ST398	V (5C2)	t034	2020
SKLX122951	JAWSNY000000000	CC8	ST239	III (3A)	t030	2020
SKLX122955	JAWSNX000000000	CC59	ST59	IVa (2B)	t437	2020
SKLX123887	JAWSNW000000000	CC88	ST88	IVc (2B)	t10450	2020
SKLX125232	JAWSNV000000000	CC8	ST630	V (5C2&5)	t4549	2020

SKLX125235	JAWSNU000000000	CC398	ST398	V (5C2)	t034	2020
SKLX125274	JAWSNT000000000	CC5	ST5	II (2A)	t311	2020
SKLX125368	JAWSNS000000000	CC59	ST59	IVg (2B)	t437	2020
SKLX125401	JAWSNR000000000	CC8	ST72	IVc (2B)	t2461	2020
SKLX125403	JAWSNQ000000000	CC8	ST72	IVc (2B)	t2461	2020
SKLX125558	JAWSNP000000000	CC59	ST59	IVa (2B)	t437	2020
SKLX125835	JAWSNO000000000	CC59	ST59	IVg (2B)	t437	2020
SKLX125861	JAWSNN000000000	CC398	ST398	V (5C2)	t034	2020
SKLX125907	JAWSNM000000000	CC88	ST88	ΨSCC*mec*$_{ST88}$	t7637	2020
SKLX125934	JAWSNL000000000	CC398	ST398	V (5C2)	t034	2020
SKLX125955	JAWSNK000000000	CC59	ST59	V (5C2&5)	t437	2020
SKLX125978	JAWSNJ000000000	CC59	ST8351	IVa (2B)	t437	2020
SKLX126113	JAWSNI000000000	CC59	ST59	IVa (2B)	t437	2020
SKLX126129	JAWSNH000000000	CC45	ST45	IVa (2B)	t073	2020
SKLX126171	JAWSNG000000000	CC59	ST59	IVa (2B)	None	2020
SKLX126189	JAWSNF000000000	CC5	ST5	II (2A)	t5076	2020
SKLX126194	JAWSNE000000000	CC5	ST5	II (2A)	t5076	2020
SKLX126221	JAWSND000000000	CC6	ST6	IVa (2B)	t304	2020
SKLX126248	JAWSNC000000000	CC8	ST239	III (3A)	t421	2020
SKLX126254	JAWSNB000000000	CC8	ST239	III (3A)	t421	2020
SKLX126255	JAWSNA000000000	CC8	ST239	III (3A)	t421	2020
SKLX126605	JAWSMZ000000000	CC5	ST5	II (2A)	t311	2020
SKLX126615	JAWSMY000000000	CC5	ST5	II (2A)	t311	2020
SKLX126652	JAWSMX000000000	CC5	ST965	IVc (2B)	t062	2020
SKLX126733	JAWSMW000000000	CC59	ST59	IVa (2B)	t437	2020
SKLX126737	JAWSMV000000000	CC59	ST59	V (5C2&5)	t437	2020
SKLX126793	JAWSMU000000000	CC59	ST59	IVg (2B)	t13954	2020
SKLX126896	JAWSMT000000000	CC5	ST5	II (2A)	t311	2020
SKLX127157	JAWSMS000000000	CC59	ST59	IVa (2B)	t437	2020
SKLX127262	JAWSMR000000000	CC88	ST88	ΨSCC*mec*$_{ST88}$	t7637	2020
SKLX127618	JAWSMQ000000000	CC59	ST59	IVa (2B)	t437	2020
SKLX127642	JAWSMP000000000	CC59	ST59	V (5C2&5)	t437	2020
SKLX127656	JAWSMO000000000	CC59	ST59	IVa (2B)	t437	2020
SKLX127658	JAWSMN000000000	CC59	ST59	IVa (2B)	t437	2020
SKLX127675	JAWSMM000000000	CC8	ST72	IVc (2B)	t2431	2020
SKLX127686	JAWSML000000000	CC59	ST59	IVa (2B)	t437	2020
SKLX127723	JAWSMK000000000	CC398	ST398	V (5C2)	t1255	2020
SKLX127743	JAWSMJ000000000	CC88	ST88	ΨSCC*mec*$_{ST88}$	t7637	2020

SKLX127756	JAWSMI000000000	CC59	ST59	IVa (2B)	t437	2020
SKLX127856	JAWSMH000000000	CC59	ST59	IVa (2B)	t437	2020
SKLX127879	JAWSMG000000000	CC8	ST72	IVc (2B)	t4359	2020
SKLX127917	JAWSMF000000000	CC59	ST59	IVa (2B)	t172	2020
SKLX127943	JAWSME000000000	CC59	ST59	IVa (2B)	t437	2020
SKLX127978	JAWSMD000000000	CC59	ST59	IVa (2B)	t437	2020
SKLX128017	JAWSMC000000000	CC5	ST5	II (2A)	t2460	2020
SKLX128112	JAWSMB000000000	CC30	ST2580	IV* (2B)	t3351	2020
SKLX128122	JAWSMA000000000	CC59	ST3193	IVa (2B)	t172	2020
SKLX128170	JAWSLZ000000000	CC188	ST188	IVa (2B)	t189	2020
SKLX128199	JAWSLY000000000	CC8	ST239	III (3A)	t030	2020
SKLX128216	JAWSLX000000000	CC59	ST59	IVa (2B)	t437	2020
SKLX128277	JAWSLW000000000	CC59	ST59	IVa (2B)	t437	2020
SKLX128895	JAWSLV000000000	CC5	ST5	II (2A)	t088	2020
SKLX129039	JAWSLU000000000	CC5	ST5	II (2A)	t311	2020
SKLX129725	JAWSLT000000000	CC59	ST59	IVa (2B)	t172	2020
SKLX130045	JAWSLS000000000	CC5	ST5	ΨSCC*mec* (ST5)	t002	2020
SKLX130563	JAWSLR000000000	CC59	ST8352	IVa (2B)	t441	2020
SKLX131166	JAWSLQ000000000	CC6	ST6	IVa (2B)	t304	2020
SKLX131759	JAWSLP000000000	CC22	ST22	V (5C2&5)	t309	2020
SKLX132278	JAWSLO000000000	CC22	ST22	IVa (2B)	t5763	2020
SKLX132472	JAWSLN000000000	CC59	ST4513	IVa (2B)	t529	2020
SKLX132718	JAWSLM000000000	CC59	ST59	IVa (2B)	t437	2020
SKLX133567	JAWSLL000000000	CC59	ST8356	V (5C2&5)	t437	2020
SKLX133570	JAWSLK000000000	CC88	ST88	ΨSCCmec_{ST88}	t5351	2020
SKLX133610	JAWSLJ000000000	CC398	ST398	V (5C2)	t034	2020
SKLX133650	JAWSLI000000000	CC59	ST59	IVa (2B)	None	2020
SKLX133655	JAWSLH000000000	CC59	ST59	IVa (2B)	t437	2020
SKLX133669	JAWSLG000000000	CC59	ST59	IVa (2B)	t437	2020
SKLX133714	JAWSLF000000000	CC59	ST59	IVa (2B)	t11911	2020
SKLX133737	JAWSLE000000000	CC398	ST398	V (5C2&5)	t034	2020
SKLX133738	JAWSLD000000000	CC59	ST8360	IVa (2B)	t437	2020
SKLX133770	JAWSLC000000000	CC45	ST8468	IVa (2B)	t776	2020
SKLX133829	JAWSLB000000000	CC398	ST398	V (5C2&5)	t034	2020
SKLX133850	JAWSLA000000000	CC59	ST8353	IVa (2B)	t441	2020
SKLX133887	JAWSKZ000000000	CC8	ST72	V (5C2&5)	t148	2020
SKLX133905	JAWSKY000000000	CC59	ST59	IVa (2B)	t437	2020
SKLX134057	JAWSKX000000000	CC8	ST72	IVc (2B)	t148	2020

SKLX134547	JAWSKW000000000	CC398	ST398	V (5C2)	t011	2020
SKLX134572	JAWSKV000000000	CC59	ST59	IVa (2B)	t437	2020
SKLX134590	JAWSKU000000000	CC88	ST88	ΨSCC*mec*$_{ST88}$	t7637	2020
SKLX134624	JAWSKT000000000	CC8	ST630	V (5C2&5)	t4549	2020
SKLX134630	JAWSKS000000000	CC6	ST6	IVa (2B)	t304	2020
SKLX134712	JAWSKR000000000	CC5	ST5	II (2A)	t311	2020
SKLX134766	JAWSKQ000000000	CC5	ST5	II (2A)	t311	2020
SKLX134901	JAWSKP000000000	CC59	ST3355	IVa (2B)	t437	2020
SKLX134988	JAWSKO000000000	CC88	ST88	ΨSCC*mec*$_{ST88}$	None	2020
SKLX135083	JAWSKN000000000	CC6	ST6	IVa (2B)	t304	2020
SKLX135100	JAWSKM000000000	CC59	ST59	IVa (2B)	t437	2020
SKLX135114	JAWSKL000000000	CC5	ST5	V (5C2&5)	t311	2020
SKLX135120	JAWSKK000000000	CC5	ST5	II (2A)	t311	2020
SKLX135145	JAWSKJ000000000	CC59	ST8363	V (5C2&5)	t437	2020
SKLX135170	JAWSKI000000000	CC59	ST59	IVa (2B)	t172	2020
SKLX135246	JAWSKH000000000	CC5	ST5	II (2A)	t311	2020
SKLX136046	JAWSKG000000000	CC59	ST59	IVa (2B)	t437	2020
SKLX136047	JAWSKF000000000	CC59	ST59	IV* (2B)	t3590	2020
SKLX136163	JAWSKE000000000	CC59	ST59	IVa (2B)	t437	2020
SKLX136199	JAWSKD000000000	CC8	ST72	IVc (2B)	t324	2020
SKLX136234	JAWSKC000000000	CC59	ST59	IVa (2B)	t437	2020
SKLX136256	JAWSKB000000000	CC22	ST22	V (5C2&5)	t1328	2020
SKLX136276	JAWSKA000000000	CC59	ST59	IVa (2B)	t437	2020
SKLX136309	JAWSJZ000000000	CC8	ST239	III (3A)	t030	2020
SKLX136327	JAWSJY000000000	CC59	ST59	V (5C2&5)	t437	2020
SKLX136345	JAWSJX000000000	CC59	ST59	IVa (2B)	t437	2020
SKLX136374	JAWSJW000000000	CC59	ST59	IVa (2B)	t437	2020
SKLX136406	JAWSJV000000000	CC8	ST72	IVc (2B)	t324	2020
SKLX136596	JAWSJU000000000	CC398	ST398	V (5C2)	t034	2020
SKLX140542	JAWSJT000000000	CC398	ST398	V (5C2)	t034	2020
SKLX140543	JAWSJS000000000	CC59	ST3193	IVa (2B)	t172	2020
SKLX140554	JAWSJR000000000	CC59	ST59	IVa (2B)	t437	2020
SKLX140758	JAWSJQ000000000	CC22	ST22	V (5C2&5)	t309	2020
SKLX140804	JAWSJP000000000	CC59	ST59	IVa (2B)	t172	2020
SKLX141282	JAWSJO000000000	CC59	ST8361	IVa (2B)	t437	2020
SKLX141541	JAWSJN000000000	CC6	ST6	IVa (2B)	t304	2020
SKLX141542	JAWSJM000000000	CC59	ST59	IVa (2B)	t441	2020
SKLX141543	JAWSJL000000000	CC59	ST59	IVa (2B)	t437	2020

SKLX141546	JAWSJK000000000	CC59	ST59	IVa (2B)	t4145	2020
SKLX141547	JAWSJJ000000000	CC59	ST59	IVa (2B)	t8347	2020
SKLX142904	JAWSJI000000000	CC5	ST5	II (2A)	t311	2020
SKLX144524	JAWSJH000000000	CC5	ST5	II (2A)	t2460	2020
SKLX144544	JAWSJG000000000	CC5	ST5	V (5C2)	t002	2020
SKLX144553	JAWSJF000000000	CC5	ST5	V (5C2)	t002	2020
SKLX144571	JAWSJE000000000	CC59	ST3355	IVa (2B)	t437	2020
SKLX144662	JAWSJD000000000	CC59	ST59	IVa (2B)	t437	2020
SKLX144664	JAWSJC000000000	CC88	ST4083	IVc (2B)	t2310	2020
SKLX144763	JAWSJB000000000	CC59	ST59	IVa (2B)	t172	2020
SKLX144797	JAWSJA000000000	CC8	ST239	III (3A)	t030	2020
SKLX144846	JAWSIZ000000000	CC5	ST5	II (2A)	t311	2020
SKLX144851	JAWSIY000000000	CC5	ST5	II (2A)	t311	2020
SKLX144877	JAWSIX000000000	CC59	ST59	IVa (2B)	t2755	2020
SKLX144917	JAWSIW000000000	CC59	ST8358	V (5C2&5)	t3590	2020
SKLX144978	JAWSIV000000000	CC398	ST398	V (5C2)	t034	2020
SKLX144984	JAWSIU000000000	CC398	ST398	V (5C2)	t034	2020
SKLX144988	JAWSIT000000000	CC398	ST398	V (5C2)	t034	2020
SKLX145068	JAWSIS000000000	CC398	ST398	V (5C2)	t034	2020
SKLX145082	JAWSIR000000000	CC398	ST398	V (5C2)	t011	2020
SKLX145187	JAWSIQ000000000	CC59	ST59	IVa (2B)	t441	2020
SKLX145637	JAWSIP000000000	CC59	ST59	IVa (2B)	t441	2020
SKLX145672	JAWSIO000000000	CC5	ST5	II (2A)	t2460	2020
SKLX145674	JAWSIN000000000	CC5	ST5	II (2A)	t2460	2020
SKLX145703	JAWSIM000000000	CC59	ST59	IVa (2B)	t437	2020
SKLX145720	JAWSIL000000000	CC22	ST22	V (5C2&5)	t309	2020
SKLX145765	JAWSIK000000000	CC398	ST8052	V (5C2)	t4652	2020
SKLX145811	JAWSIJ000000000	CC59	ST338	V (5C2&5)	t437	2020
SKLX145852	JAWSII000000000	CC398	ST8052	V (5C2)	t034	2020
SKLX145874	JAWSIH000000000	CC59	ST59	IVa (2B)	t441	2020
SKLX146788	JAWSIG000000000	CC59	ST8359	IVa (2B)	t172	2020
SKLX146895	JAWSIF000000000	CC5	ST5	II (2A)	t2460	2020
SKLX147040	JAWSIE000000000	CC5	ST5	IVc (2B)	t548	2020
SKLX147534	JAWSID000000000	CC5	ST965	IVc (2B)	t062	2020
SKLX147563	JAWSIC000000000	CC398	ST398	V (5C2)	t034	2020
SKLX152932	JAWSIB000000000	CC59	ST59	IVa (2B)	t441	2020
SKLX38194	JAWSIA000000000	CC5	ST5	II (2A)	t311	2016
SKLX38222	JAWSHZ000000000	CC59	ST338	V (5C2&5)	t437	2016

SKLX51130	JAWSHY000000000	CC8	ST239	III (3A)	t2270	2014
SKLX51186	JAWSHX000000000	CC59	ST59	IVa (2B)	t441	2014
SKLX51532	JAWSHW000000000	CC59	ST59	IVa (2B)	t441	2014
SKLX51656	JAWSHV000000000	CC5	ST5	II (2A)	t311	2014
SKLX51969	JAWSHU000000000	CC59	ST3186	IVa (2B)	t437	2014
SKLX52065	JAWSHT000000000	CC8	ST72	IVc (2B)	t2431	2014
SKLX52118	JAWSHS000000000	CC59	ST59	IVa (2B)	t12011	2014
SKLX52178	JAWSHR000000000	CC59	ST59	IVa (2B)	t12011	2014
SKLX53756	JAWSHQ000000000	CC398	ST8472	V (5C2)	t034	2015
SKLX53869	JAWSHP000000000	CC8	ST239	III (3A)	t030	2015
SKLX56575	JAWSHO000000000	CC121	ST121	V (5C2&5)	t159	2016
SKLX56589	JAWSHN000000000	CC59	ST59	IVa (2B)	t437	2016
SKLX56610	JAWSHM000000000	CC59	ST59	IVg (2B)	t3523	2016
SKLX56892	JAWSHL000000000	CC5	ST5	II (2A)	t311	2016
SKLX57159	JAWSHK000000000	CC59	ST59	IVa (2B)	t163	2016
SKLX57440	JAWSHJ000000000	CC8	ST239	III (3A)	t037	2016
SKLX57445	JAWSHI000000000	CC398	ST398	V (5C2)	t011	2016
SKLX57662	JAWSHH000000000	CC59	ST59	IVa (2B)	t3385	2016
SKLX57881	JAWSHG000000000	CC398	ST398	V (5C2)	t011	2016
SKLX58685	JAWSHF000000000	CC1	ST8476	IVa (2B)	None	2016
SKLX58822	JAWSHE000000000	CC5	ST5	II (2A)	t311	2016
SKLX59060	JAWSHD000000000	CC59	ST59	IVa (2B)	t437	2016
SKLX59492	JAWSHC000000000	CC59	ST59	IVg (2B)	t437	2020
SKLX59563	JAWSHB000000000	CC59	ST59	IVa (2B)	t437	2020
SKLX59883	JAWSHA000000000	CC398	ST8478	V (5C2)	t034	2017
SKLX60069	JAWSGZ000000000	CC5	ST5	II (2A)	t311	2017
SKLX60076	JAWSGY000000000	CC5	ST5	II (2A)	t311	2017
SKLX60808	JAWSGX000000000	CC8	ST239	III (3A)	t030	2017
SKLX60826	JAWSGW000000000	CC8	ST239	III (3A)	t030	2017
SKLX60838	JAWSGV000000000	CC8	ST239	III (3A)	t030	2017
SKLX60842	JAWSGU000000000	CC8	ST239	III (3A)	t030	2017
SKLX61324	JAWSGT000000000	CC59	ST59	IVa (2B)	t437	2020
SKLX62689	JAWSGS000000000	CC398	ST398	V (5C2)	t1456	2018
SKLX62839	JAWSGR000000000	CC59	ST59	IVa (2B)	t437	2018
SKLX63914	JAWSGQ000000000	CC398	ST398	V (5C2)	t011	2018
SKLX63985	JAWSGP000000000	CC59	ST59	IVa (2B)	t437	2018
SKLX67669	JAWSGO000000000	CC5	ST5	II (2A)	t311	2017
SKLX69581	JAWSGN000000000	CC5	ST5	II (2A)	t002	2018

SKLX76951	JAWSGM000000000	CC59	ST59	IVa (2B)	t441	2018
SKLX81770	JAWSGL000000000	CC59	ST59	IVa (2B)	t3590	2018
SKLX83041	JAWSGK000000000	CC398	ST398	V (5C2)	t011	2018
SKLX83690	JAWSGJ000000000	CC398	ST398	V (5C2)	t1793	2018
SKLX83695	JAWSGI000000000	CC5	ST764	II (2A)	t002	2018
SKLX87222	JAWSGH000000000	CC59	ST59	IVa (2B)	t437	2018
SKLX87246	JAWSGG000000000	CC59	ST59	IVa (2B)	t437	2018
SKLX87825	JAWSGF000000000	CC59	ST59	IVa (2B)	t437	2019
SKLX88344	JAWSGE000000000	CC59	ST59	IVa (2B)	t437	2019
SKLX88607	JAWSGD000000000	CC59	ST59	IVa (2B)	t437	2019
SKLX88659	JAWSGC000000000	CC8	ST239	III (3A)	t030	2019
SKLX89394	JAWSGB000000000	CC59	ST3193	IVa (2B)	t172	2019
SKLX89437	JAWSGA000000000	CC5	ST5	II (2A)	t2460	2019
SKLX89450	JAWSFZ000000000	CC59	ST59	IVa (2B)	t437	2019
SKLX89537	JAWSFY000000000	CC22	ST22	V (5C2&5)	t3668	2019
SKLX90375	JAWSFX000000000	CC5	ST5	II (2A)	t311	2019
SKLX91027	JAWSFW000000000	CC59	ST59	IVa (2B)	t163	2019
SKLX93019	JAWSFV000000000	CC398	ST398	V (5C2)	t034	2019
SKLX94337	JAWSFU000000000	CC59	ST59	IVa (2B)	t172	2019
SKLX100712	JAWSFT000000000	CC59	ST59	IVa (2B)	t437	2019
SKLX113500	JAWSFS000000000	CC59	ST338	V (5C2&5)	t437	2020
SKLX117249	JAWSFR000000000	CC6	ST6	IVa (2B)	t304	2020
SKLX119939	JAWSFQ000000000	CC1	ST1	IVg (2B)	t114	2020
SKLX119941	JAWSFP000000000	CC22	ST22	V (5C2&5)	t309	2020
SKLX121710	JAWSFO000000000	CC8	ST630	V (5C2)	t4549	2020
SKLX121718	JAWSFN000000000	CC22	ST22	V (5C2&5)	t309	2020
SKLX122275	JAWSFM000000000	CC5	ST965	IVc (2B)	t640	2020
SKLX125947	JAWSFL000000000	CC22	ST22	V (5C2&5)	t309	2020
SKLX125985	JAWSFK000000000	CC8	ST630	V (5C2&5)	t4549	2020
SKLX126471	JAWSFJ000000000	CC22	ST22	V (5C2&5)	t309	2020
SKLX126510	JAWSFI000000000	CC22	ST22	V (5C2&5)	t309	2020
SKLX127807	JAWSFH000000000	CC22	ST22	V (5C2&5)	t309	2020
SKLX127898	JAWSFG000000000	CC59	ST59	IVa (2B)	t437	2020
SKLX133695	JAWSFF000000000	CC59	ST59	IVa (2B)	t437	2020
SKLX134118	JAWSFE000000000	CC5	ST8206	IVc (2B)	t062	2020
SKLX134146	JAWSFD000000000	CC45	ST45	IVa (2B)	t2195	2020
SKLX134149	JAWSFC000000000	CC45	ST45	IVa (2B)	t2195	2020
SKLX136618	JAWSFB000000000	CC59	ST59	IVa (2B)	t3527	2020

SKLX140756	JAWSFA000000000	CC1	ST1	IVg (2B)	t114	2020
SKLX141893	JAWSEZ000000000	CC8	ST630	V (5C2&5)	t4549	2020
SKLX142180	JAWSEY000000000	CC22	ST22	V (5C2&5)	t309	2020
SKLX142565	JAWSEX000000000	CC59	ST338	V (5C2&5)	t437	2020
SKLX143757	JAWSEW000000000	CC22	ST22	V (5C2&5)	t309	2020
SKLX144244	JAWSEV000000000	CC22	ST22	V (5C2&5)	t309	2020
SKLX144510	JAWSEU000000000	CC59	ST338	V (5C2&5)	t3590	2020
SKLX144562	JAWSET000000000	CC59	ST338	V (5C2&5)	t437	2020
SKLX144792	JAWSES000000000	CC22	ST22	V (5C2&5)	t309	2020
SKLX144858	JAWSER000000000	CC59	ST59	V (5C2&5)	t437	2020
SKLX146971	JAWSEQ000000000	CC59	ST59	V (5C2&5)	t437	2020
SKLX146993	JAWSEP000000000	CC59	ST59	IVa (2B)	t437	2020
SKLX147740	JAWSEO000000000	CC5	ST5	IV* (2B)	t13727	2020
SKLX44852	JAWSEN000000000	CC59	ST59	IVa (2B)	t437	2020
SKLX45995	JAWSEM000000000	CC5	ST965	IVc (2B)	t062	2017
SKLX59511	JAWSEL000000000	CC59	ST59	IVg (2B)	t437	2017
SKLX59684	JAWSEK000000000	CC59	ST59	IVa (2B)	t437	2017
SKLX60414	JAWSEJ000000000	CC5	ST5	II (2A)	t2460	2017
SKLX60517	JAWSEI000000000	CC59	ST59	IVa (2B)	t437	2017
SKLX60525	JAWSEH000000000	CC59	ST59	IVa (2B)	t437	2017
SKLX60528	JAWSEG000000000	CC59	ST59	IVa (2B)	t437	2017
SKLX61872	JAWSEF000000000	CC398	ST398	V (5C2)	t034	2017
SKLX61899	JAWSEE000000000	CC8	ST630	V (5C2)	t4549	2017
SKLX62077	JAWSED000000000	CC59	ST59	IVa (2B)	t437	2017
SKLX62269	JAWSEC000000000	CC5	ST5529	IVg (2B)	t688	2017
SKLX63396	JAWSEB000000000	CC5	ST5	II (2A)	t2460	2018
SKLX63510	JAWSEA000000000	CC59	ST59	V (5C2&5)	t437	2018
SKLX67792	JAWSDZ000000000	CC398	ST398	V (5C2)	t034	2017
SKLX78558	JAWSDY000000000	CC22	ST22	V (5C2&5)	t309	2018
SKLX85187	JAWSDX000000000	CC8	ST72	IVc (2B)	t6509	2018
SKLX85701	JAWSDW000000000	CC45	ST45	IVa (2B)	t21048	2018
SKLX85720	JAWSDV000000000	CC59	ST3260	V (5C2&5)	t437	2018
SKLX88828	JAWSDU000000000	CC22	ST22	V (5C2&5)	t309	2019
SKLX88923	JAWSDT000000000	CC59	ST59	IVa (2B)	t437	2019
SKLX96378	JAWSDS000000000	CC59	ST59	V (5C2&5)	t437	2019
SKLX97549	JAWSDR000000000	CC59	ST338	V (5C2&5)	t437	2019
SKLX97567	JAWSDQ000000000	CC398	ST398	V (5C2)	t034	2019
SKLX97576	JAWSDP000000000	CC398	ST8275	V (5C2)	t898	2019

SKLX97578	JAWSDO000000000	CC398	ST398	V (5C2)	t034	2019
SKLX97589	JAWSDN000000000	CC398	ST398	V (5C2)	t4652	2019
SKLX4953	JAWSDM000000000	CC8	ST239	III (3A)	None	2014
SKLX6805	JAWSDL000000000	CC8	ST239	III (3A)	t037	2014
SKLX7554	JAWSDK000000000	CC8	ST239	III (3A)	t030	2014
SKLX16424	JAWSDJ000000000	CC8	ST239	III (3A)	t030	2014
SKLX17572	JAWSDI000000000	CC8	ST239	III (3A)	t030	2014
SKLX18423	JAWSDH000000000	CC8	ST239	III (3A)	t030	2014
SKLX25599	JAWSDG000000000	CC8	ST239	III (3A)	t030	2014
SKLX26147	JAWSDF000000000	CC8	ST239	III (3A)	t2270	2014
SKLX27116	JAWSDE000000000	CC8	ST239	III (3A)	t030	2014
SKLX27530	JAWSDD000000000	CC8	ST239	III (3A)	t030	2015
SKLX29571	JAWSDC000000000	CC8	ST239	III (3A)	t030	2015
SKLX32885	JAWSDB000000000	CC8	ST239	III (3A)	t030	2015
SKLX36547	JAWSDA000000000	CC8	ST239	III (3A)	t030	2016
SKLX41399	JAWSCZ000000000	CC8	ST239	III (3A)	t030	2016
SKLX42156	JAWSCY000000000	CC8	ST239	III (3A)	t037	2016
SKLX43239	JAWSCX000000000	CC8	ST239	III (3A)	t037	2016
SKLX44458	JAWSCW000000000	CC8	ST239	III (3A)	t037	2017
SKLX99159	JAWSCV000000000	CC8	ST239	III (3A)	t030	2019
SKLX1381	JAWSCU000000000	CC59	ST59	IVa (2B)	t441	2011
SKLX1890	JAWSCT000000000	CC59	ST59	IVa (2B)	t3736	2011
SKLX1940	JAWSCS000000000	CC59	ST59	V (5C2&5)	t437	2011
SKLX20173	JAWSCR000000000	CC8	ST239	III (3A)	t030	2011
SKLX20174	JAWSCQ000000000	CC8	ST239	III (3A)	t030	2011
SKLX20175	JAWSCP000000000	CC8	ST239	III (3A)	t030	2011
SKLX20176	JAWSCO000000000	CC8	ST239	III (3A)	t030	2011
SKLX20177	JAWSCN000000000	CC8	ST239	III (3A)	t030	2011
SKLX20178	JAWSCM000000000	CC8	ST239	III (3A)	t037	2011
SKLX20179	JAWSCL000000000	CC8	ST239	III (3A)	t030	2011
SKLX20180	JAWSCK000000000	CC8	ST239	III (3A)	t030	2012
SKLX20183	JAWSCJ000000000	CC8	ST239	III (3A)	t030	2012
SKLX20400	JAWSCI000000000	CC59	ST59	IVa (2B)	t437	2012
SKLX20401	JAWSCH000000000	CC5	ST5	II (2A)	t002	2012
SKLX20402	JAWSCG000000000	CC59	ST59	V (5C2&5)	t437	2012
SKLX20403	JAWSCF000000000	CC5	ST5	II (2A)	t311	2012
SKLX20404	JAWSCE000000000	CC5	ST5	II (2A)	t311	2012
SKLX20407	JAWSCD000000000	CC5	ST5	II (2A)	t002	2012

SKLX20410	JAWSCC000000000	CC59	ST59	IVa (2B)	t437	2012
SKLX20411	JAWSCB000000000	CC5	ST5	II (2A)	t311	2012
SKLX20412	JAWSCA000000000	CC5	ST5	II (2A)	t311	2012
SKLX20413	JAWSBZ000000000	CC8	ST239	III (3A)	t030	2012
SKLX20416	JAWSBY000000000	CC1	ST8519	IVa (2B)	t127	2012
SKLX20420	JAWSBX000000000	CC5	ST5	II (2A)	t311	2012
SKLX20421	JAWSBW000000000	CC5	ST5	II (2A)	t311	2012
SKLX20422	JAWSBV000000000	CC8	ST239	III (3A)	t037	2012
SKLX20423	JAWSBU000000000	CC5	ST5	II (2A)	t002	2012
SKLX20425	JAWSBT000000000	CC5	ST5	II (2A)	t311	2012
SKLX20426	JAWSBS000000000	CC5	ST5	II (2A)	t311	2012
SKLX20434	JAWSBR000000000	CC59	ST59	V (5C2&5)	t437	2012
SKLX20436	JAWSBQ000000000	CC8	ST239	III (3A)	t030	2011
SKLX20437	JAWSBP000000000	CC5	ST5	II (2A)	t311	2011
SKLX20440	JAWSBO000000000	CC5	ST5	II (2A)	t311	2011
SKLX20580	JAWSBN000000000	CC8	ST239	III (3A)	t421	2012
SKLX20582	JAWSBM000000000	CC59	ST59	IVa (2B)	t437	2012
SKLX20585	JAWSBL000000000	CC59	ST59	IVa (2B)	t437	2011
SKLX20586	JAWSBK000000000	CC8	ST239	III (3A)	t030	2012
SKLX20591	JAWSBJ000000000	CC59	ST59	IVa (2B)	t437	2011
SKLX20599	JAWSBI000000000	CC8	ST239	III (3A)	t421	2012
SKLX20600	JAWSBH000000000	CC5	ST5	II (2A)	t002	2011
SKLX20601	JAWSBG000000000	CC59	ST59	V (5C2&5)	t437	2011
SKLX20604	JAWSBF000000000	CC8	ST8470	III (3A)	t030	2011
SKLX20609	JAWSBE000000000	CC8	ST239	III (3A)	t030	2011
SKLX20610	JAWSBD000000000	CC5	ST5	II (2A)	t002	2012
SKLX20611	JAWSBC000000000	CC59	ST8520	IVa (2B)	t437	2012
SKLX20613	JAWSBB000000000	CC8	ST239	III (3A)	t030	2011
SKLX20616	JAWSBA000000000	CC59	ST59	V (5C2&5)	t1894	2012
SKLX20618	JAWSAZ000000000	CC8	ST8470	III (3A)	t030	2012
SKLX20621	JAWSAY000000000	CC59	ST59	V (5C2&5)	t1894	2012
SKLX20624	JAWSAX000000000	CC8	ST8470	III (3A)	t030	2012
SKLX20836	JAWSAW000000000	CC8	ST8521	III (3A)	t037	2011
SKLX20837	JAWSAV000000000	CC45	ST45	IVa (2B)	t116	2011
SKLX20848	JAWSAU000000000	CC8	ST239	III (3A)	t074	2012
SKLX20853	JAWSAT000000000	CC59	ST59	IVa (2B)	t441	2012
SKLX20854	JAWSAS000000000	CC8	ST239	III (3A)	t030	2012
SKLX20855	JAWSAR000000000	CC8	ST239	III (3A)	t030	2012

SKLX20857	JAWSAQ000000000	CC59	ST59	V (5C2&5)	t437	2012
SKLX2104	JAWSAP000000000	CC8	ST239	III (3A)	t030	2011
SKLX2113	JAWSAO000000000	CC59	ST59	IVa (2B)	t437	2011
SKLX21328	JAWSAN000000000	CC8	ST239	III (3A)	t030	2011
SKLX21331	JAWSAM000000000	CC8	ST239	III (3A)	t030	2011
SKLX21332	JAWSAL000000000	CC8	ST239	III (3A)	t030	2011
SKLX21333	JAWSAK000000000	CC8	ST239	III (3A)	t030	2011
SKLX21336	JAWSAJ000000000	CC8	ST239	III (3A)	t030	2011
SKLX21337	JAWSAI000000000	CC8	ST239	III (3A)	t030	2011
SKLX21338	JAWSAH000000000	CC8	ST239	III (3A)	t030	2011
SKLX21339	JAWSAG000000000	CC8	ST239	III (3A)	t030	2011
SKLX21344	JAWSTF000000000	CC8	ST239	III (3A)	t030	2011
SKLX21345	JAWSTE000000000	CC8	ST239	III (3A)	t030	2011
SKLX21346	JAWSTD000000000	CC8	ST239	III (3A)	t030	2011
SKLX21351	JAWSTC000000000	CC8	ST239	III (3A)	t030	2011
SKLX21352	JAWSTB000000000	CC8	ST239	III (3A)	t030	2012
SKLX21353	JAWSTA000000000	CC8	ST239	III (3A)	t030	2012
SKLX21354	JAWSSZ000000000	CC8	ST239	III (3A)	t030	2012
SKLX21358	JAWSSY000000000	CC8	ST239	III (3A)	t030	2012
SKLX21359	JAWSSX000000000	CC8	ST239	III (3A)	t030	2012
SKLX21361	JAWSSW000000000	CC8	ST239	III (3A)	t030	2012
SKLX21362	JAWSSV000000000	CC8	ST239	III (3A)	t030	2012
SKLX21363	JAWSSU000000000	CC8	ST239	III (3A)	t030	2012
SKLX21364	JAWSST000000000	CC8	ST239	III (3A)	t030	2012
SKLX21365	JAWSSS000000000	CC8	ST239	III (3A)	t030	2012
SKLX21367	JAWSSR000000000	CC8	ST239	III (3A)	t030	2012
SKLX21368	JAWSSQ000000000	CC8	ST239	III (3A)	t030	2012
SKLX21371	JAWSSP000000000	CC8	ST239	III (3A)	t030	2012
SKLX21373	JAWSSO000000000	CC8	ST239	III (3A)	t030	2012
SKLX21374	JAWSSN000000000	CC8	ST239	III (3A)	t030	2012
SKLX21615	JAWSSM000000000	CC8	ST239	III (3A)	t030	2011
SKLX21616	JAWSSL000000000	CC8	ST239	III (3A)	t030	2011
SKLX21617	JAWSSK000000000	CC8	ST239	III (3A)	t030	2011
SKLX21619	JAWSSJ000000000	CC9	ST9	Unknown	t693	2011
SKLX21620	JAWSSI000000000	CC59	ST3355	IVa (2B)	t437	2011
SKLX21621	JAWSSH000000000	CC8	ST239	III (3A)	t030	2011
SKLX21622	JAWSSG000000000	CC8	ST239	III (3A)	t030	2011
SKLX21623	JAWSSF000000000	CC8	ST239	III (3A)	t030	2011

SKLX21624	JAWSSE000000000	CC59	ST59	IVa (2B)	t3590	2011
SKLX21625	JAWSSD000000000	CC8	ST239	III (3A)	t030	2011
SKLX21626	JAWSSC000000000	CC8	ST239	III (3A)	t030	2011
SKLX21628	JAWSSB000000000	CC8	ST239	III (3A)	t030	2011
SKLX21630	JAWSSA000000000	CC8	ST239	III (3A)	t030	2011
SKLX21631	JAWSRZ000000000	CC8	ST239	III (3A)	t030	2011
SKLX21632	JAWSRY000000000	CC8	ST239	III (3A)	t030	2011
SKLX21633	JAWSRX000000000	CC8	ST239	III (3A)	t030	2011
SKLX21635	JAWSRW000000000	CC59	ST3355	IVa (2B)	t437	2011
SKLX21636	JAWSRV000000000	CC8	ST239	III (3A)	t037	2011
SKLX21637	JAWSRU000000000	CC8	ST239	III (3A)	t030	2011
SKLX21638	JAWSRT000000000	CC8	ST239	III (3A)	t030	2011
SKLX21640	JAWSRS000000000	CC8	ST239	III (3A)	t030	2011
SKLX21641	JAWSRR000000000	CC59	ST59	IVa (2B)	t437	2011
SKLX21642	JAWSRQ000000000	CC8	ST239	III (3A)	t030	2011
SKLX21643	JAWSRP000000000	CC8	ST239	III (3A)	t030	2011
SKLX21644	JAWSRO000000000	CC8	ST239	III (3A)	t030	2011
SKLX21645	JAWSRN000000000	CC59	ST59	IVa (2B)	t437	2011
SKLX21647	JAWSRM000000000	CC8	ST239	III (3A)	t459	2011
SKLX21648	JAWSRL000000000	CC8	ST239	III (3A)	t030	2011
SKLX21650	JAWSRK000000000	CC8	ST239	III (3A)	t030	2011
SKLX21656	JAWSRJ000000000	CC8	ST239	III (3A)	t037	2011
SKLX21658	JAWSRI000000000	CC8	ST239	III (3A)	t030	2012
SKLX21659	JAWSRH000000000	CC8	ST239	III (3A)	t030	2012
SKLX21660	JAWSRG000000000	CC8	ST239	III (3A)	t030	2012
SKLX21662	JAWSRF000000000	CC8	ST239	III (3A)	t030	2012
SKLX21663	JAWSRE000000000	CC8	ST239	III (3A)	t030	2012
SKLX21664	JAWSRD000000000	CC8	ST239	III (3A)	t030	2012
SKLX21665	JAWSRC000000000	CC8	ST239	III (3A)	t030	2012
SKLX21666	JAWSRB000000000	CC59	ST59	IVa (2B)	t437	2012
SKLX21667	JAWSRA000000000	CC8	ST239	III (3A)	t030	2012
SKLX21668	JAWSQZ000000000	CC8	ST239	III (3A)	t030	2012
SKLX21669	JAWSQY000000000	CC8	ST239	III (3A)	t030	2012
SKLX21844	JAWSQX000000000	CC5	ST5	II (2A)	t311	2011
SKLX21845	JAWSQW000000000	CC59	ST3195	IVa (2B)	t437	2011
SKLX21849	JAWSQV000000000	CC5	ST5	II (2A)	t311	2011
SKLX21850	JAWSQU000000000	CC5	ST5	II (2A)	t311	2011
SKLX21853	JAWSQT000000000	CC5	ST5	II (2A)	t311	2011

SKLX21854	JAWSQS000000000	CC5	ST5	II (2A)	t002	2011
SKLX21857	JAWSQR000000000	CC5	ST5	II (2A)	t311	2011
SKLX21858	JAWSQQ000000000	CC5	ST5	II (2A)	t002	2011
SKLX21865	JAWSQP000000000	CC59	ST59	IVa (2B)	t437	2011
SKLX21866	JAWSQO000000000	CC5	ST5	II (2A)	t311	2011
SKLX21868	JAWSQN000000000	CC5	ST5	II (2A)	t311	2011
SKLX21869	JAWSQM000000000	CC59	ST59	V (5C2&5)	t437	2011
SKLX21870	JAWSQL000000000	CC8	ST239	III (3A)	t030	2011
SKLX21871	JAWSQK000000000	CC5	ST5	II (2A)	t311	2011
SKLX21872	JAWSQJ000000000	CC5	ST5	II (2A)	t311	2011
SKLX21874	JAWSQI000000000	CC5	ST5	II (2A)	t002	2011
SKLX21875	JAWSQH000000000	CC5	ST5	II (2A)	t311	2011
SKLX21876	JAWSQG000000000	CC8	ST239	III (3A)	t030	2012
SKLX21877	JAWSQF000000000	CC59	ST59	IVa (2B)	t437	2012
SKLX21878	JAWSQE000000000	CC8	ST239	III (3A)	t030	2012
SKLX21885	JAWSQD000000000	CC59	ST59	IVa (2B)	t437	2012
SKLX21886	JAWSQC000000000	CC8	ST72	IVc (2B)	t324	2012
SKLX21985	JAWSQB000000000	CC8	ST239	III (3A)	t030	2012
SKLX21986	JAWSQA000000000	CC8	ST1821	V (5C2)	t5554	2011
SKLX21989	JAWSPZ000000000	CC8	ST72	IVc (2B)	t324	2011
SKLX21990	JAWSPY000000000	CC5	ST5	II (2A)	t002	2012
SKLX21991	JAWSPX000000000	CC8	ST239	III (3A)	t030	2012
SKLX2686	JAWSPW000000000	CC8	ST239	III (3A)	t030	2011
SKLX3351	JAWSPV000000000	CC8	ST239	III (3A)	t030	2011
SKLX3357	JAWSPU000000000	CC8	ST239	III (3A)	t030	2011
SKLX3375	JAWSPT000000000	CC8	ST239	III (3A)	t030	2011
SKLX3994	JAWSPS000000000	CC8	ST239	III (3A)	t030	2011
SKLX20395	JAWSPR000000000	CC8	ST239	III (3A)	t037	2011
SKLX20595	JAWSPQ000000000	CC8	ST239	III (3A)	t037	2011
SKLX3282	JAWSTG000000000	CC8	ST239	III (3A)	t037	2011

Table S2. Comparison of antimicrobial susceptibilities between ST239 and ST59.

	ST239		ST59	
	n	%	n	%
Total	184		401	
Erythromycin	173	94.02	355	88.53
Clindamycin	117	63.59	344	85.79
Trimethoprim-sulfamethoxazole	25	13.59	0	0
Tetracycline	179	97.28	149	37.16
Ciprofloxacin	183	99.46	61	15.21
Levofloxacin	177	96.2	36	8.98
Moxifloxacin	178	96.74	36	8.98
Gentamicin	179	97.28	20	4.99
Amikacin	165	89.67	32	7.98
Rifampicin	166	90.22	7	1.75
Vancomycin	0	0	0	0
Tigecycline	4	2.17	2	0.5
Daptomycin	0	0	0	0
Linezolid	0	0	0	0

Table S3. Metadata of MRSA strains used in phenotypic and genetic assays.

Strain	ST	Clade	SCC*mec*	*spa*	*lukS-PV*	*lukF-PV*	*seb*	*chp*	*selk*	*selq*	*hla*	*sraP* gene length(bp)	immune-evasion cluster (IEC)	Province in China	Collection date	Associated experiment(s)*
SKLX144763	ST59	ST59-I	IVa (2B)	t172	0	0	1	1	1	1	1	5415	A (*sea-sak-chp-scn*)	Shandong	2020	Comparative fitness assay; stress-phenotype assay; cytotoxicity assay; animal infection assay
SKLX55046	ST59	ST59-I	IVa (2B)	t172	0	0	1	1	1	1	1	5415	A (*sea-sak-chp-scn*)	Zhejiang	2015	Comparative fitness assay; stress-phenotype assay; cytotoxicity assay; animal infection assay
SKLX84856	ST59	ST59-I	IVa (2B)	t172	0	0	1	1	1	1	1	5414	A (*sea-sak-chp-scn*)	Zhejiang	2018	Comparative fitness assay; stress-phenotype assay; cytotoxicity assay; animal infection assay
SKLX94337	ST59	ST59-I	IVa (2B)	t172	0	0	1	1	1	1	1	5415	A (*sea-sak-chp-scn*)	Shandong	2019	Comparative fitness assay; stress-phenotype assay; cytotoxicity assay; animal infection assay
SKLX145874	ST59	ST59-I	IVa (2B)	t441	0	0	0	1	0	0	1	5415	A (*sea-sak-chp-scn*)	Hubei	2020	Cytotoxicity assay; animal infection assay
SKLX42090	ST59	ST59-I	IVa (2B)	t163	0	0	1	1	1	1	1	5415	A (*sea-sak-chp-scn*)	Zhejiang	2016	Cytotoxicity assay; animal infection assay
SKLX50577	ST59	ST59-I	IVa (2B)	t163	0	0	0	1	0	0	1	5415	A (*sea-sak-chp-scn*)	Anhui	2014	Cytotoxicity assay; animal infection assay
SKLX53534	ST59	ST59-I	IVa (2B)	t172	0	0	1	1	1	1	1	5415	A (*sea-sak-chp-scn*)	Jiangsu	2015	Cytotoxicity assay; animal infection assay
SKLX57137	ST59	ST59-I	IVa (2B)	t163	0	0	1	1	1	1	1	5415	A (*sea-sak-chp-scn*)	Zhejiang	2016	Cytotoxicity assay; animal infection assay
SKLX68096	ST59	ST59-I	IVa (2B)	t172	0	0	1	1	1	1	1	5415	A (*sea-sak-chp-scn*)	Zhejiang	2017	Cytotoxicity assay; animal infection assay
SKLX117483	ST59	ST59-II	IVg (2B)	t441	0	0	1	0	1	1	1	6801	G (*selp-sak-scn*)	Fujian	2019	Comparative fitness assay; stress-phenotype assay; cytotoxicity assay; animal infection assay
SKLX126793	ST59	ST59-II	IVg (2B)	t13954	0	0	1	1	1	1	1	6801	B (*sak-chp-scn*)	Henan	2020	Comparative fitness assay; stress-phenotype assay; cytotoxicity assay; animal infection assay
SKLX57651	ST59	ST59-II	IVg (2B)	t437	0	0	1	0	1	1	1	6801	E (*sak-scn*)	Fujian	2016	Comparative fitness assay; stress-phenotype assay; cytotoxicity assay; animal infection assay
SKLX97572	ST59	ST59-II	IVg (2B)	t437	0	0	1	0	1	1	1	6801	G (*selp-sak-scn*)	Anhui	2019	Comparative fitness assay; stress-phenotype assay; cytotoxicity assay; animal infection assay
SKLX121746	ST59	ST59-II	IVg (2B)	t1751	0	0	1	1	1	1	1	6801	B (*sak-chp-scn*)	Fujian	2020	Cytotoxicity assay; animal infection assay
SKLX125835	ST59	ST59-II	IVg (2B)	t437	0	0	1	0	1	1	1	6801	G (*selp-sak-scn*)	Zhejiang	2020	Cytotoxicity assay; animal infection assay

SKLX54743	ST59	ST59-II	IVg (2B)	t1751	0	0	1	0	1	1	1	6801	G (*selp-sak-scn*)	Fujian	2015	Cytotoxicity assay; animal infection assay
SKLX56588	ST59	ST59-II	IVg (2B)	t1751	0	0	1	0	1	1	1	6801	G (*selp-sak-scn*)	Fujian	2016	Cytotoxicity assay; animal infection assay
SKLX59492	ST59	ST59-II	IVg (2B)	t437	0	0	1	1	1	1	1	6801	C (*chp-scn*)	Fujian	2020	Cytotoxicity assay; animal infection assay
SKLX59644	ST59	ST59-II	IVg (2B)	t437	0	0	1	0	1	1	1	6801	G (*selp-sak-scn*)	Shandong	2016	Cytotoxicity assay; animal infection assay
SKLX58137	ST59	ST59-III	IVa (2B)	t437	1	1	1	1	1	1	1	6759	C (*chp-scn*)	Zhejiang	2016	Comparative fitness assay; stress-phenotype assay; cytotoxicity assay; animal infection assay
SKLX65332	ST59	ST59-III	IVa (2B)	t437	1	1	1	1	1	1	1	6759	C (*chp-scn*)	Zhejiang	2017	Comparative fitness assay; stress-phenotype assay; cytotoxicity assay; animal infection assay
SKLX83809	ST59	ST59-III	IVa (2B)	t437	1	1	1	1	1	1	1	6759	C (*chp-scn*)	Anhui	2018	Comparative fitness assay; stress-phenotype assay; cytotoxicity assay; animal infection assay
SKLX88607	ST59	ST59-III	IVa (2B)	t437	1	1	1	1	1	1	1	6759	B (*sak-chp-scn*)	Anhui	2019	Comparative fitness assay; stress-phenotype assay; cytotoxicity assay; animal infection assay
SKLX11027 6	ST59	ST59-III	IVa (2B)	t437	1	1	0	1	0	0	1	6801	C (*chp-scn*)	Hubei	2019	Cytotoxicity assay; animal infection assay
SKLX12536 8	ST59	ST59-III	IVg (2B)	t437	0	0	0	1	0	0	1	6801	A (*sea-sak-chp-scn*)	Jiangxi	2020	Cytotoxicity assay; animal infection assay
SKLX13661 8	ST59	ST59-III	IVa (2B)	t3527	0	0	1	1	1	1	1	6801	C (*chp-scn*)	Hubei	2020	Cytotoxicity assay; animal infection assay
SKLX45006	ST59	ST59-III	IVa (2B)	t437	1	1	0	1	0	0	1	6759	C (*chp-scn*)	Zhejiang	2017	Cytotoxicity assay; animal infection assay
SKLX56579	ST59	ST59-III	IVa (2B)	t3424	1	1	1	1	1	1	1	6801	C (*chp-scn*)	Fujian	2016	Cytotoxicity assay; animal infection assay
SKLX83818	ST59	ST59-III	IVa (2B)	t437	1	1	1	1	1	1	1	6801	C (*chp-scn*)	Anhui	2018	Cytotoxicity assay; animal infection assay
SKLX12595 5	ST59	ST59-IV	V (5C2&5)	t437	1	1	1	1	1	1	1	6801	C (*chp-scn*)	Jiangxi	2020	Comparative fitness assay; stress-phenotype assay; cytotoxicity assay; animal infection assay
SKLX61303	ST59	ST59-IV	V (5C2&5)	t437	1	1	1	1	1	1	1	6705	C (*chp-scn*)	Anhui	2017	Comparative fitness assay; stress-phenotype assay; cytotoxicity assay; animal infection assay
SKLX63908	ST59	ST59-IV	V (5C2&5)	t437	1	1	1	1	1	1	1	6801	C (*chp-scn*)	Anhui	2018	Comparative fitness assay; stress-phenotype assay; cytotoxicity assay; animal infection assay
SKLX77782	ST59	ST59-IV	V (5C2&5)	t437	1	1	1	1	1	1	1	6801	C (*chp-scn*)	Jiangxi	2018	Comparative fitness assay; stress-phenotype assay; cytotoxicity assay; animal infection assay
SKLX20601	ST59	ST59-IV	V (5C2&5)	t437	1	1	1	1	1	1	1	6801	C (*chp-scn*)	Shandong	2011	Cytotoxicity assay; animal infection assay

SKLX53479	ST59	ST59-IV	V (5C2&5)	t437	1	1	0	1	0	0	1	6801	C (*chp-scn*)	Anhui	2015	Cytotoxicity assay; animal infection assay
SKLX58004	ST59	ST59-IV	V (5C2&5)	t437	1	1	1	1	1	1	1	6801	C (*chp-scn*)	Anhui	2016	Cytotoxicity assay; animal infection assay
SKLX60246	ST59	ST59-IV	V (5C2&5)	t437	1	1	0	1	0	0	1	6801	C (*chp-scn*)	Shandong	2017	Cytotoxicity assay; animal infection assay
SKLX61393	ST59	ST59-IV	V (5C2&5)	t437	1	1	1	1	1	1	1	6801	C (*chp-scn*)	Jiangxi	2017	Cytotoxicity assay; animal infection assay
SKLX96378	ST59	ST59-IV	V (5C2&5)	t437	0	0	0	1	0	0	1	6801	C (*chp-scn*)	Hubei	2019	Cytotoxicity assay; animal infection assay
SKLX15777	ST59	ST59-V	IVa (2B)	t437	0	0	1	1	1	1	1	6801	B (*sak-chp-scn*)	Zhejiang	2014	Comparative fitness assay; stress-phenotype assay; cytotoxicity assay; animal infection assay; mutant construction and complementation assay; transcriptomic profiling (pH 4.5 vs. pH 7.4)
SKLX39485	ST59	ST59-V	IVa (2B)	t437	0	0	1	1	1	1	1	6801	B (*sak-chp-scn*)	Zhejiang	2016	Comparative fitness assay; stress-phenotype assay; cytotoxicity assay; animal infection assay
SKLX52911	ST59	ST59-V	IVa (2B)	t437	0	0	1	1	1	1	1	6801	B (*sak-chp-scn*)	Zhejiang	2015	Comparative fitness assay; stress-phenotype assay; cytotoxicity assay; animal infection assay
SKLX85887	ST59	ST59-V	IVa (2B)	t437	0	0	1	1	1	1	1	6801	B (*sak-chp-scn*)	Zhejiang	2018	Comparative fitness assay; stress-phenotype assay; cytotoxicity assay; animal infection assay
SKLX127618	ST59	ST59-V	IVa (2B)	t437	0	0	0	1	0	0	1	6801	B (*sak-chp-scn*)	Shandong	2020	Cytotoxicity assay; animal infection assay
SKLX141543	ST59	ST59-V	IVa (2B)	t437	0	0	0	1	0	0	1	6801	B (*sak-chp-scn*)	Henan	2020	Cytotoxicity assay; animal infection assay
SKLX50788	ST59	ST59-V	IVa (2B)	t441	0	0	1	0	1	1	1	6801	E (*sak-scn*)	Anhui	2014	Cytotoxicity assay; animal infection assay
SKLX54720	ST59	ST59-V	IVa (2B)	t437	1	1	1	1	1	1	1	6801	B (*sak-chp-scn*)	Guangdong	2015	Cytotoxicity assay; animal infection assay
SKLX57230	ST59	ST59-V	IVa (2B)	t437	1	1	1	1	1	1	1	6801	B (*sak-chp-scn*)	Anhui	2016	Cytotoxicity assay; animal infection assay
SKLX59684	ST59	ST59-V	IVa (2B)	t437	0	0	0	1	0	0	1	6801	B (*sak-chp-scn*)	Gansu	2017	Cytotoxicity assay; animal infection assay
SKLX115943	ST239	ST239-I	III (3A)	t037	0	0	0	0	0	0	1	5280	D (*sea-sak-scn*)	Shandong	2019	Comparative fitness assay; stress-phenotype assay; cytotoxicity assay; animal infection assay
SKLX126248	ST239	ST239-I	III (3A)	t421	0	0	0	0	1	1	1	5280	D (*sea-sak-scn*)	Jiangsu	2020	Comparative fitness assay; stress-phenotype assay; cytotoxicity assay; animal infection assay
SKLX39166	ST239	ST239-I	III (3A)	t421	0	0	0	0	1	1	0	5280	D (*sea-sak-scn*)	Zhejiang	2016	Comparative fitness assay; stress-phenotype assay; cytotoxicity assay; animal infection assay

SKLX42156	ST239	ST239-I	III (3A)	t037	0	0	0	0	1	1	1	5280	D (*sea-sak-scn*)	Zhejiang	2016	Comparative fitness assay; stress-phenotype assay; cytotoxicity assay; animal infection assay
SKLX44458	ST239	ST239-I	III (3A)	t037	0	0	0	0	1	1	1	5280	D (*sea-sak-scn*)	Zhejiang	2017	Comparative fitness assay; stress-phenotype assay; cytotoxicity assay; animal infection assay
SKLX53417	ST239	ST239-I	III (3A)	t037	0	0	0	0	0	0	1	5280	Negative	Zhejiang	2015	Comparative fitness assay; stress-phenotype assay; cytotoxicity assay; animal infection assay
SKLX54652	ST239	ST239-I	III (3A)	t037	0	0	0	0	0	0	1	5280	D (*sea-sak-scn*)	Jiangsu	2015	Comparative fitness assay; stress-phenotype assay; cytotoxicity assay; animal infection assay
SKLX56296	ST239	ST239-I	III (3A)	t037	0	0	0	0	0	0	1	5280	D (*sea-sak-scn*)	Jiangsu	2016	Comparative fitness assay; stress-phenotype assay; cytotoxicity assay; animal infection assay
SKLX57440	ST239	ST239-I	III (3A)	t037	0	0	0	0	1	1	1	5280	D (*sea-sak-scn*)	Jiangsu	2016	Comparative fitness assay; stress-phenotype assay; cytotoxicity assay; animal infection assay
SKLX6805	ST239	ST239-I	III (3A)	t037	0	0	0	0	1	1	1	5130	D (*sea-sak-scn*)	Zhejiang	2014	Comparative fitness assay; stress-phenotype assay; cytotoxicity assay; animal infection assay
SKLX115984	ST239	ST239-I	III (3A)	t037	0	0	0	0	0	0	1	5280	D (*sea-sak-scn*)	Shandong	2019	Cytotoxicity assay; animal infection assay
SKLX126254	ST239	ST239-I	III (3A)	t421	0	0	0	0	1	1	1	5280	D (*sea-sak-scn*)	Jiangsu	2020	Cytotoxicity assay; animal infection assay
SKLX126255	ST239	ST239-I	III (3A)	t421	0	0	0	0	1	1	1	5280	D (*sea-sak-scn*)	Jiangsu	2020	Cytotoxicity assay; animal infection assay
SKLX20178	ST239	ST239-I	III (3A)	t037	0	0	0	0	1	1	1	5280	E (*sak-scn*)	Hebei	2011	Cytotoxicity assay; animal infection assay
SKLX20395	ST239	ST239-I	III (3A)	t037	0	0	0	0	1	1	1	5280	Negative	Zhejiang	2011	Cytotoxicity assay; animal infection assay
SKLX20422	ST239	ST239-I	III (3A)	t037	0	0	0	0	1	1	1	5280	D (*sea-sak-scn*)	Zhejiang	2012	Cytotoxicity assay; animal infection assay
SKLX20580	ST239	ST239-I	III (3A)	t421	0	0	0	0	1	1	1	5280	D (*sea-sak-scn*)	Shandong	2012	Cytotoxicity assay; animal infection assay
SKLX20595	ST239	ST239-I	III (3A)	t037	0	0	0	0	1	1	1	5280	D (*sea-sak-scn*)	Shandong	2011	Cytotoxicity assay; animal infection assay
SKLX20599	ST239	ST239-I	III (3A)	t421	0	0	0	0	1	1	1	5280	D (*sea-sak-scn*)	Shandong	2012	Cytotoxicity assay; animal infection assay
SKLX20848	ST239	ST239-I	III (3A)	t074	0	0	0	0	1	1	1	5280	E (*sak-scn*)	Guangdong	2012	Cytotoxicity assay; animal infection assay
SKLX21636	ST239	ST239-I	III (3A)	t037	0	0	0	0	1	1	1	5280	D (*sea-sak-scn*)	Gansu	2011	Cytotoxicity assay; animal infection assay
SKLX21656	ST239	ST239-I	III (3A)	t037	0	0	0	0	1	1	1	5280	D (*sea-sak-scn*)	Gansu	2011	Cytotoxicity assay; animal infection assay
SKLX3282	ST239	ST239-I	III (3A)	t037	0	0	0	0	1	1	1	5280	Negative	Guangdong	2011	Cytotoxicity assay; animal infection assay

SKLX43239	ST239	ST239-I	III (3A)	t037	0	0	0	0	1	1	1	5280	D (*sea-sak-scn*)	Zhejiang	2016	Cytotoxicity assay; animal infection assay
SKLX59537	ST239	ST239-I	III (3A)	t037	0	0	0	0	1	1	1	5280	Negative	Fujian	2016	Cytotoxicity assay; animal infection assay
SKLX116294	ST239	ST239-II	III (3A)	t030	0	0	0	0	1	1	1	5280	D (*sea-sak-scn*)	Liaoning	2019	Comparative fitness assay; stress-phenotype assay; cytotoxicity assay; animal infection assay
SKLX119013	ST239	ST239-II	III (3A)	t233	0	0	0	0	1	1	1	5280	D (*sea-sak-scn*)	Ningxia	2019	Comparative fitness assay; stress-phenotype assay; cytotoxicity assay; animal infection assay
SKLX29848	ST239	ST239-II	III (3A)	t030	0	0	0	0	1	1	1	5319	D (*sea-sak-scn*)	Zhejiang	2015	Comparative fitness assay; stress-phenotype assay; cytotoxicity assay; animal infection assay
SKLX51240	ST239	ST239-II	III (3A)	t030	0	0	0	0	1	1	1	5280	D (*sea-sak-scn*)	Anhui	2014	Comparative fitness assay; stress-phenotype assay; cytotoxicity assay; animal infection assay
SKLX54320	ST239	ST239-II	III (3A)	t030	0	0	0	0	0	0	1	5280	D (*sea-sak-scn*)	Anhui	2015	Comparative fitness assay; stress-phenotype assay; cytotoxicity assay; animal infection assay
SKLX54561	ST239	ST239-II	III (3A)	t632	0	0	0	0	1	1	1	5280	D (*sea-sak-scn*)	Shandong	2015	Comparative fitness assay; stress-phenotype assay; cytotoxicity assay; animal infection assay
SKLX56193	ST239	ST239-II	III (3A)	t030	0	0	0	0	1	1	1	5280	D (*sea-sak-scn*)	Hubei	2016	Comparative fitness assay; stress-phenotype assay; cytotoxicity assay; animal infection assay
SKLX60806	ST239	ST239-II	III (3A)	t030	0	0	0	0	0	0	1	5280	D (*sea-sak-scn*)	Gansu	2017	Comparative fitness assay; stress-phenotype assay; cytotoxicity assay; animal infection assay
SKLX62563	ST239	ST239-II	III (3A)	t632	0	0	0	0	1	1	1	5280	D (*sea-sak-scn*)	Shandong	2017	Comparative fitness assay; stress-phenotype assay; cytotoxicity assay; animal infection assay
SKLX83686	ST239	ST239-II	III (3A)	t030	0	0	0	0	1	1	1	5280	D (*sea-sak-scn*)	Anhui	2018	Comparative fitness assay; stress-phenotype assay; cytotoxicity assay; animal infection assay
SKLX122951	ST239	ST239-II	III (3A)	t030	0	0	0	0	1	1	1	5280	D (*sea-sak-scn*)	Shaanxi	2020	Cytotoxicity assay; animal infection assay
SKLX144797	ST239	ST239-II	III (3A)	t030	0	0	0	0	0	0	1	5280	D (*sea-sak-scn*)	Shandong	2020	Cytotoxicity assay; animal infection assay
SKLX16424	ST239	ST239-II	III (3A)	t030	0	0	0	0	1	1	1	5280	D (*sea-sak-scn*)	Zhejiang	2014	Cytotoxicity assay; animal infection assay
SKLX20174	ST239	ST239-II	III (3A)	t030	0	0	0	0	1	1	1	5280	D (*sea-sak-scn*)	Hebei	2011	Cytotoxicity assay; animal infection assay
SKLX20413	ST239	ST239-II	III (3A)	t030	0	0	0	0	1	1	1	5280	D (*sea-sak-scn*)	Zhejiang	2012	Cytotoxicity assay; animal infection assay

SKLX20855	ST239	ST239-II	III (3A)	t030	0	0	0	0	1	1	1	5280	D (*sea-sak-scn*)	Guangdong	2012	Cytotoxicity assay; animal infection assay
SKLX21333	ST239	ST239-II	III (3A)	t030	0	0	0	0	1	1	1	5280	D (*sea-sak-scn*)	Yunnan	2011	Cytotoxicity assay; animal infection assay
SKLX21353	ST239	ST239-II	III (3A)	t030	0	0	0	0	1	1	1	5280	D (*sea-sak-scn*)	Yunnan	2012	Cytotoxicity assay; animal infection assay
SKLX21621	ST239	ST239-II	III (3A)	t030	0	0	0	0	0	0	1	5280	D (*sea-sak-scn*)	Gansu	2011	Cytotoxicity assay; animal infection assay
SKLX21628	ST239	ST239-II	III (3A)	t030	0	0	0	0	1	1	1	5280	D (*sea-sak-scn*)	Gansu	2011	Cytotoxicity assay; animal infection assay
SKLX21640	ST239	ST239-II	III (3A)	t030	0	0	0	0	0	0	1	5280	D (*sea-sak-scn*)	Gansu	2011	Cytotoxicity assay; animal infection assay
SKLX21878	ST239	ST239-II	III (3A)	t030	0	0	0	0	1	1	1	5280	Negative	Zhejiang	2012	Cytotoxicity assay; animal infection assay
SKLX58116	ST239	ST239-II	III (3A)	t030	0	0	0	0	0	0	1	5280	D (*sea-sak-scn*)	Xinjiang	2016	Cytotoxicity assay; animal infection assay
SKLX60032	ST239	ST239-II	III (3A)	t030	0	0	0	0	1	1	1	5280	D (*sea-sak-scn*)	Ningxia	2016	Cytotoxicity assay; animal infection assay
SKLX99159	ST239	ST239-II	III (3A)	t030	0	0	0	0	0	0	1	5319	Negative	Zhejiang	2019	Cytotoxicity assay; animal infection assay

*For comparative fitness (growth/competition assays) and stress-phenotype assays (oxidative stress, desiccation, thermotolerance, high osmolarity and acid tolerance), we randomly selected 20 ST59 isolates (4 per clade, I-V) and 20 ST239 isolates (10 per clade, I-II) to represent genomic diversity.

For cytotoxicity (THP-1 LDH) assays, all ST59 (n = 401) and all ST239 (n = 184) isolates were tested.

For animal infection models, we used panels of 50 ST59 and 50 ST239 clinical isolates. For ST59, we included the 20 isolates above (4 per clade, I-V) plus an additional 30 (6 per clade), yielding 10 per clade (I-V). For ST239, we included the 20 isolates above (10 per clade, I-II) plus an additional 30 (15 per clade), yielding 25 per clade (I-II).

For mutant construction and complementation, *chp* and *sraP* were deleted and restored in the representative ST59 clinical isolate SKLX15777 (SCC*mec* IVa; *spa* type t437), which displays high cytotoxicity (88.07% THP-1 lysis) and carries intact *chp* and *sraP*, enabling clear phenotypic contrasts. This isolate was also used for transcriptomic profiling during exponential growth under acidic (pH 4.5) and physiological (pH 7.4) conditions.

Table S4. Isolate-specific (diagnostic) SNPs for isolates used in competition experiments.

Strain	ST	Clade	Diagnostic SNP	Position*
SKLX144763	ST59	ST59-I	A	150383
			T	183048
			A	193831
			A	420812
			T	434097
			G	462364
			T	467333
			G	521080
			G	563478
			T	575253
			C	692170
			C	789974
			T	876121
			T	907221
			T	981083
			T	1004954
			T	1010936
			G	1032725
			G	1040958
			T	1062394
			T	1109527
			A	1122511
			A	1139480
			A	1247703
			A	1273882
			T	1288015
			A	1300801
			A	1329472
			T	1342789
			T	1361807
			G	1363597
			A	1473790
			G	1499625
			A	1500254
			A	1598280
			A	1608713

			C	1721135
			A	1785842
			A	1860170
			C	1921053
			A	1938706
			T	1949009
			T	2012165
			A	2089765
			T	2347153
			T	2349907
			T	2415104
			C	2452291
			A	2484911
			A	2491289
			G	2506748
			A	2532390
			A	2556069
			A	2566711
			G	2676413
			C	2686431
			C	2709786
			T	2730231
			A	2764059
			A	2844019
			A	2859856
			A	2975007
			A	2981835
			T	2985635
			A	2993010
			T	3042304
SKLX55046	ST59	ST59-I	A	152696
			T	174689
			A	174690
			T	174693
			G	174694
			T	219580
			A	279945
			T	301816

			T	601635
			T	681493
			A	684974
			T	824340
			T	939342
			T	1065330
			T	1093553
			C	1266861
			T	1330121
			T	1344549
			T	1449632
			T	1511232
			C	1711445
			T	1802125
			C	1904743
			T	2093881
			G	2344751
			A	2480704
			C	2531986
			A	2600757
			T	2657121
			T	2734511
			T	2786374
			A	2846205
			T	2969282
			T	2999048
SKLX84856	ST59	ST59-I	A	76860
			G	171718
			A	312112
			G	355567
			T	433629
			T	472233
			A	572440
			C	603082
			G	672872
			T	700562
			C	718599
			A	939537

			A	977284
			T	1051041
			A	1097253
			A	1177947
			T	1444423
			A	1470607
			G	1483481
			A	1500850
			G	1532792
			C	1584713
			A	1822933
			T	1908075
			A	1938006
			T	2029273
			A	2072579
			C	2099021
			A	2173169
			A	2173170
			A	2179245
			C	2312210
			T	2344639
			A	2419164
			T	2556247
			A	2562007
			C	2573011
			A	2576043
			T	2628830
			T	2696158
			C	2869433
			A	2904765
SKLX94337	ST59	ST59-I	G	9439
			G	146358
			A	155625
			T	218556
			A	278447
			A	293518
			G	320319
			A	328655

T	420692
A	437589
T	438920
C	610044
G	660202
A	689341
G	693334
T	694537
A	701897
G	706173
A	720149
T	793209
G	871308
G	978641
A	984200
C	1001859
A	1012838
T	1083635
T	1117572
A	1131904
T	1155293
G	1162339
T	1172267
A	1261347
A	1273498
A	1284169
G	1344803
T	1367032
A	1397806
T	1397807
A	1397810
C	1566612
T	1607439
A	1627871
A	1656939
A	1683439
A	1702676
G	1708331

			T	1708775
			C	1760856
			T	1786262
			A	1918829
			A	1918830
			T	2007926
			G	2015677
			A	2068115
			T	2071330
			T	2097231
			G	2177661
			A	2356381
			A	2402301
			C	2413629
			G	2438383
			G	2457746
			T	2583038
			A	2647874
			T	2653781
			T	2657468
			A	2657482
			G	2657499
			T	2674778
			T	2712317
			A	2771083
			T	2775765
			A	2856598
			A	2933868
			A	2996336
SKLX117483	ST59	ST59-II	A	14539
			G	118849
			A	150790
			A	163738
			T	176491
			A	213460
			A	216078
			T	230110
			C	250926

C	282639
T	323869
A	424190
A	610465
G	619115
G	626597
T	627187
G	632434
T	638118
T	646288
C	681697
A	684990
G	711069
C	733570
T	767582
T	779329
C	815372
C	849318
G	884766
C	915735
T	915766
A	1018313
A	1018434
G	1083646
A	1115366
A	1138238
C	1140163
T	1172322
T	1172326
G	1284128
G	1340659
C	1362451
T	1362953
A	1408645
G	1430889
G	1440630
T	1479271
T	1489091

A	1500439
A	1527631
A	1541682
G	1599923
T	1750101
T	1800663
G	1811217
C	1812192
A	1839665
C	1861020
T	1913135
T	1921654
T	1921901
A	1942893
C	1944146
T	2009094
G	2016101
T	2021252
T	2104091
C	2109241
T	2382822
G	2407430
C	2426713
G	2430300
G	2475380
A	2483559
G	2486706
G	2507512
C	2538469
A	2654894
T	2670072
A	2684330
T	2742385
T	2748897
T	2761093
T	2787918
G	2951468
A	2992714

			C	3035197
SKLX126793	ST59	ST59-II	T	3127
			T	133870
			T	134410
			T	136287
			A	156603
			T	166342
			C	172131
			T	216392
			T	218401
			G	250496
			A	372928
			T	459874
			G	530286
			A	563896
			A	589665
			T	592919
			A	597222
			T	620272
			T	638974
			T	648349
			G	655142
			A	683586
			A	712365
			T	718887
			C	734878
			A	782249
			T	822050
			T	830719
			T	888056
			G	892028
			C	927447
			A	933669
			T	939584
			A	999170
			G	1010308
			T	1017919
			A	1040840

G	1087555
A	1093879
T	1111464
A	1134013
T	1136255
G	1191384
A	1231858
A	1241086
T	1243293
G	1243956
G	1254797
C	1302678
C	1343750
G	1366958
G	1377548
C	1392480
C	1415307
G	1442772
A	1467075
A	1474618
T	1540221
T	1594013
G	1595181
A	1604372
T	1608359
A	1841143
G	1856549
C	1876311
A	1941590
T	2020244
A	2072150
T	2312119
T	2328051
T	2334230
A	2372601
A	2379267
T	2394885
A	2413695

			T	2424844
			T	2426192
			G	2524425
			A	2530923
			A	2661417
			T	2698009
			A	2775310
			A	2841070
			A	2856700
			G	2868484
			T	2950550
			T	3028766
SKLX57651	ST59	ST59-II	C	1851
			T	7929
			A	161413
			G	172287
			A	176382
			T	229902
			T	243888
			T	246883
			C	322442
			G	339722
			T	344556
			T	374664
			T	473717
			G	563183
			A	589776
			G	594596
			G	604551
			A	705408
			T	734356
			C	740802
			G	746830
			A	800466
			T	829649
			T	844452
			A	886497
			A	893297

T	897657
A	902541
A	973102
C	976788
T	1007837
A	1015043
G	1015044
T	1045956
A	1046500
C	1052416
G	1057777
A	1059434
A	1087294
A	1090599
T	1108257
A	1191774
C	1195374
A	1243611
A	1278563
A	1289247
T	1375267
A	1438167
A	1441368
T	1460798
G	1493182
A	1508847
T	1561944
A	1563389
C	1567421
T	1588327
G	1599179
A	1613833
A	1654594
T	1672851
T	1685162
T	1717586
G	1749399
C	1811530

A	1872275
T	1892180
C	1921160
G	2001678
C	2015783
G	2024749
T	2117204
A	2119178
A	2122408
C	2122429
T	2122432
T	2122512
G	2122513
A	2122534
G	2122558
A	2122576
G	2122588
C	2122591
C	2122597
C	2122612
A	2122615
G	2122617
C	2122621
G	2122627
C	2122666
C	2122696
C	2122705
C	2122711
C	2122714
C	2122753
T	2157039
A	2315780
T	2363940
G	2404033
T	2426234
T	2527466
T	2552979
A	2567499

			G	2595238
			T	2606291
			T	2620307
			T	2653814
			A	2809392
			A	2825610
			T	2948417
			A	2992542
			T	3042324
SKLX97572	ST59	ST59-II	T	30945
			G	117792
			T	133506
			T	199144
			G	212919
			G	213105
			A	214848
			C	220974
			C	263977
			T	285381
			G	294054
			C	302585
			A	339330
			T	374543
			T	375290
			T	422549
			G	544829
			C	560427
			G	607646
			T	624388
			T	648559
			C	721251
			T	782177
			G	913070
			T	921469
			A	972434
			T	980142
			T	1035926
			A	1037837

T	1052004
A	1056200
C	1075126
T	1112137
A	1127607
A	1139778
G	1151602
A	1192612
T	1226024
G	1258273
C	1336131
T	1354283
A	1359009
T	1372000
A	1379256
A	1389032
C	1399626
A	1423978
A	1438742
A	1463211
C	1524095
A	1542189
A	1562971
C	1585567
A	1602920
T	1604829
C	1671786
T	1728553
A	1743521
G	1800770
A	1892974
T	1986798
T	1995849
A	2015232
T	2041805
T	2096824
T	2103879
A	2157943

			A	2310293
			T	2408516
			A	2409216
			T	2472497
			A	2477973
			G	2543254
			A	2551300
			C	2608378
			A	2629634
			G	2673301
			T	2718188
			A	2728906
			G	2732129
			T	2763541
			A	2801296
			T	2859796
			T	2861528
			G	2861529
			A	2861530
			T	2861534
			G	2861535
			T	2862085
			C	2923850
			A	3024278
SKLX58137	ST59	ST59-III	T	275898
			T	277729
			A	365583
			T	483725
			A	640038
			T	659068
			C	723348
			G	739448
			T	770420
			A	771924
			T	788098
			T	803343
			C	899409
			G	981537

			T	1006347
			T	1026404
			T	1047423
			G	1255252
			T	1257174
			A	1284088
			G	1337978
			A	1386860
			A	1407209
			T	1587927
			A	1711380
			A	1711381
			A	1874228
			T	1881637
			T	1885595
			T	1901650
			C	1988942
			T	2021280
			A	2028691
			G	2064302
			T	2508715
			G	2541507
			T	2541508
			T	2567240
			C	2567766
			A	2582160
			A	2605796
			T	2612308
			C	2620051
			A	2652671
			C	2844481
			T	2846976
			T	2944589
			G	2971019
SKLX65332	ST59	ST59-III	T	132058
			T	143253
			T	171539
			G	370782

G	462393
G	464230
G	476708
T	576125
C	593681
T	636545
G	667897
T	684891
C	735031
G	776297
T	787226
A	803998
C	810411
G	836057
C	1008660
A	1103647
A	1140248
A	1155268
G	1210334
A	1229997
A	1265171
T	1276105
T	1311413
A	1356754
T	1397819
G	1447550
T	1512777
G	1541401
T	1572254
C	1627042
T	1627826
T	1682236
A	1696357
G	1925398
T	2015096
A	2044382
A	2071020
A	2079071

			T	2469029
			A	2485481
			A	2541241
			T	2552289
			A	2597335
			C	2603695
			T	2652654
			G	2681348
			C	2698467
			C	2775782
			T	2792929
			A	2825737
			C	2832382
			A	2847434
			T	2913781
			T	2931066
			A	2951434
			G	3028274
SKLX83809	ST59	ST59-III	G	117326
			A	124783
			T	162735
			G	175000
			T	176455
			C	199362
			T	253473
			G	361890
			G	363741
			G	562255
			A	674923
			G	675253
			A	766953
			T	858955
			G	862185
			G	912498
			G	917898
			T	951142
			A	1042622
			A	1084838

			A	1090545
			A	1142194
			T	1280585
			C	1350736
			C	1363137
			G	1372075
			G	1400531
			A	1433431
			C	1460892
			G	1561897
			T	1588346
			A	1656311
			T	1656918
			G	1752847
			C	1808126
			T	1872441
			T	2021377
			A	2073098
			A	2309199
			A	2363326
			C	2363347
			T	2575356
			T	2612116
			A	2672142
			A	2725683
			G	2788010
			A	2805459
			T	2995710
SKLX88607	ST59	ST59-III	A	191193
			T	222957
			T	238608
			A	292278
			A	292803
			T	440944
			T	555808
			A	636641
			A	643479
			C	652411

A	672355
G	697823
T	736716
A	737138
G	802604
T	834620
T	910752
A	926896
G	969669
T	989113
A	1033626
A	1037268
G	1051359
A	1070650
C	1112746
T	1121674
G	1128917
T	1132516
G	1245213
T	1256949
G	1293167
T	1342414
A	1346519
T	1394908
A	1397765
T	1417301
T	1426543
A	1487903
G	1539873
G	1591229
T	1648213
A	1662832
T	1687618
C	1702758
A	1727592
G	1727593
C	1727594
T	1764486

			A	1788321
			A	1801230
			A	1839450
			T	1939809
			A	2039692
			T	2066497
			A	2319373
			A	2324002
			C	2334221
			A	2382369
			G	2405058
			A	2510959
			C	2583380
			T	2653739
			G	2706548
			T	2757636
			G	2790786
			T	2804390
			G	2819352
			T	2831508
			C	2843864
			A	2853486
			C	2947307
			T	3024273
SKLX125955	ST59	ST59-IV	C	22325
			T	74657
			C	150843
			T	177878
			C	186969
			C	213872
			G	214821
			A	222762
			A	233489
			A	260057
			T	320722
			A	323818
			C	364507
			A	443207

T	457386
T	480420
A	609152
G	611141
A	626449
A	633568
A	637087
C	645056
G	651704
A	680757
T	714656
G	760513
A	766919
T	780861
A	819142
A	823673
G	868781
T	918683
T	995307
T	1020195
T	1056556
T	1100874
T	1166946
A	1176972
C	1189307
T	1203449
A	1203987
T	1232738
T	1290859
G	1312405
T	1395961
A	1415801
A	1442880
A	1463921
A	1470156
T	1497618
C	1508775
G	1517637

G	1529909
A	1567508
C	1605156
A	1612879
T	1672679
T	1723433
C	1765453
A	1801783
T	1823078
T	1827154
A	1829206
T	1845985
T	1870224
T	1941670
A	1996470
G	2039040
T	2040814
G	2088146
G	2101276
A	2117364
T	2181283
A	2320332
G	2425142
A	2458597
A	2482772
T	2487267
T	2612596
A	2659018
A	2682536
G	2693211
A	2746267
A	2752691
G	2763984
A	2768249
A	2807227
A	2820876
C	2824646
A	2841216

			A	2855160
			T	2855411
			G	2923597
			A	2941588
			T	2944803
			A	2950681
			T	2989520
SKLX61303	ST59	ST59-IV	A	19760
			G	196067
			T	228814
			A	273003
			A	324236
			C	374746
			T	434440
			C	463913
			C	472393
			A	531323
			A	540083
			G	551909
			A	560607
			T	697492
			T	779732
			G	789558
			T	797451
			A	818421
			G	938291
			T	1002699
			T	1030075
			C	1041709
			G	1097660
			T	1119674
			G	1171553
			T	1300573
			A	1307941
			C	1367413
			T	1387798
			A	1454764
			A	1528193

			G	1543373
			A	1561929
			G	1668623
			C	1730424
			T	1743983
			T	1764790
			T	1843803
			T	1892655
			T	2016710
			T	2059723
			T	2168872
			G	2334589
			A	2337072
			G	2340592
			A	2361639
			C	2390674
			A	2426728
			T	2532817
			C	2541010
			T	2607889
			T	2677965
			C	2688472
			T	2729448
			T	2770581
			A	2835048
			T	2981444
SKLX63908	ST59	ST59-IV	G	429
			T	14612
			C	140549
			C	196208
			T	213806
			A	236349
			G	251349
			G	269632
			G	269637
			T	322297
			A	322331
			C	421676

T	421822
A	429421
A	445969
T	465662
T	521707
C	581480
T	614759
G	687755
T	754254
T	760283
C	799105
G	861196
C	871002
G	933653
A	988980
A	1026312
A	1039207
A	1040826
G	1050825
A	1082712
G	1084139
A	1173405
T	1258413
C	1381131
C	1472965
C	1478558
T	1492975
T	1530567
T	1540777
A	1542872
C	1543226
A	1627924
A	1638420
G	1688348
A	1791976
C	1816851
G	1834048
C	1836635

			C	1839732
			A	1873821
			T	1876601
			G	1913137
			A	2026654
			T	2027158
			G	2027557
			C	2043295
			C	2073499
			A	2081403
			A	2083825
			C	2174937
			G	2349909
			T	2360528
			A	2394924
			A	2452303
			T	2459506
			G	2555322
			T	2609521
			T	2691947
			T	2762058
			G	2762493
			G	2762518
			A	2762519
			C	2762520
			G	2766354
			A	2784560
			T	2798114
			T	2826309
			G	2842996
			G	2863759
			A	2872126
			A	2944544
			T	2950532
			G	2982222
SKLX77782	ST59	ST59-IV	T	77126
			A	120905
			A	156594

G	186775
C	208259
A	274794
T	281875
A	288366
A	341429
A	539806
T	660079
T	663359
T	671380
A	747619
A	753898
T	788425
G	843837
G	880673
A	993523
C	1053412
T	1075999
A	1077375
C	1175110
T	1317594
T	1352934
T	1389232
T	1474607
A	1540305
A	1595410
T	1638490
A	1658648
T	1685291
C	1691649
A	1695099
T	1749801
G	1823182
T	1927917
T	1946564
A	2012389
G	2080421
C	2352311

			C	2372846
			T	2396689
			A	2432075
			A	2450309
			A	2526595
			T	2574656
			C	2658019
			T	2685963
			A	2694316
			T	2703178
			A	2711203
			C	2807228
			G	2816994
			A	2828256
			T	2918761
			A	2949279
			C	2981074
			A	2984578
SKLX15777	ST59	ST59-V	T	157147
			C	274178
			T	286991
			G	358813
			T	428939
			T	465727
			T	522293
			G	620612
			G	627169
			T	751287
			A	911386
			C	975993
			C	1129032
			C	1141377
			T	1217086
			T	1270143
			A	1285447
			T	1406308
			T	1427117
			A	1437633

			T	1477439
			G	1505549
			T	1519994
			T	1600911
			G	1645492
			G	1720775
			T	1725751
			A	1728380
			A	1787102
			T	1827757
			G	1852253
			G	1900187
			G	2109139
			T	2110552
			C	2117588
			A	2117912
			T	2118363
			C	2145755
			A	2344124
			T	2435743
			A	2526592
			T	2558910
			G	2570031
			A	2600059
			A	2634880
			A	2745556
			T	2765510
			A	2895440
			A	2994932
SKLX39485	ST59	ST59-V	T	2115
			A	13602
			T	122315
			A	187829
			T	203419
			C	236111
			A	330920
			G	338797
			T	356928

G	358952
G	366441
T	447976
A	469979
A	477394
A	477399
C	525278
A	547485
A	593408
G	602858
A	619567
T	737764
A	746053
G	766116
T	782011
A	782674
T	878384
T	1005567
C	1110430
T	1110431
A	1110437
C	1164056
C	1165964
C	1320231
A	1321382
C	1336924
T	1357030
G	1398751
A	1423600
C	1431752
G	1490523
T	1544104
T	1569700
A	1576110
T	1604779
C	1727776
T	1745633
C	2007447

			T	2156571
			A	2171385
			A	2179984
			C	2365492
			T	2450543
			T	2466591
			G	2514072
			T	2594286
			G	2607415
			C	2610685
			G	2642385
			C	2682510
			A	2808899
			G	2847614
			T	2933361
			A	2937564
			G	2947505
			C	2952622
			T	2982993
			G	3035822
			A	3042107
SKLX52911	ST59	ST59-V	T	151557
			T	282694
			G	299486
			A	306769
			A	312554
			C	319874
			G	348879
			C	424958
			G	444487
			A	470626
			G	537277
			C	563584
			T	569956
			G	580667
			C	582288
			G	597578
			C	608710

A	689261
A	690608
A	759571
T	776167
T	821940
A	833852
T	852295
T	862529
T	872798
C	899375
G	992482
T	992483
C	1009512
A	1019833
A	1023592
G	1024981
C	1059859
A	1085801
A	1100494
C	1116581
A	1116586
G	1116587
A	1116599
T	1116602
T	1123228
G	1170435
C	1199534
A	1203733
A	1203734
T	1215087
A	1240343
A	1276137
T	1283934
G	1315363
G	1320498
C	1333716
A	1340385
C	1360311

A	1364271
G	1374790
T	1412634
T	1419603
C	1486996
G	1492337
C	1493582
A	1514345
T	1530633
C	1532965
C	1576189
A	1585166
T	1600131
A	1632579
C	1661284
T	1762002
A	1765884
T	1789642
G	1804977
G	1852319
C	1853942
C	1872714
G	1928363
C	1984519
A	2008604
G	2014241
A	2014724
T	2032178
T	2135064
A	2325547
A	2344249
T	2353689
C	2392906
C	2404085
T	2434899
A	2476848
G	2478024
C	2505821

			C	2535619
			T	2541509
			T	2541510
			T	2545367
			A	2568227
			C	2586086
			G	2619645
			A	2653368
			C	2672166
			T	2673520
			G	2692878
			C	2754495
			A	2759679
			G	2771608
			T	2798285
			C	2816927
			A	2904999
			A	2953177
			G	2968799
SKLX85887	ST59	ST59-V	G	172225
			G	209882
			A	257000
			T	316296
			A	331959
			G	444093
			T	444094
			G	444095
			A	472304
			C	558782
			A	571276
			A	698996
			A	705426
			T	766365
			G	807308
			G	841031
			A	874280
			G	935943
			G	937372

T	976263
T	981445
T	1009175
T	1012966
T	1051553
A	1085722
A	1087064
T	1095599
G	1107060
T	1195157
T	1306308
G	1316675
C	1358462
G	1449074
C	1567537
T	1570129
T	1580025
C	1801260
T	1834528
G	1842790
G	1855681
T	1872343
G	1872461
G	1888736
T	1921581
A	1943640
C	2026901
C	2035074
T	2078037
T	2569985
T	2588313
T	2597178
C	2666422
C	2674869
A	2678004
T	2703809
A	2704659
A	2736142

			T	2751995
			C	2813031
			A	2819059
			G	2904488
			T	2916059
			G	2939113
			C	2985523
			T	2996556
SKLX115943	ST239	ST239-I	T	10320
			T	78419
			T	136850
			T	149646
			G	191532
			C	230728
			C	234727
			G	254923
			C	269445
			A	269451
			T	269455
			C	269457
			G	269460
			T	284439
			C	329324
			T	329871
			A	330675
			T	447304
			A	544913
			A	553924
			G	559167
			T	572825
			T	574235
			T	578670
			A	579946
			T	593914
			T	594099
			A	611120
			A	617272
			T	626593

C	649513
G	654992
C	689290
C	699440
T	723111
A	749599
G	752413
T	766350
A	805754
A	808657
G	819087
T	878813
G	883953
T	902088
C	904540
G	934978
T	967072
A	977452
T	989538
A	1052316
C	1123852
T	1133137
G	1187393
A	1192971
A	1195696
A	1220867
C	1251506
A	1257877
A	1264653
A	1328507
T	1370479
T	1383285
T	1390661
G	1397249
A	1407220
T	1426236
A	1429068
G	1448146

T	1453148
T	1465160
G	1494078
T	1522589
A	1604588
C	1675298
C	1690547
A	1707887
G	1785072
T	1803781
C	1849486
C	1873507
C	1918527
C	1936720
T	1988650
T	2028262
A	2081873
C	2175057
A	2312195
A	2341080
T	2348736
G	2385345
A	2393618
T	2406731
T	2434219
T	2508571
T	2509083
C	2512954
A	2513087
C	2519647
C	2527844
G	2543164
C	2547688
A	2570175
A	2591581
A	2596959
T	2626428
A	2662897

			T	2680993
			A	2683591
			A	2710406
			T	2711523
			A	2726984
			T	2804625
			A	2843770
			A	2844955
			G	2865155
			C	2905479
			A	2939892
			G	2942623
			C	2942635
			A	2966359
			A	2970301
			T	2990213
			A	2995775
			A	3042546
SKLX126248	ST239	ST239-I	C	25238
			G	29305
			T	116730
			G	124655
			G	137880
			A	200286
			C	231837
			G	265413
			C	283707
			A	288086
			G	576025
			G	584753
			A	605973
			C	632475
			T	632485
			T	632495
			T	633110
			G	673629
			T	776239
			A	776613

T	799185
T	1026556
T	1031293
A	1142362
A	1164643
C	1166286
G	1169064
T	1176927
C	1180306
C	1211284
A	1275032
C	1280265
C	1302022
A	1326320
C	1407069
G	1427141
C	1467623
G	1482157
C	1499419
A	1639867
A	1646367
T	1677652
T	1789643
C	1818974
C	1829675
C	1857030
C	1995407
G	2027572
A	2030046
A	2032886
T	2046641
C	2327955
A	2368855
C	2392058
T	2403529
G	2414629
T	2491476
T	2573853

			C	2590427
			A	2602709
			C	2712108
			A	2719429
			A	2724266
			C	2746241
			A	2781812
			C	2781830
			T	2827784
			A	2830089
			G	2838562
			A	2942686
			T	2942687
			G	2953663
			G	2959530
SKLX39166	ST239	ST239-I	G	239876
			A	260722
			A	277268
			A	278375
			T	290403
			A	334187
			T	361171
			T	367687
			C	447058
			A	571315
			T	610017
			T	617080
			C	651129
			A	665423
			C	666537
			A	671536
			T	712994
			T	771016
			C	846582
			T	866196
			G	900069
			T	937353
			T	942661

A	942780
C	1012420
T	1013954
A	1040808
C	1056956
A	1087020
G	1104665
G	1139476
A	1158594
T	1268206
G	1296974
T	1316832
T	1333823
G	1335090
C	1360895
T	1363380
T	1413023
T	1415123
A	1415466
A	1432072
G	1491697
C	1528268
T	1545207
C	1561912
A	1561913
A	1579192
T	1648275
C	1664685
T	1675394
G	1680162
G	1682482
T	1689170
A	1726905
G	1782749
C	1810413
A	1838561
A	1924589
T	1925630

			A	2039563
			T	2057048
			C	2067768
			C	2154074
			A	2155671
			T	2313031
			A	2399902
			A	2420947
			A	2454214
			T	2559667
			A	2602658
			A	2622721
			A	2670236
			C	2691047
			T	2692818
			A	2802255
			A	2840683
			A	2844687
			T	2854543
			A	2909298
			T	2916508
			A	2922859
			C	2926361
			G	2950599
			A	3024798
			T	3028217
SKLX42156	ST239	ST239-I	A	6956
			A	32509
			T	163517
			G	207623
			A	213894
			A	215360
			G	269747
			T	291540
			T	368351
			A	454549
			T	578105
			T	579732

G	580323
T	754714
C	888249
T	891595
C	906199
T	946177
T	1001794
T	1094472
A	1129233
T	1236435
C	1245981
C	1256312
G	1303714
C	1315215
T	1384206
G	1462433
A	1475713
A	1480176
A	1532872
A	1610319
A	1638012
T	1705932
T	1716197
A	1725986
G	1763063
T	1833813
A	1900763
C	2003572
T	2326399
T	2341704
C	2411766
A	2412992
T	2455338
T	2533122
T	2671012
T	2798027
T	2909479
C	2947743

			C	2967500
			A	3029973
SKLX44458	ST239	ST239-I	G	31446
			C	305906
			A	340384
			A	473688
			T	629081
			A	795072
			C	807591
			T	894337
			C	977541
			T	1002007
			G	1040889
			C	1347221
			A	1348122
			C	1353587
			G	1393143
			A	1463215
			T	1523605
			C	1527945
			C	1608801
			C	1738702
			G	1838992
			A	1857312
			G	1860022
			G	1904417
			A	2051994
			A	2335319
			T	2421349
			A	2434415
			C	2485600
			T	2559908
			T	2577489
			G	2598763
			T	2619286
			T	2622424
			G	2677012
			T	2803444

			C	2895974
			A	3022685
			C	3024659
SKLX53417	ST239	ST239-I	C	118839
			T	146917
			T	202284
			T	206346
			C	214671
			G	266054
			T	279840
			A	420786
			C	458416
			C	459869
			A	523044
			A	568176
			T	636054
			G	754405
			T	782086
			A	814322
			A	869160
			T	899324
			T	939119
			G	969457
			A	976771
			T	995783
			C	1037506
			A	1082861
			C	1090146
			G	1093697
			C	1139639
			G	1147191
			A	1197122
			A	1230944
			A	1230947
			T	1258525
			C	1260929
			A	1289666
			A	1405659

			T	1407227
			T	1434598
			C	1461893
			G	1462339
			G	1501256
			G	1557325
			G	1559699
			G	1606414
			C	1643315
			C	1757236
			C	1757542
			G	1830340
			C	1858241
			A	1871464
			G	1887463
			A	1995621
			T	2180420
			G	2319951
			T	2568648
			G	2663527
			A	2677239
			A	2753642
			A	2770172
			C	2782753
			G	2786316
			G	2808169
			T	2857552
			T	2930423
			C	2961870
SKLX54652	ST239	ST239-I	A	12564
			A	31937
			G	123037
			A	150144
			T	162390
			T	179031
			G	181150
			G	201018
			C	238716

C	334084
A	425961
G	437367
A	533722
T	576320
C	614875
T	650175
A	656882
A	768019
C	781276
T	831920
T	876427
G	918277
T	977200
G	989439
A	995041
A	1036988
C	1079201
T	1111405
A	1113775
G	1115747
A	1147694
A	1218715
A	1234812
A	1245851
T	1256945
A	1265198
C	1308920
C	1325062
C	1417098
T	1462678
A	1481364
A	1490068
T	1530465
A	1601159
A	1608915
T	1633557
A	1638878

			G	1667659
			C	1670786
			C	1774454
			T	1826794
			T	2064230
			C	2358752
			T	2526211
			A	2541529
			C	2541539
			A	2578679
			G	2656220
			T	2729736
			C	2749390
			G	2763263
			T	2786917
			T	2898830
			T	2900087
			A	2922689
			T	3022109
			T	3030417
			T	3040573
SKLX56296	ST239	ST239-I	A	3636
			T	33492
			A	126646
			G	142633
			T	175471
			A	199001
			T	213595
			G	248295
			G	254021
			T	283908
			G	294670
			A	316372
			C	368682
			A	370936
			G	447427
			C	476368
			A	539250

A	545906
C	548854
T	624480
T	660947
C	667036
A	701658
T	713939
A	765844
C	773990
C	779312
C	787836
T	802183
T	822332
T	822664
A	845327
T	854456
G	863704
A	888550
A	892309
A	902606
T	974575
C	1009443
G	1018594
A	1049516
G	1075566
T	1195360
A	1298968
G	1313718
A	1331230
A	1353261
G	1365904
A	1370000
C	1373903
A	1404280
A	1420948
T	1433322
C	1448065
T	1478393

T	1512804
T	1542716
C	1560551
T	1579004
C	1633947
G	1654214
T	1685743
T	1696473
C	1705317
T	1705469
C	1804410
T	1838114
G	1868437
A	1927221
T	1942252
A	2024808
A	2037739
T	2063479
C	2066571
T	2094182
T	2102198
T	2117659
A	2156835
C	2310365
A	2333722
G	2359775
A	2389990
C	2417049
A	2473561
C	2490224
A	2495608
A	2533124
C	2537607
T	2567865
A	2577532
A	2647919
A	2686134
C	2757614

			T	2772066
			T	2859927
			A	2862754
			T	2897047
SKLX57440	ST239	ST239-I	T	6264
			T	142612
			A	236176
			T	304481
			T	357268
			C	485532
			T	579063
			T	804312
			T	844894
			A	886877
			A	890397
			A	891670
			A	936166
			C	1159816
			A	1346306
			C	1579086
			C	1668681
			A	1708788
			A	1728197
			T	1740814
			T	1851182
			T	1928185
			T	2035324
			G	2094309
			G	2104032
			C	2570403
			G	2602311
			T	2652395
			A	2696990
			C	2784186
			T	2790346
			T	2791422
			C	2859932
SKLX6805	ST239	ST239-I	G	185852

A	197439
T	231751
C	263509
T	263552
C	544704
T	550462
G	551220
A	668530
C	815400
T	843991
A	977798
T	977799
G	1010973
T	1077929
G	1093907
C	1143733
A	1175368
G	1195164
C	1223848
T	1384495
G	1415114
A	1510477
A	1577306
T	1654125
C	1683796
T	1687655
A	1699735
A	1711556
T	1781881
C	1781885
A	1781888
C	1781889
T	1781890
A	1829219
T	1836578
T	1892263
A	2030976
C	2058088

			A	2088859
			C	2339843
			G	2377425
			G	2450202
			C	2485470
			A	2579235
			C	2585164
			G	2585535
			G	2632655
			G	2637128
			T	2766135
			G	2768856
			C	2852377
			T	2917242
			C	2951029
			A	2953203
			A	2981273
			C	2991554
			G	3028089
SKLX116294	ST239	ST239-II	G	32822
			T	125613
			A	174157
			A	184737
			G	195072
			G	234681
			T	237697
			G	335460
			T	355111
			A	557270
			G	569576
			T	606829
			G	625035
			G	657696
			A	659938
			C	663919
			T	666366
			T	666538
			T	751032

T	754307
A	758337
T	769914
G	777190
G	787066
A	818687
G	869971
C	927494
A	948703
C	1031816
T	1033786
A	1058944
C	1112098
T	1130087
G	1136704
T	1139654
G	1151653
A	1199837
T	1214902
A	1246197
T	1275257
G	1310225
G	1427556
C	1494183
C	1495957
A	1497721
T	1499963
T	1500829
C	1504173
T	1569707
G	1606345
C	1617770
C	1688667
T	1716602
G	1728792
T	1739540
A	1756672
G	1775099

			G	1809104
			T	1828687
			G	1833782
			G	1919761
			A	1995522
			A	2041559
			T	2109520
			G	2390618
			G	2402825
			G	2463977
			C	2485519
			T	2496349
			A	2536947
			T	2537859
			C	2674309
			T	2742442
			C	2768560
			G	2816953
			C	2818928
			A	2822307
			G	2914033
SKLX119013	ST239	ST239-II	T	75240
			C	75242
			G	75274
			T	75276
			C	75279
			A	75298
			G	133744
			T	217113
			A	240044
			T	240366
			C	261124
			T	264949
			C	271948
			T	291650
			T	296371
			A	374017
			T	460587

T	482239
G	528684
A	591326
A	646588
C	769377
A	796549
T	942570
T	1002015
G	1007314
C	1033172
T	1046866
A	1059510
A	1065095
T	1097242
A	1106653
A	1106682
T	1108203
A	1116387
G	1125384
A	1140246
C	1233719
T	1257220
C	1300240
T	1378041
G	1390577
G	1474176
T	1503721
C	1513264
T	1515757
A	1571827
C	1676915
C	1682780
A	1686634
A	1710172
A	1750574
A	1761902
A	1801211
A	1808230

			T	1823397
			T	1877636
			T	1944184
			A	1995082
			C	2020555
			C	2028917
			T	2094185
			G	2105385
			T	2180906
			A	2337582
			T	2361703
			T	2400095
			T	2439096
			T	2453313
			A	2454479
			T	2458101
			A	2479955
			C	2490214
			G	2542113
			A	2550027
			T	2557103
			A	2672895
			A	2675766
			T	2715486
			T	2726020
			T	2832984
			G	2839065
			A	2851662
			G	2865915
			A	2897376
			A	2938555
			C	2941949
			G	2941954
SKLX29848	ST239	ST239-II	A	2115
			A	3095
			A	122807
			T	143662
			A	185642

A	213007
C	219709
A	293853
A	305934
A	319304
C	343180
A	421903
C	463911
A	550272
C	603243
A	644708
T	647381
A	661078
G	701677
G	751316
G	756930
T	768894
T	857919
G	863843
G	875636
G	908716
A	917381
T	920761
T	927361
A	981085
A	1003399
A	1085659
C	1100558
A	1114665
C	1189340
C	1195563
A	1275487
A	1349699
A	1367698
G	1397185
G	1420920
C	1459957
T	1511345

			T	1524566
			C	1527577
			A	1531604
			A	1590396
			A	1675356
			A	1699262
			T	1783934
			C	1784741
			T	1842739
			A	1889479
			G	1892506
			C	1917025
			A	1922206
			T	1932912
			G	2027635
			T	2372423
			A	2398606
			T	2476063
			A	2492406
			T	2528700
			T	2538833
			T	2567169
			T	2583608
			C	2638853
			T	2667603
			T	2735122
			A	2756661
			T	2760752
			T	2865256
			T	2969018
SKLX51240	ST239	ST239-II	A	78397
			G	147904
			A	151408
			G	220949
			T	240323
			T	372856
			T	549089
			T	576734

			T	627073
			T	754618
			A	774896
			T	869900
			G	942931
			A	1002016
			C	1032492
			A	1121998
			A	1126614
			T	1253570
			A	1347776
			T	1367525
			C	1405709
			T	1582387
			T	1624188
			T	1677717
			C	1763933
			C	1791796
			T	1880164
			C	1905194
			T	2051713
			T	2344396
			A	2509689
			G	2513616
			T	2544939
			T	2569471
			T	2639614
			G	2775216
			A	2793252
			A	2843091
			C	2856482
			T	2861551
			C	2903625
SKLX54320	ST239	ST239-II	G	164
			T	234182
			A	263372
			A	431892
			T	556670

			G	621998
			A	622009
			C	735807
			C	742246
			C	811522
			C	926810
			G	1051494
			T	1164132
			T	1220401
			G	1498759
			T	1527616
			G	1540735
			A	1657468
			T	1720048
			A	1738467
			T	1774710
			C	1824626
			T	1848690
			A	1856483
			G	1943505
			C	1988739
			G	2034400
			T	2039447
			A	2067774
			G	2101571
			C	2104847
			T	2339584
			G	2435183
			G	2435936
			G	2518488
			G	2581906
			A	2600555
			C	2684115
			C	2831038
			A	2842856
SKLX54561	ST239	ST239-II	T	259689
			C	436911
			T	450377

			A	453459
			G	477438
			A	574133
			A	600674
			G	777189
			G	1320036
			A	1361335
			C	1479262
			A	1885549
			G	2087658
			C	2479693
			A	2568620
			T	2745194
			A	2909384
SKLX56193	ST239	ST239-II	C	181105
			C	579325
			G	609645
			G	633612
			T	655078
			C	705745
			C	1032933
			A	1126593
			C	1160878
			T	1206385
			A	1356256
			G	1405690
			A	1415504
			G	1476222
			G	1560457
			T	1700672
			C	1706745
			A	1793443
			A	1797330
			C	1802693
			A	1869246
			A	1875087
			T	1913436
			A	1923354

			A	2062684
			A	2176992
			G	2390001
			T	2412349
			C	2415394
			T	2438196
			A	2461984
			A	2478774
			A	2491493
			G	2519344
			T	2525775
			T	2551070
			T	2695362
			A	2710680
			T	2789615
			C	2967831
SKLX60806	ST239	ST239-II	A	14629
			T	25703
			A	127612
			G	194290
			A	197711
			T	234909
			A	271403
			T	299442
			A	365123
			A	423897
			C	424366
			C	480950
			T	558298
			C	644483
			C	655970
			T	712788
			C	748916
			A	818699
			C	870787
			C	915613
			T	976331
			T	1137122

A	1167428
T	1180751
T	1187717
T	1209732
C	1273567
C	1318669
G	1348480
A	1348491
G	1354264
T	1441946
T	1487796
A	1579726
A	1586132
T	1594562
T	1629673
G	1691639
A	1744943
A	1752317
T	1758356
T	1762735
C	1887212
G	1919140
A	2060378
C	2061119
T	2118987
C	2177322
A	2333727
A	2337518
G	2401678
C	2466892
T	2525020
G	2586965
T	2627590
A	2640630
A	2647021
A	2649588
T	2736094
C	2805589

			A	2839475
			G	2934434
			C	2965559
			G	2989666
SKLX62563	ST239	ST239-II	T	32844
			A	345852
			T	433989
			C	565252
			A	598584
			G	605862
			G	646655
			G	655703
			T	796644
			A	812468
			G	857672
			T	867892
			A	877173
			C	1100576
			T	1115278
			A	1133045
			T	1158055
			T	1225887
			A	1232991
			G	1249151
			A	1344444
			G	1392892
			C	1480456
			A	1547337
			G	1572368
			G	1606513
			T	1629633
			A	1669473
			C	1681809
			G	1715505
			G	1774664
			G	1810944
			T	1911688
			C	1939151

			T	2044957
			A	2053300
			A	2067724
			C	2418718
			T	2451244
			A	2617309
			C	2640147
			A	2752265
			T	2816456
			A	2928164
			T	2933334
			C	2945721
			G	2974322
			C	3023719
			A	3040458
SKLX83686	ST239	ST239-II	A	12830
			A	120037
			A	212212
			C	332964
			G	473112
			T	567466
			A	589447
			T	608961
			G	645542
			T	891910
			A	898615
			T	946424
			C	1066941
			T	1070463
			T	1116386
			T	1391358
			C	1521496
			A	1627180
			A	1654216
			C	1725649
			T	1760898
			C	1988161
			T	2050362

A	2073701
T	2084297
T	2168836
C	2316567
A	2366580
A	2425352
T	2440180
C	2485480
T	2508205
A	2553953
T	2578229
C	2628790
A	2638758
T	2673715
A	2702324
C	2844069
A	2849565
G	2962230
A	2989007

*Positions are reported relative to the ST239 reference TW20 reference genome (GenBank accession FN433596.1).
